# Mycophenolic Acid Derivatives with Immunosuppressive Activity from the Coral-Derived Fungus *Penicillium bialowiezense*

**DOI:** 10.3390/md16070230

**Published:** 2018-07-07

**Authors:** Qing Zhang, Beiye Yang, Fengli Li, Mengting Liu, Shuang Lin, Jianping Wang, Yongbo Xue, Hucheng Zhu, Weiguang Sun, Zhengxi Hu, Yonghui Zhang

**Affiliations:** Hubei Key Laboratory of Natural Medicinal Chemistry and Resource Evaluation, School of Pharmacy, Tongji Medical College, Huazhong University of Science and Technology, Wuhan 430030, China; zhq_em@163.com (Q.Z.); yangbeiye123@163.com (B.Y.); 13971267286@163.com (F.L.); tjliumengting@163.com (M.L.); 13207186519@163.com (S.L.); jpwang1001@163.com (J.W.); yongboxue@mail.hust.edu.cn (Y.X.); zhuhucheng0@163.com (H.Z.)

**Keywords:** coral-derived fungus, *Penicillium bialowiezense*, Mycophenolic acid derivatives, immunosuppressive activity, molecular docking

## Abstract

Mycophenolic acid (MPA) is a potent inosine-5′-monophosphate dehydrogenase (IMPDH) inhibitor for immunosuppressive chemotherapy. Most importantly, as the 2-morpholinoethyl ester prodrug of MPA, mycophenolate mofetil (MMF) is a well-known immunosuppressant used to prevent rejection in organ transplantations. Nevertheless, due to its frequently occurred side effects, searching for new therapeutic agents is ongoing. In our current work, by virtue of efficient bioassay-guided fractionation and purification, eleven mycophenolic acid derivatives, including five previously unreported metabolites (**3**–**7**) and six known compounds (**1**, **2**, and **8**–**11**), were obtained from the coral-derived fungus *Penicillium bialowiezense*. Their structures were elucidated by means of extensive spectroscopic analyses (including 1D and 2D NMR and HRESIMS data) and comparison of the NMR and other physical data with those reported in the literature in the case of the known compounds. All the isolates **1**–**11** were evaluated for the immunosuppressive activity, and **1**–**3** showed potent IMPDH2 inhibitory potency with IC_50_ values of 0.84–0.95 μM, which were comparable to that of MPA (the positive control), while **4**–**10** showed significant inhibitory potency with IC_50_ values of 3.27–24.68 μM. All the MPA derivatives showed promising immunosuppressive activity, endowing them as potential drug leads for organ transplantations and autoimmune related diseases.

## 1. Introduction

With great advantages of unique ecological environments, powerful gene clusters, and high yields of secondary metabolites, marine-derived fungi represent a gigantic and insufficiently untapped reservoir for the exploration of novel bioactive marine natural products (MNPs) [1,2,3]. One promising family of such MNPs is the mycophenolic acid (MPA) family, which was mainly discovered in the *Penicillium* genus, such as *P. brevicompactum*, *P. viridicatum*, *P. griscobrunneum*, *P. stoloniferum*, etc. [4]. Since the first report of MPA in 1893 [5], the MPA and its derivatives have attracted much attention from phytochemists and pharmacologists because of their wide array of bioactivities, such as immunosuppressive, antitumor, antiviral, and RNA capping inhibitory properties [6]. Over the last decades, the studies on MPA and its derivatives are the hotspot fields owing to their potentials to provide natural chemotypes for the discovery of new therapeutic agents; for instance, MPA is a well-known non-competitive and reversible inhibitor of inosine-5′-monophosphate dehydrogenase (IMPDH), which is responsible for regulating the biosynthesis of intracellular guanine nucleotide, and thus is of great importance for the DNA and RNA synthesis, signal transduction, energy source for translation, glycoprotein synthesis, as well as other cellular proliferation processes [7]. More importantly, as the 2-morpholinoethyl ester prodrug of MPA, mycophenolate mofetil (MMF) has been widely used as a clinical immunosuppressant in heart, kidney, lung and liver transplantation processes [8]. However, the frequently occurred side effects such as leukopenia and gastrointestinal disorders, particularly diarrhea restricted the use of MMF in clinical practice [8]. Therefore, the exploration is still on the way for more effective analogues that are non-toxic or of low toxicity and beneficial to improve the quality of life of patients.

**Figure 1:**
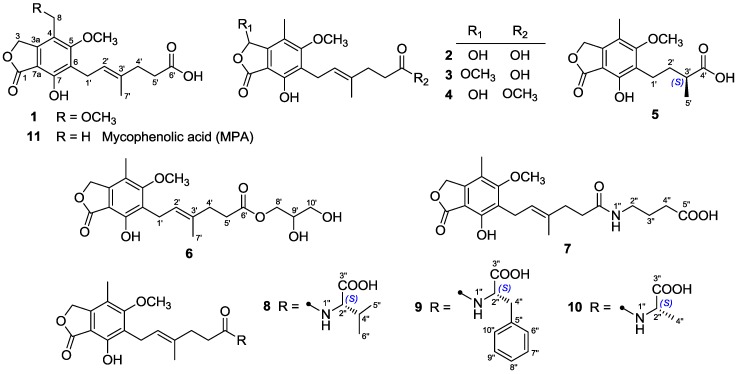
Chemical structures of **1**–**11**.

As part of our program aiming at exploring structurally unique natural products with interesting bioactivities from fungi inhabiting unique environments [9,10,11,12,13,14], a coral-derived fungus *Penicillium bialowiezense* was cultivated in the solid-state fermented rice medium. By virtue of effective bioassay-guided fractionation and purification, eleven mycophenolic acid derivatives, including five previously unreported metabolites (**3**–**7**) and six known compounds (**1**, **2**, and **8**–**11**), were obtained. Herein, we report the isolation, structure elucidation, and immunosuppressive activity for all these compounds (Figure 1).

## 2. Results

Compounds **3** and **4**, both isolated as white powders, gave the identical molecular formula C_18_H_22_O_7_ based on their HRESIMS *m*/*z* 373.1260 [M + Na]^+^ and 373.1282 [M + Na]^+^ (calcd. for C_18_H_22_O_7_Na, 373.1263) as well as the ^13^C-NMR and DEPT data, indicating eight degrees of unsaturation. The IR spectrum of **3** showed absorptions of hydroxyl (3435 cm^−1^), ester carbonyl (1744 cm^−1^), and aromatic ring (1626 and 1456 cm^−1^). The ^1^H-NMR spectrum (Table 1) of **3** showed characteristic signals attributable to two methyl groups at *δ*_H_ 1.81 (3H, s, Me-7′) and 2.20 (3H, s, Me-8), two methoxyl groups at *δ*_H_ 3.54 (3H, s, OMe-3) and 3.75 (3H, s, OMe-5), and one olefinic proton at *δ*_H_ 5.25 (^1^H, t, *J* = 6.9 Hz). The ^13^C-NMR and DEPT spectra (Table 2) showed the presence of 18 carbon signals, including four methyls (two oxygenated), three sp^3^ methylenes, one sp^2^ methine, one oxygen-bearing sp^3^ methine, seven sp^2^ quaternary and two carbonyl carbons. These data suggested that **1** was a MPA derivative.

The ^1^H and ^13^C-NMR data (Table 1 and Table 2) of **3** resembled those of **2**, except for the presence of a methoxyl group (*δ*_H_ 3.54; *δ*_C_ 56.4) in **3** instead of a hydroxyl group on C-3 in **2**, as demonstrated by the HMBC correlation from H-3 (*δ*_H_ 6.34) to the methoxyl carbon (*δ*_C_ 56.4). Comparison of the ^1^H and ^13^C-NMR data (Table 1 and Table 2) of **4** with those of **2** revealed that the C-6′ carboxyl group in **2** was methyl-esterified in **4**, which was confirmed by the HMBC correlation from 6′-OMe (*δ*_H_ 3.60) to the ester carbonyl carbon at *δ*_C_ 174.1. Compounds **3** and **4** were optically inactive since they showed no Cotton effects in the experimental CD spectra, suggesting that they were also racemic. Unfortunately, an attempt to separate the enantiomers of **3** and **4** was not successful. Accordingly, the structures of **3** and **4** were elucidated as 6-(5-carboxy-3-methylpent-2-enyl)-7-hydroxy-3,5-dimethoxy-4-methylphthalan-1-one and 6-(5-methoxycarbonyl-3-methylpent-2-enyl)-3,7-dihydroxy-5-methoxy-4-methylphthalan-1-one, respectively.

**Figure 2:**
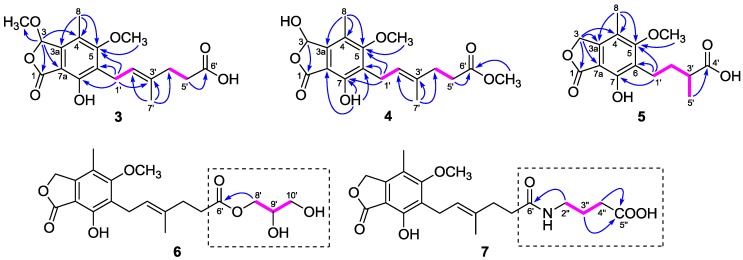
Key ^1^H–^1^H COSY (pink lines) and HMBC (blue arrows) correlations of **3**–**7**.

Compound **5** was purified as a white powder. Its molecular formula C_15_H_18_O_6_ was assigned based on the HRESIMS *m*/*z* 317.1007 [M + Na]^+^ (calcd. for C_15_H_18_O_6_Na, 317.1001). The NMR data of **5** (Table 3) closely resembled those of euparvic acid [15], whose absolute structure was confirmed by X-ray crystallography. The only difference was that the C-5 hydroxy group in euparvic acid was replaced by a methoxyl group (*δ*_H_ 3.79; *δ*_C_ 61.6) in **5**, as supported by the HMBC correlation from the methoxyl proton to C-5 (*δ*_C_ 164.9) (Figure 2). As the specific rotations of **5** {[α${]}_{D}^{23}$: –6.6 (*c* 0.01, MeOH)} and euparvic acid {[*α*${]}_{D}^{20}$: –4.0 (*c* 0.01, MeOH)} were levorotatory, it was suggested that the absolute configuration of C-3′ in **5** should be the same as that of C-3′ of euparvic acid, i.e., 3′*S*. Thus, the structure of **5** was identified as 6-(3-carboxybutyl)-7-hydroxy-5-methoxy-4-methylphthalan-1-one.

Compound **6** was also isolated as a white, amorphous powder. The HRESIMS analysis of **6** showed a positive molecular ion peak at *m*/*z* 417.1495 [M + Na]^+^ (calcd. for C_20_H_26_O_8_Na, 417.1525), corresponding to a molecular formula C_20_H_26_O_8_. Comparison of its ^1^H and ^13^C-NMR data (Table 1 and Table 2) with those of **11** revealed that **6** was 2,3-dihydroxypropyl mycophenolate. This structure was supported by the ^1^H–^1^H COSY correlations from H-8′ through H-10′ and HMBC correlation from H_2_-8′ (*δ*_H_ 3.98 and 4.06) to C-6′ (*δ*_C_ 174.8) (Figure 2). The specific rotation of **6** was approximately zero, indicating that **6** was also a racemic mixture. Thus, compound **6** was named 6-[5-(2,3-dihydroxy-1-carboxyglyceride)-3-methylpent-2-enyl]-7-hydroxy-5-methoxy-4-methylphthalan-1-one.

The molecular formula of **7** was assigned as C_21_H_27_NO_7_ from the HRESIMS *m*/*z* 428.1708 [M + Na]^+^ (calcd. for C_21_H_27_NO_7_Na, 428.1685). The ^1^H and ^13^C-NMR data of **7** (Table 1 and Table 2) were similar to those of **6**, except for the presence of a 4-aminobutanoic acid moiety instead of the 2,3-dihydroxypropyl group. This hypothesis was confirmed by the ^1^H–^1^H COSY correlations from H-2″ through H-4″ and HMBC correlations from H_2_-2″ (*δ*_H_ 3.11) to C-6′ (*δ*_C_ 175.8) and from H_2_-3″ (*δ*_H_ 1.68) and H_2_-4″ (*δ*_H_ 2.24) to C-5″ (*δ*_C_ 177.2) (Figure 2). Thus, the structure of **7** was elucidated as 6-[5-(1-carboxy-4-*N*-carboxylate)-3-methylpent-2-enyl]-7-hydroxy-5-methoxy-4-methylphthalan-1-one.

Compounds **1**–**2** and **8**–**11** were identified as 8-*O*-methyl mycophenolic acid (**1**) [16], 3-hydroxy mycophenolic acid (**2**) [16], *N*-mycophenoyl-l-valine (**8**) [8], *N*-mycophenoyl-l-phenyloalanine (**9**) [8], *N*-mycophenoyl-l-alanine (**10**) [8], and MPA (**11**) [17] by comparison of their NMR data and specific rotations with those reported in the literature. Moreover, their structures were confirmed by ^1^H–^1^H COSY and HMBC correlations (for detailed structural determination, please see the Supporting Information).

To assess the biological activity of the MPA derivatives, we primarily evaluated their IMPDH2 inhibitory activity with the previously reported enzyme assay method [18]. The results (Table 4) revealed that all these compounds could inhibit IMPDH2 with IC_50_ values ranging from 0.59 to 24.68 µM, of which compounds **1**–**3** were comparable to that of MPA (the positive control), suggesting that hydroxylation or methoxylation of the main skeleton (7-hydroxy-5-methoxy-4-methylphthalan-1-one moiety) minimally affected the IMPDH2 inhibition, while esterification of hexenoic acid tail dramatically decreased the IMPDH2 inhibitory activity, which was in accordance with that of mycophenolate mofetil (MMF) [19]. Subsequently, the MPA derivatives were further tested for the in vitro immunosuppressive activity against the proliferation of T lymphocytes. Unsurprisingly, the activity at the cellular level was markedly consistent with their IMPDH2 inhibitory activity.

**Figure 3:**
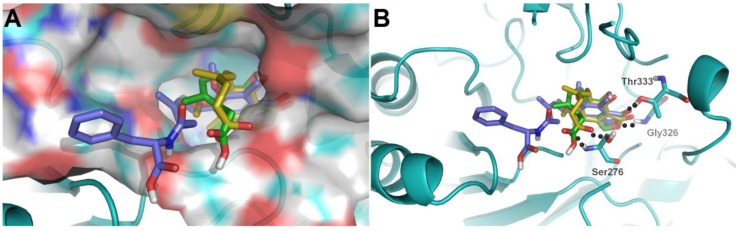
(**A**) Low-energy binding conformations of compounds **3**, **9**, and MPA (**11**) bound to IMPDH2 enzyme generated by virtual ligand docking; (**B**) Compounds **3**, **9**, and MPA (**11**) are depicted as the stick model showing carbon (green, blue, and yellow, respectively), hydrogen (grey), oxygen (red). The black balls represent the hydrogen bonds.

To further investigate their structure-activity relationships and modes of action, the binding modes and docking scores of compounds with IMPDH2 were obtained by molecular docking [20]. As shown in Figure 3, compounds **3** and **9** with considerable different levels on the activeness of enzyme were chosen to compare with MPA. The main skeleton was deeply buried into the binding pocket with several hydrogen bonds between these compounds and IMPDH2 observed, which included hydrogen bonds between the lactone oxygen (O-2) and the amide nitrogen of Gly326, and the carbonyl (C-1) oxygen and hydroxyl group of Thr333. Moreover, the hexenoic acid tail of **3** and MPA could adopt a bent conformation which allowed the carboxylate group to form hydrogen bonds with the amide nitrogen and side-chain hydroxyl groups of Ser276, thus the collective data from both bioassays and virtual docking revealed the importance of the hydrogen interactions in the immunosuppressive activity displayed by the MPA scaffold at both the enzymatic and cellular levels.

## 3. Materials and Methods

### 3.1. General Experimental Procedures

Optical rotations were measured by using a PerkinElmer 341 instrument. UV and FT-IR spectra were recorded by using a Varian Cary 50 and a Bruker Vertex 70 instrument, respectively. High-resolution electrospray ionization mass spectrometry (HRESIMS) were performed by using the positive ion mode with a Thermo Fisher LC-LTQ-Orbitrap XL spectrometer. The 1D (^1^H, ^13^C, DEPT) and 2D (HSQC, HMBC, ^1^H–^1^H COSY) NMR spectra were recorded by using Bruker AM-400 and DRX-600 instruments with tetramethylsilane as an internal standard, and the chemical shifts (*δ*) were expressed in ppm and referenced to the solvent signals (*δ*_H_ 3.31 and *δ*_C_ 49.0 for CD_3_OD). Semi-preparative HPLC were performed by using an Agilent 1200 liquid chromatograph with a Zorbax SB-C_18_ (9.4 mm × 25 cm) column. Column chromatography (CC) was performed by using Silica gel (200–300 mesh; Qingdao Marine Chemical, Inc., Qingdao, China), Sephadex LH-20 (GE Healthcare Bio-Sciences AB, Uppsala, Sweden), and Lichroprep RP-C_18_ gel (40–63 μm, Merck, Darmstadt, Germany). Analytical Thin-layer chromatography (TLC) was performed with precoated silica gel 60 F_254_ glass plates (200–250 μm thickness, Qingdao Marine Chemical Inc.), and spots were visualized by spraying heated silica gel plates with 10% H_2_SO_4_ in EtOH.

### 3.2. Fungal Material

The strain *Penicillium bialowiezense* was isolated from the fresh the soft coral *Sarcophyton subviride*, which was collected from the Xisha Island (16°45′ N, 111°65′ E) in the South China Sea in October 2016. The strain was authenticated by one of the authors (J. Wang), according to its morphology and sequence analysis of the ITS region of the rDNA (GenBank accession MH443003). The fungal sample was preserved in the culture collection center of Tongji Medical College, Huazhong University of Science and Technology.

### 3.3. Fermentation, Extraction, and Bioassay-Guided Isolation Procedures

The strain *Penicillium bialowiezense* was incubated on potato dextrose agar (PDA) medium at 26 °C for 7 days in stationary phase to prepare the seed cultures, which were then cut into small pieces (nearly 0.4 × 0.4 × 0.4 cm) and inoculated into 300 × 500 mL Erlenmeyer flasks (compositions: 200 g rice and 200 mL distilled water), previously sterilized by autoclaving. All flasks were incubated at 28 °C for 40 days. Following incubation, the growth of cells was stopped by adding 300 mL EtOAc to each flask, and the culture was homogenized. The fermented materials were extracted with EtOAc (30 L) for eight times, and under reduced pressure the organic solvent was evaporated to dryness to give a dark brown crude extract (600 g), which showed a moderate immunosuppressive activity against the proliferation of T lymphocytes at a concentration of 33.6 ± 0.41 μg/mL.

The total extract (600 g) was subjected to silica gel CC (100–200 mesh), eluted with a gradient of petroleum ether–EtOAc–MeOH (20:1:0 to 1:1:1) to give eight fractions (Fr.1–Fr.8), which were evaluated for immunosuppressive activity against the proliferation of T lymphocytes. As a result, only Fr.4 (petroleum ether–EtOAc, 2:1) and Fr.5 (petroleum ether–EtOAc, 1:1) showed obvious inhibitory activity at concentrations of 1.42 ± 0.15 and 11.68 ± 0.83 μg/mL, respectively. Then, Fr.4 (140 g) was subjected to YMC RP-C_18_ CC (MeOH–H_2_O, 20% to 100%) to give five subfractions (Fr.4.1–Fr.4.5). Compound **11** (50 g) crystallized from Fr.4.3 (MeOH–H_2_O, 60%, 100 g), and the residue was loaded onto Sephadex LH-20 (CH_2_Cl_2_–MeOH, 1:1), silica gel CC (CH_2_Cl_2_–MeOH, 60:1), and repeated semi-preparative HPLC (MeOH–H_2_O, 70:30, 2.0 mL/min) to yield compounds **1** (7.9 mg), **2** (17.3 mg), **3** (20.5 mg), **4** (4.2 mg), and **5** (11.3 mg). Fr.5 (100 g) was separated into five subfractions by RP-C_18_ CC (MeOH–H_2_O, 20% to 100%). Fr.5.3 (MeOH–H_2_O, 60%, 65 g) was further purified by Sephadex LH-20 (CH_2_Cl_2_–MeOH, 1:1), silica gel CC using CH_2_Cl_2_–MeOH (stepwise 50:1 to 30:1), and repeated semi-preparative HPLC eluted with MeOH–H_2_O (65:35, 2 mL/min) or CH_3_CN–H_2_O (60:40, 2 mL/min) to afford compounds **6** (10.2 mg), **7** (10.4 mg), **8** (9.2 mg), **9** (21.6 mg), and **10** (8.5 mg).

6-(5-Carboxy-3-methylpent-2-enyl)-7-hydroxy-3,5-dimethoxy-4-methylphthalan-1-one (**3**): white powder; UV (MeOH) λ_max_ (log *ε*): 219 (4.48), 253 (3.75), and 309 (3.57) nm; IR (*ν*_max_): 3435, 2923, 2853, 1744, 1626, 1456, 1416, 1382, 1208, 1138, 1073, 908, 676 cm^−1^; HRESIMS *m*/*z* 373.1260 [M + Na]^+^ (calcd. for C_18_H_22_O_7_Na, 373.1263); ^1^H and ^13^C-NMR data, see Table 1 and Table 2.

6-(5-Methoxycarbonyl-3-methylpent-2-enyl)-3,7-dihydroxy-5-methoxy-4-methylphthalan-1-one (**4**): white powder; UV (MeOH) λ_max_ (log *ε*): 218 (4.50), 251 (3.79), and 311 (3.64) nm; IR (*ν*_max_): 3442, 2922, 2852, 1739, 1643, 1466, 1440, 1382, 1278, 1093, 683 cm^−1^; HRESIMS *m*/*z* 373.1282 [M + Na]^+^ (calcd. for C_18_H_22_O_7_Na, 373.1263); ^1^H and ^13^C-NMR data, see Table 1 and Table 2.

6-(3-Carboxybutyl)-7-hydroxy-5-methoxy-4-methylphthalan-1-one (**5**): white powder; [α${]}_{D}^{23}$: –6.6 (*c* 0.01, MeOH); UV (MeOH) λ_max_ (log *ε*): 217 (4.35), 252 (3.71), and 307 (3.39) nm; IR (*ν*_max_): 3423, 2925, 2854, 1732, 1619, 1462, 1413, 1378, 1322, 1196, 1141, 1034, 967, 678 cm^−1^; HRESIMS *m*/*z* 317.1007 [M + Na]^+^ (calcd. for C_15_H_18_O_6_Na, 317.1001); ^1^H and ^13^C-NMR data, see Table 3.

6-[5-(2,3-Dihydroxy-1-carboxyglyceride)-3-methylpent-2-enyl]-7-hydroxy-5-methoxy-4-methylphthalan-1-one (**6**): white powder; [α${]}_{D}^{23}$: 0 (*c* 0.5, MeOH); UV (MeOH) λ_max_ (log *ε*): 217 (4.24), 251 (3.47), and 306 (3.15) nm; IR (*ν*_max_): 3396, 2922, 2851, 1736, 1646, 1456, 1415, 1382, 1271, 1137, 1080, 1033, 972, 647 cm^−1^; HRESIMS *m*/*z* 417.1495 [M + Na]^+^ (calcd. for C_20_H_26_O_8_Na, 417.1525); ^1^H and ^13^C-NMR data, see Table 1 and Table 2.

6-[5-(1-Carboxy-4-*N*-carboxylate)-3-methylpent-2-enyl]-7-hydroxy-5-methoxy-4-methylphthalan-1-one (**7**): white powder; UV (MeOH) λ_max_ (log *ε*): 217 (4.46), 251 (3.72), and 306 (3.43) nm; IR (*ν*_max_): 3420, 2924, 2853, 1736, 1633, 1454, 1412, 1377, 1328, 1276, 1193, 1136, 1078, 1030, 970, 792, 652, 597 cm^−1^; HRESIMS *m*/*z* 428.1708 [M + Na]^+^ (calcd. for C_21_H_27_NO_7_Na, 428.1685); ^1^H and ^13^C-NMR data, see Table 1 and Table 2.

### 3.4. Biological Assays

Each compound was first dissolved in DMSO and then diluted with distilled water to the desired concentration.

#### 3.4.1. IMPDH2 Expression and Purification

The gene encoding IMPDH2 was cloned into the pET-28a vector (Novagen). After the recombinant plasmids were verified by sequencing, the plasmid was transformed into *Escherichia coli* BL21(DE3) (Invitrogen), which was grown in LB medium at 37 °C to an OD_600_ (0.8–1.0) and induced by 0.4 mM isopropyl-D-thiogalactopyranoside (IPTG) and grown at 20 °C for 16 h. The cell pellet was harvested and re-suspended in 30 mL buffer (20 mM Tris pH 8.5, 200 mM NaCl, and 10 mM imidazole), followed by disruption on a French press. Cell debris was removed by centrifugation at 21,000 rpm for 30 min. The supernatant was loaded to Ni-agarose affinity resin, which was washed with buffer A containing 20 mM Tris pH 8.5, 200 mM NaCl, and 10 mM imidazole, and eluted with buffer B containing 20 mM Tris, pH 8.5, 250 mM NaCl, and 150 mM imidazole. The protein was further purified with size exclusion chromatography at 20 mM Tris pH 8.5 and 200 mM NaCl.

#### 3.4.2. Enzyme Inhibition Assay

The IMPDH2 inhibitory effects of test compounds were measured using the recombinant protein above. The enzyme solution (20 µL, 10 µg/mL), test compounds (10 µL) and buffer (40 µL, 100 mM phosphate buffer, pH 7.2) were pipetted and mixed in a 96-well microtiter plate. The mixture was incubated for 10 min at 37 °C. After incubation, inosine 5′-monophosphate (IMP) substrate solution (30 µL, 2.5 mM) and NAD^+^ (30 µL, 5 mM) were added. The reaction can be readily monitored by an increase in optical absorbance at 340 nm. MPA was used as a positive control and averages of three replicates were calculated. The data were imported into Prism (version 5.0, GraphPad) and the IC_50_ values were calculated by using a standard dose response curve fitting.

#### 3.4.3. Immunosuppressive Activity of Test Compounds

BALB/c mice were sacrificed by cervical dislocation, and the spleens were removed aseptically. Mononuclear cell suspensions were prepared after cell debris, and clumps were removed. Erythrocytes were depleted with ammonium chloride buffer solution. Lymphocytes were washed and suspended in DMEM medium supplemented with 10% FBS, penicillin (100 U/mL), and streptomycin (100 mg/mL). The 5 × 10^5^ (150 μL per well) spleen cells in 96-well microtiter plates were cultured at 37 °C in a humidified and 5% CO_2_-containing incubator for 24 h in the presence or absence of the serious concentrations (ranging from 0.1 to 100 µM) of compounds. The cultures were stimulated with 5 μg/mL of concanavalin A (ConA) to induce T cells proliferative response. After treatment for 24 h, the cell quantity was measured using a CCK8 assay kit according to the manufacturer’s instructions.

### 3.5. Molecular Docking Simulation

The virtual docking was implemented in the Surflex-Dock module of the FlexX/Sybyl software, which is a fast docking method that allows sufficient flexibility of ligands and keeps the target protein rigid. Molecules were built with Chemdraw and optimized at molecular mechanical and semiempirical level by using Open Babel GUI. X-ray crystal structure of IMPDH2 at 2.6 Å resolution (PDB ID: 1JR1) was taken from RCSB Protein Data Bank (www.rcsb.org). The crystallographic ligand was extracted from the active site and the designed ligands were modelled. All the hydrogen atoms were added to define the correct ionization and tautomeric states, and the carboxylate, phosphonate, and sulphonate groups were considered in their charged forms. In the docking calculation, the default FlexX scoring function was used for exhaustive searching, solid body optimizing, and interaction scoring. Finally, the ligands with the lowest-energy and the most favorable orientation were selected.

## 4. Conclusions

In conclusion, by virtue of the efficient bioassay-guided isolation, eleven mycophenolic acid derivatives (**1**–**11**), including five previously unreported metabolites (**3**–**7**) and six known compounds (**1**, **2**, and **8**–**11**), were isolated and identified from a coral-derived fungus *Penicillium bialowiezense*. The discovery of **1**–**10** is not only a further structurally supplement to the natural occurring MPAs, but also may be potential chemotaxonomic markers for the *Penicillium* species. Moreover, the immunosuppressive activity of all the isolates were also investigated, and **1**–**3** were found to be quite potent to inhibit IMPDH2 with IC_50_ values ranging from 0.84 to 0.95 μM, which were comparable to that of MPA (the positive control), while **4**–**10** showed significant inhibitory potency with IC_50_ values of 3.27–24.68 μM. These results were consistent with the in vitro immunosuppressive activity against the proliferation of T lymphocytes at the cellular level. Our remarkable work indicates that these bioactive chemicals enable us to excavate the possible utilization of MPA derivatives of their biological functions for human healthcare, especially organ transplantations and autoimmune related diseases.

## Figures and Tables

**Table 1:** ^1^H-NMR data for **3**, **4**, **6**, and **7** in CD_3_OD (*δ* in ppm, *J* in Hz).

No.	3 *^a^*^,*c*^	4 *^b^*^,*c*^	6 *^a^*^,*c*^	7 *^a^*^,*c*^
3	6.34 (1H, s)	6.54 (1H, s)	5.10 (2H, s)	5.24 (2H, s)
8	2.20 (3H, s)	2.25 (3H, s)	2.07 (3H, s)	2.14 (3H, s)
3-OMe	3.54 (3H, s)	-	-	-
5-OMe	3.75 (3H, s)	3.74 (3H, s)	3.72 (3H, s)	3.76 (3H, s)
7-OH	-	7.58 s	-	-
1′	3.39 (2H, br d, *J* = 6.9 Hz)	3.35 (2H, m)	3.28 (2H, br d, *J* = 7.0 Hz)	3.38 (2H, br d, *J* = 7.0 Hz)
2′	5.25 (1H, t, *J* = 6.9 Hz)	5.19 (1H, t, *J* = 6.8 Hz)	5.20 (1H, t, *J* = 7.0 Hz)	5.26 (1H, t, *J* = 7.0 Hz)
4′	2.27 (2H, m)	2.27 (2H, m)	2.24 (2H, m)	2.26 (2H, m)
5′	2.34 (2H, m)	2.37 (2H, m)	2.41 (2H, m)	2.26 (2H, m)
7′	1.81 (3H, s)	1.77 (3H, s)	1.77 (3H, s)	1.82 (3H, s)
8′	-	-	3.98 (1H, dd, *J* = 6.3, 11.3 Hz); 4.06 (1H, dd, *J* = 4.5, 11.3 Hz)	-
9′	-	-	3.78 (1H, m)	-
10′	-	-	3.52 (2H, dd, *J* = 3.3, 5.4 Hz)	-
6′-OMe	-	3.60 (3H, s)	-	-
2″	-	-	-	3.11 (2H, t, *J* = 6.9 Hz)
3″	-	-	-	1.68 (2H, m)
4″	-	-	-	2.24 (2H, m)

*^a^* Recorded at 400 MHz; *^b^* Recorded at 600 MHz; *^c^* “m” means overlapped or multiplet with other signals.

**Table 2:** ^13^C-NMR data for **3**, **4**, **6**, and **7** in CD_3_OD (*δ* in ppm).

No.	3 *^a^*	4 *^b^*	6 *^a^*	7 *^a^*
1	171.0 CO	170.6 CO	173.4 C	173.8 C
3	104.6 CH	98.3 CH	70.6 CH_2_	70.8 CH_2_
3a	143.4 C	142.8 C	146.2 C	146.6 C
4	120.5 C	119.9 C	117.6 C	117.9 C
5	165.2 C	164.4 C	164.4 C	164.8 C
6	126.1 C	124.7 C	123.2 C	123.7 C
7	154.6 C	153.6 C	154.2 C	154.7 C
7a	108.7 C	107.1 C	107.3 C	107.7 C
8	11.1 CH_3_	11.4 CH_3_	11.4 CH_3_	11.4 CH_3_
3-OMe	56.4 CH_3_	-	-	-
5-OMe	61.6 CH_3_	61.2 CH_3_	61.5 CH_3_	61.5 CH_3_
1′	23.8 CH_2_	22.9 CH_2_	23.4 CH_2_	23.6 CH_2_
2′	123.9 CH	122.6 CH	124.2 CH	124.5 CH
3′	135.4 C	134.6 C	134.7 C	135.1 C
4′	36.0 CH_2_	34.8 CH_2_	35.4 CH_2_	36.6 CH_2_
5′	34.3 CH_2_	33.1 CH_2_	33.6 CH_2_	35.8 CH_2_
6′	178.0 C	174.1 C	174.8 C	175.8 C
7′	16.3 CH_3_	16.4 CH_3_	16.2 CH_3_	16.3 CH_3_
8′	-	-	66.3 CH_2_	-
9′	-	-	70.9 CH	-
10′	-	-	63.9 CH_2_	-
6′-OMe	-	51.8 CH_3_	-	-
2″	-	-	-	39.8 CH_2_
3″	-	-	-	25.9 CH_2_
4″	-	-	-	32.5 CH_2_
5″	-	-	-	177.2 C

*^a^* Recorded at 100 MHz; *^b^* Recorded at 150 MHz.

**Table 3:** ^1^H and ^13^C-NMR data for **5** in CD_3_OD (*δ* in ppm, *J* in Hz).

No.	5	
	*δ*_H_^*a*,*b*^	*δ*_C_ *^c^*
1	-	173.8 C
3	5.24 (2H, s)	70.8 CH_2_
3a	-	146.8 C
4	-	117.8 C
5	-	164.9 C
6	-	124.0 C
7	-	154.9 C
7a	-	107.7 C
8	2.15 (3H, s)	11.5 CH_3_
5-OMe	3.79 (3H, s)	61.6 CH_3_
1′	2.71 (2H, m)	22.5 CH_2_
2′	1.65 (1H, m); 1.88 (1H, m)	34.6 CH_2_
3′	2.44 (1H, m)	40.8 CH
4′	-	180.9 C
5′	1.20 (3H, d, *J* = 7.0 Hz)	17.6 CH_3_

*^a^* Recorded at 400 MHz; *^b^* “m” means overlapped or multiplet with other signals; *^c^* Recorded at 100 MHz.

**Table 4:** Summary of IMPDH2 and mouse splenocyte proliferation inhibition assays for **1**–**11**.

Compound	IC_50_ (µM) *^b^* Inosine-5′-Monophosphate Dehydrogenase (IMPDH2)	IC_50_ (µM) *^b^* Mouse Splenocyte Proliferation	Docking Score *^c^*
**1**	0.92 ± 0.08	4.21 ± 0.20	8.04
**2**	0.95 ± 0.09	1.23 ± 0.07	8.21
**3**	0.84 ± 0.11	2.76 ± 0.13	7.83
**4**	3.27 ± 0.18	9.12 ± 0.38	7.12
**5**	24.68 ± 2.74	>40	5.37
**6**	8.59 ± 0.43	19.65 ± 0.89	6.85
**7**	12.64 ± 1.86	24.58 ± 1.34	6.96
**8**	15.73 ± 1.65	30.56 ± 2.19	6.17
**9**	23.76 ± 3.54	>40	5.46
**10**	17.52 ± 1.30	>40	6.28
**MPA (11) *^a^***	0.59 ± 0.08	0.96 ± 0.05	8.15

*^a^* MPA was selected as the positive control; *^b^* Data represent the mean ± SD of three triplicate experiments; *^c^* Interaction potential of compounds with target protein.

## Supplementary Materials

The following are available online at http://www.mdpi.com/1660-3397/16/7/230/s1, 1D & 2D NMR, HRESIMS, UV, and IR spectra of compounds **1**–**10**.
