# Supplementary material for: Mycophenolic Acid Derivatives with Immunosuppressive Activity from the Coral-Derived Fungus Penicillium bialowiezense
Source: Mar Drugs. 2018 Jul 7;16(7):230. doi: 10.3390/md16070230 (PMC6070797; doi:10.3390/md16070230)
Supplement: Supplementary file 1 [file marinedrugs-16-00230-s001.pdf]

Supporting Information

Mycophenolic Acid Derivatives with Immunosuppressive Activity from the Coral-Derived Fungus *Penicillium bialowiezense*

Qing Zhang, Beiye Yang, Fengli Li, Mengting Liu, Shuang Lin, Jianping Wang, Yongbo Xue, Hucheng Zhu, Weiguang Sun*, Zhengxi Hu*, and Yonghui Zhang*

Hubei Key Laboratory of Natural Medicinal Chemistry and Resource Evaluation, School of Pharmacy, Tongji Medical College, Huazhong University of Science and Technology, Wuhan 430030, People's Republic of China

* Corresponding author Tel.: (86) 27-83692892

E-mail: zhangyh@mails.tjmu.edu.cn (Y.Z.); weiguang_s@hust.edu.cn (W.S.); hzx616@126.com (Z.H.)

Contents of Supporting Information

Figure S1. ^{1}H NMR spectrum of compound **1** (Recorded in CD_3OD)........5
Figure S2. ^{13}C NMR spectrum of compound **1** (Recorded in CD_3OD)........6
Figure S3. DEPT spectrum of compound **1** (Recorded in CD_3OD)........7
Figure S4. HSQC spectrum of compound **1** (Recorded in CD_3OD)........8
Figure S5. HMBC spectrum of compound **1** (Recorded in CD_3OD)........9
Figure S6. ^{1}H–^{1}H COSY spectrum of compound **1** (Recorded in CD_3OD)........10
Figure S7. HRESIMS spectrum of compound **1**........11
Figure S8. IR spectrum of compound **1**........12
Figure S9. UV spectrum of compound **1**........13
Figure S10. ^{1}H NMR spectrum of compound **2** (Recorded in CD_3OD)........14
Figure S11. ^{13}C NMR spectrum of compound **2** (Recorded in CD_3OD)........15
Figure S12. DEPT spectrum of compound **2** (Recorded in CD_3OD)........16
Figure S13. HSQC spectrum of compound **2** (Recorded in CD_3OD)........17
Figure S14. HMBC spectrum of compound **2** (Recorded in CD_3OD)........18
Figure S15. ^{1}H–^{1}H COSY spectrum of compound **2** (Recorded in CD_3OD)........19
Figure S16. HRESIMS spectrum of compound **2**........20
Figure S17. IR spectrum of compound **2**........21
Figure S18. UV spectrum of compound **2**........22
Figure S19. ^{1}H NMR spectrum of compound **3** (Recorded in CD_3OD)........23
Figure S20. ^{13}C NMR spectrum of compound **3** (Recorded in CD_3OD)........24
Figure S21. DEPT spectrum of compound **3** (Recorded in CD_3OD)........25
Figure S22. HSQC spectrum of compound **3** (Recorded in CD_3OD)........26
Figure S23. HMBC spectrum of compound **3** (Recorded in CD_3OD)........27
Figure S24. ^{1}H–^{1}H COSY spectrum of compound **3** (Recorded in CD_3OD)........28
Figure S25. HRESIMS spectrum of compound **3**........29
Figure S26. IR spectrum of compound **3**........30
Figure S27. UV spectrum of compound **3**........31
Figure S28. ^{1}H NMR spectrum of compound **4** (Recorded in CD_3OD)........32
Figure S29. ^{13}C NMR spectrum of compound **4** (Recorded in CD_3OD)........33
Figure S30. HSQC spectrum of compound **4** (Recorded in CD_3OD)........34
Figure S31. HMBC spectrum of compound **4** (Recorded in CD_3OD)........35
Figure S32. ^{1}H–^{1}H COSY spectrum of compound **4** (Recorded in CD_3OD)........36
Figure S33. HRESIMS spectrum of compound **4**........37
Figure S34. IR spectrum of compound **4**........38
Figure S35. UV spectrum of compound **4**........39
Figure S36. ^{1}H NMR spectrum of compound **5** (Recorded in CD_3OD)........40

Figure S37. ^{13}C NMR spectrum of compound **5** (Recorded in CD_3OD) 41
Figure S38. DEPT spectrum of compound **5** (Recorded in CD_3OD)..42
Figure S39. HSQC spectrum of compound **5** (Recorded in CD_3OD)..43
Figure S40. HMBC spectrum of compound **5** (Recorded in CD_3OD).......................................44
Figure S41. ^{1}H–^{1}H COSY spectrum of compound **5** (Recorded in CD_3OD) 45
Figure S42. HRESIMS spectrum of compound **5**..46
Figure S43. IR spectrum of compound **5** 47
Figure S44. UV spectrum of compound **5** 48
Figure S45. ^{1}H NMR spectrum of compound **6** (Recorded in CD_3OD).......................................49
Figure S46. ^{13}C NMR spectrum of compound **6** (Recorded in CD_3OD) 50
Figure S47. DEPT spectrum of compound **6** (Recorded in CD_3OD)..51
Figure S48. HSQC spectrum of compound **6** (Recorded in CD_3OD)..52
Figure S49. HMBC spectrum of compound **6** (Recorded in CD_3OD).......................................53
Figure S50. ^{1}H–^{1}H COSY spectrum of compound **6** (Recorded in CD_3OD) 54
Figure S51. HRESIMS spectrum of compound **6**..55
Figure S52. IR spectrum of compound **6** 56
Figure S53. UV spectrum of compound **6** 57
Figure S54. ^{1}H NMR spectrum of compound **7** (Recorded in CD_3OD).......................................58
Figure S55. ^{13}C NMR spectrum of compound **7** (Recorded in CD_3OD) 59
Figure S56. DEPT spectrum of compound **7** (Recorded in CD_3OD)..60
Figure S57. HSQC spectrum of compound **7** (Recorded in CD_3OD)..61
Figure S58. HMBC spectrum of compound **7** (Recorded in CD_3OD).......................................62
Figure S59. ^{1}H–^{1}H COSY spectrum of compound **7** (Recorded in CD_3OD) 63
Figure S60. HRESIMS spectrum of compound **7**..64
Figure S61. IR spectrum of compound **7** 65
Figure S62. UV spectrum of compound **7** 66
Figure S63. ^{1}H NMR spectrum of compound **8** (Recorded in CD_3OD).......................................67
Figure S64. ^{13}C NMR spectrum of compound **8** (Recorded in CD_3OD) 68
Figure S65. DEPT spectrum of compound **8** (Recorded in CD_3OD)..69
Figure S66. HSQC spectrum of compound **8** (Recorded in CD_3OD)..70
Figure S67. HMBC spectrum of compound **8** (Recorded in CD_3OD).......................................71
Figure S68. ^{1}H–^{1}H COSY spectrum of compound **8** (Recorded in CD_3OD) 72
Figure S69. HRESIMS spectrum of compound **8**..73
Figure S70. IR spectrum of compound **8** 74
Figure S71. UV spectrum of compound **8** 75
Figure S72. ^{1}H NMR spectrum of compound **9** (Recorded in CD_3OD).......................................76
Figure S73. ^{13}C NMR spectrum of compound **9** (Recorded in CD_3OD) 77
Figure S74. DEPT spectrum of compound **9** (Recorded in CD_3OD)..78

Figure S75. HSQC spectrum of compound **9** (Recorded in CD_3OD)........79
Figure S76. HMBC spectrum of compound **9** (Recorded in CD_3OD)........80
Figure S77. ^1H–^1H COSY spectrum of compound **9** (Recorded in CD_3OD)........81
Figure S78. HRESIMS spectrum of compound **9**........82
Figure S79. IR spectrum of compound **9**........83
Figure S80. UV spectrum of compound **9**........84
Figure S81. ^1H NMR spectrum of compound **10** (Recorded in CD_3OD)........85
Figure S82. ^{13}C NMR spectrum of compound **10** (Recorded in CD_3OD)........86
Figure S83. DEPT spectrum of compound **10** (Recorded in CD_3OD)........87
Figure S84. HSQC spectrum of compound **10** (Recorded in CD_3OD)........88
Figure S85. HMBC spectrum of compound **10** (Recorded in CD_3OD)........89
Figure S86. ^1H–^1H COSY spectrum of compound **10** (Recorded in CD_3OD)........90
Figure S87. HRESIMS spectrum of compound **10**........91
Figure S88. IR spectrum of compound **10**........92
Figure S89. UV spectrum of compound **10**........93
Structure elucidation of compounds 1, 2, and 8–10........94
The physical and chemical constants of compounds 1, 2, and 8–10........96
^1H and ^{13}C NMR data for compounds 1, 2, and 8–10........97

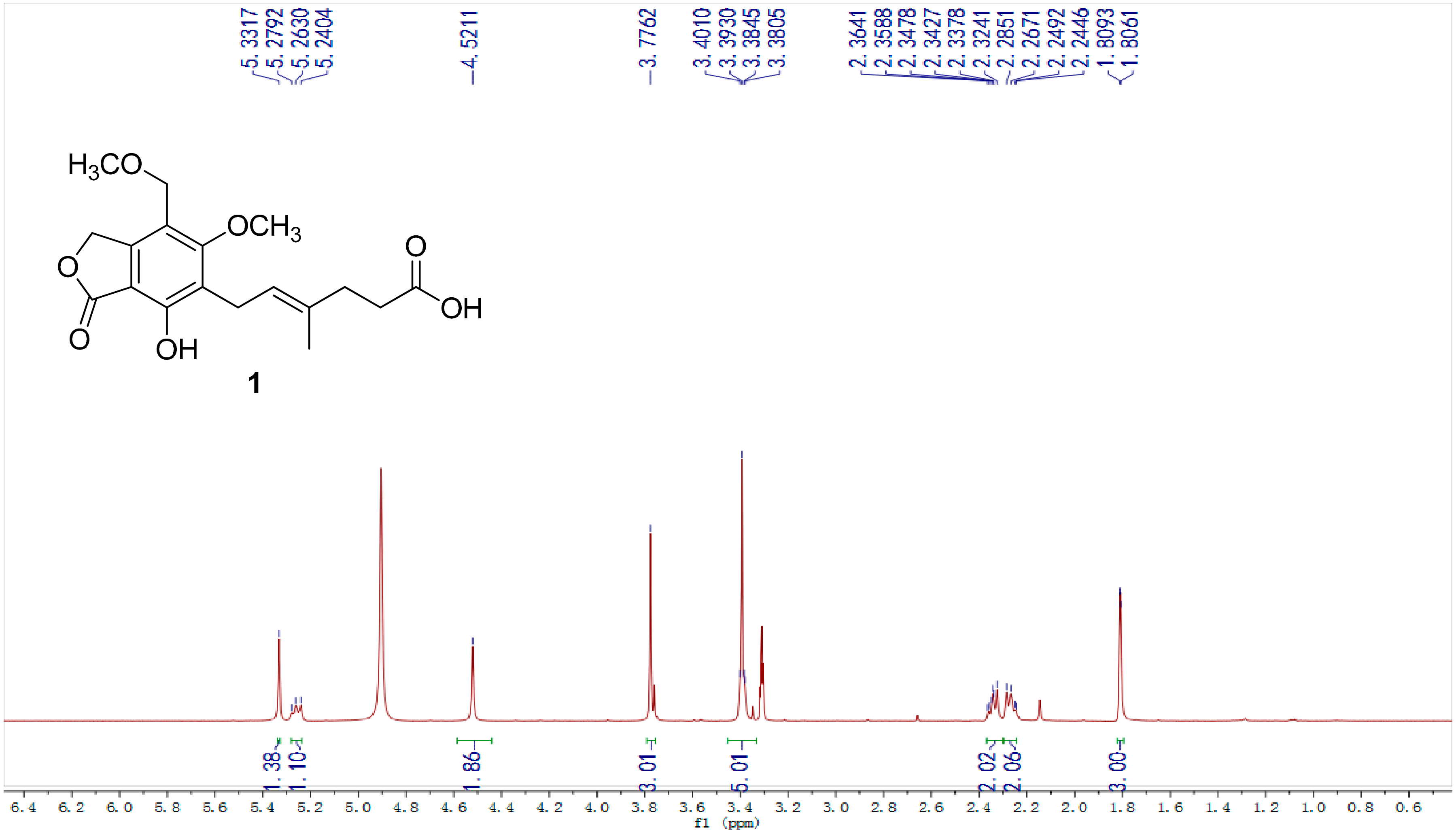

Figure S1. ^{1}H NMR spectrum of compound **1** (Recorded in CD_3OD)

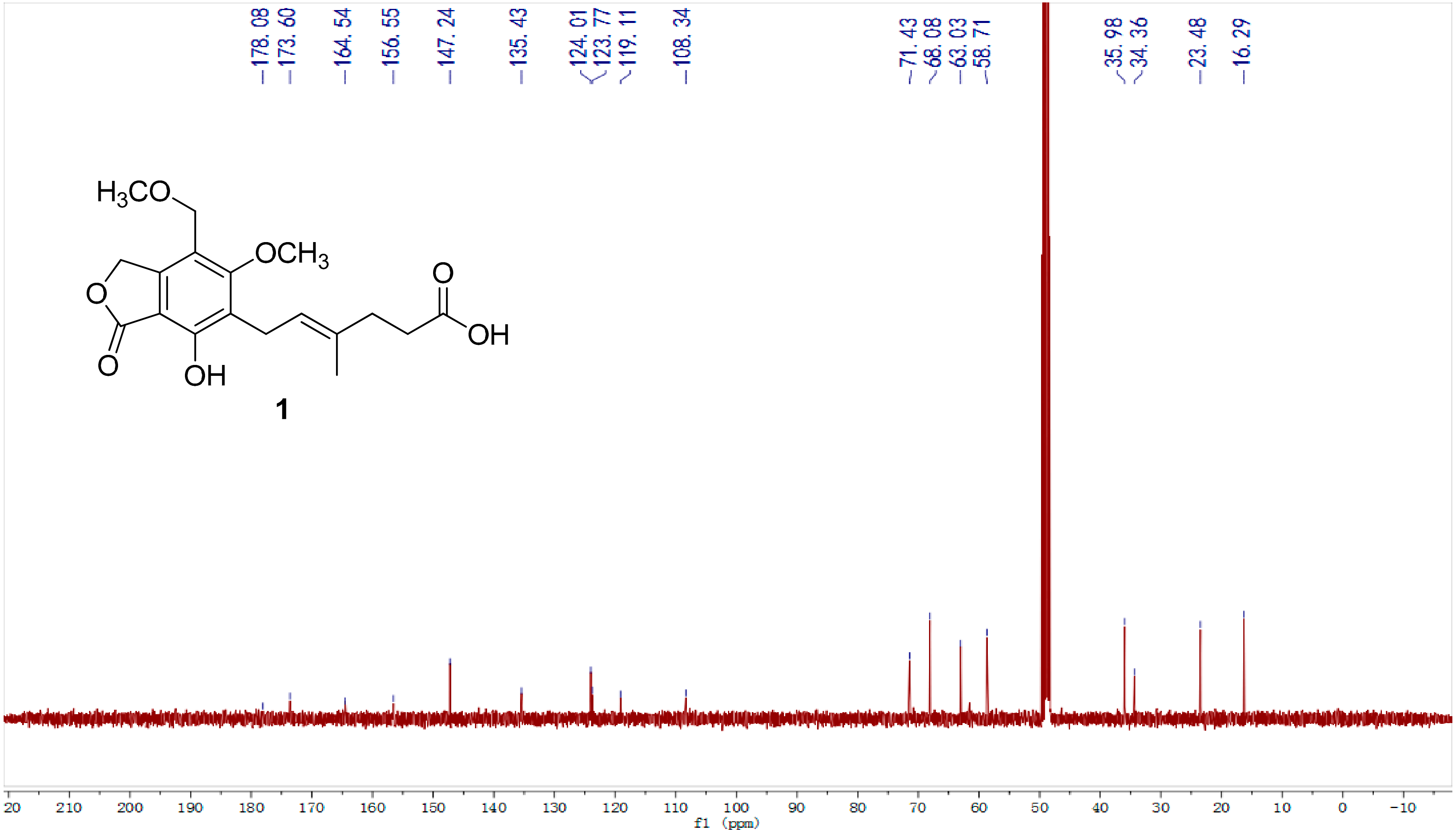

Figure S2. ^{13}C NMR spectrum of compound **1** (Recorded in CD_3OD)

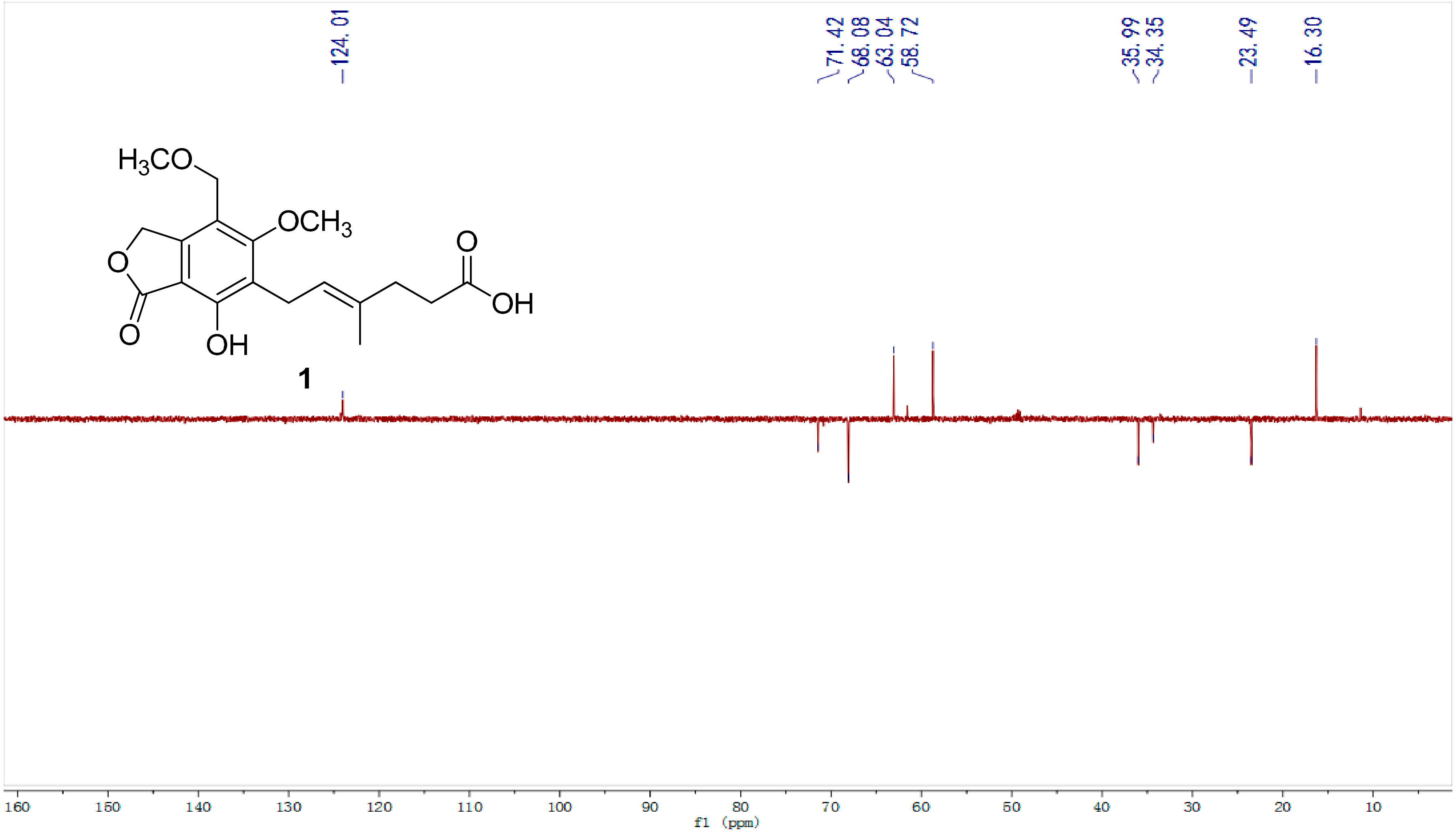

Figure S3. DEPT spectrum of compound **1** (Recorded in CD3OD)

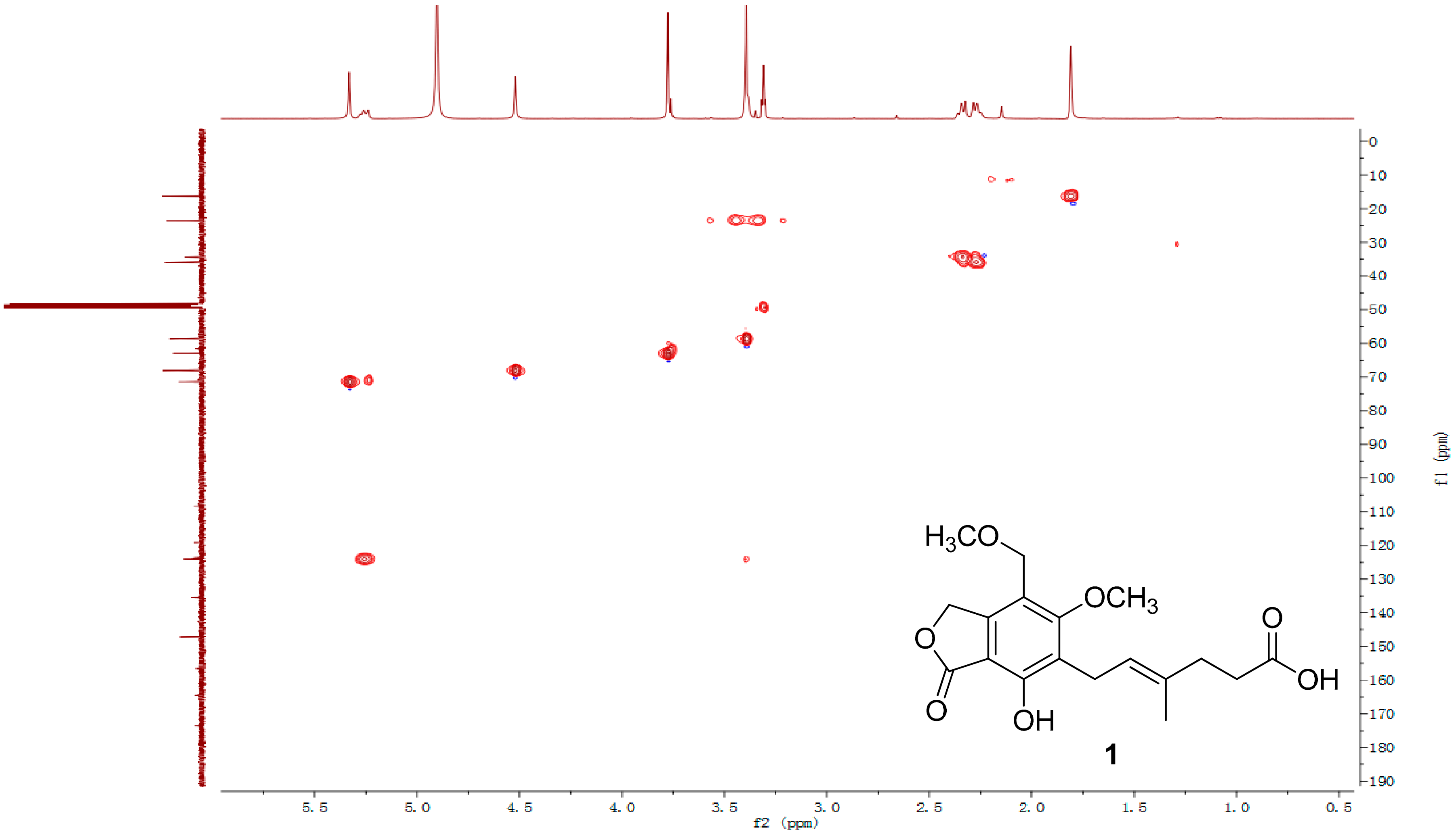

Figure S4. HSQC spectrum of compound **1** (Recorded in CD_3OD)

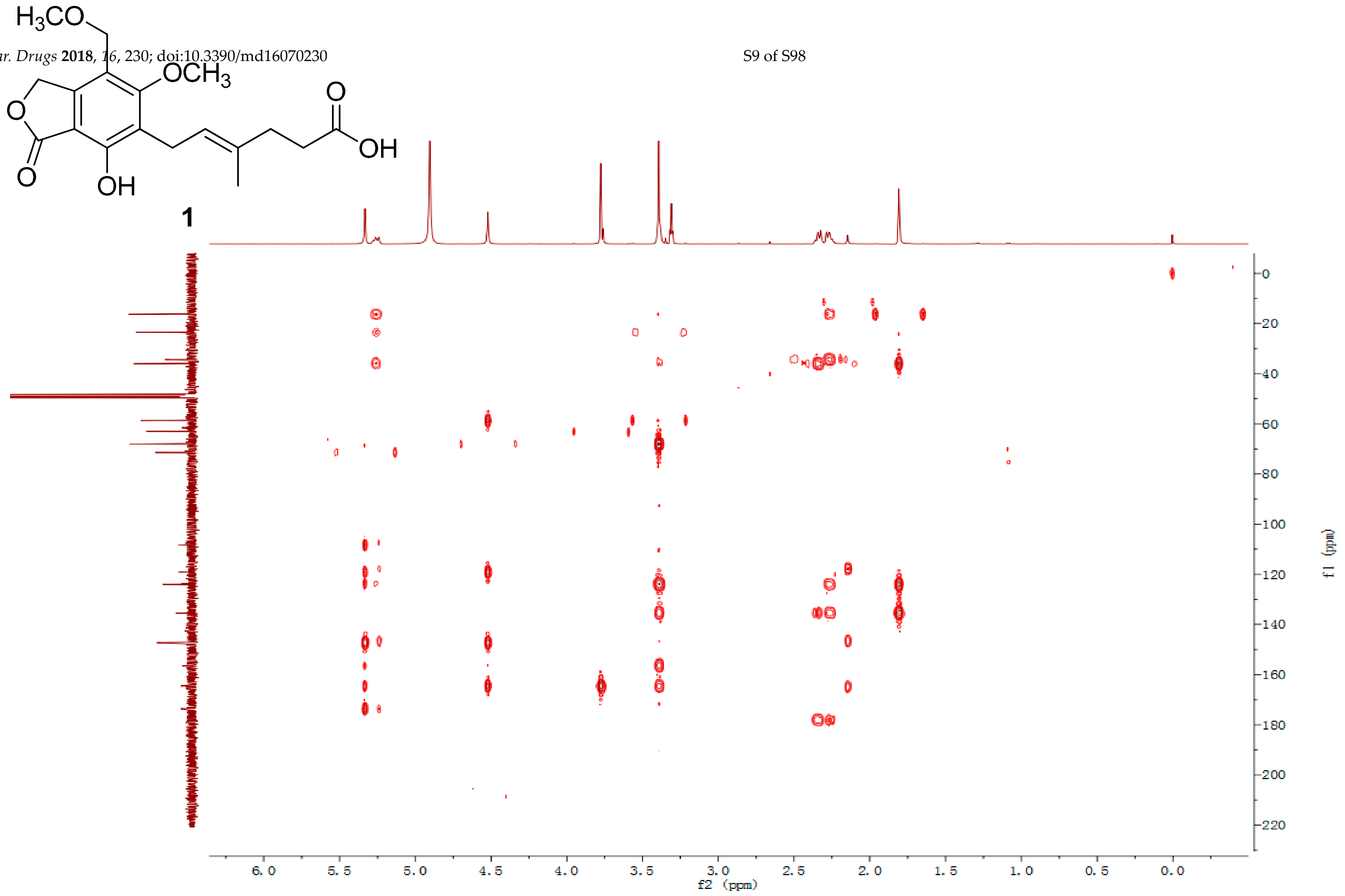

Figure S5. HMBC spectrum of compound **1** (Recorded in CD3OD)

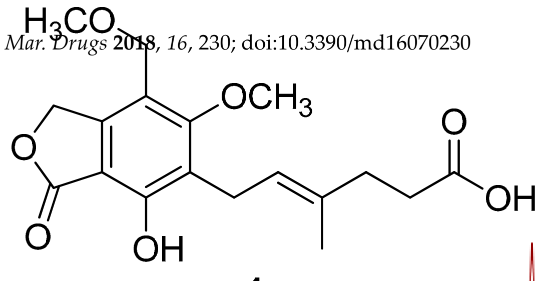

1

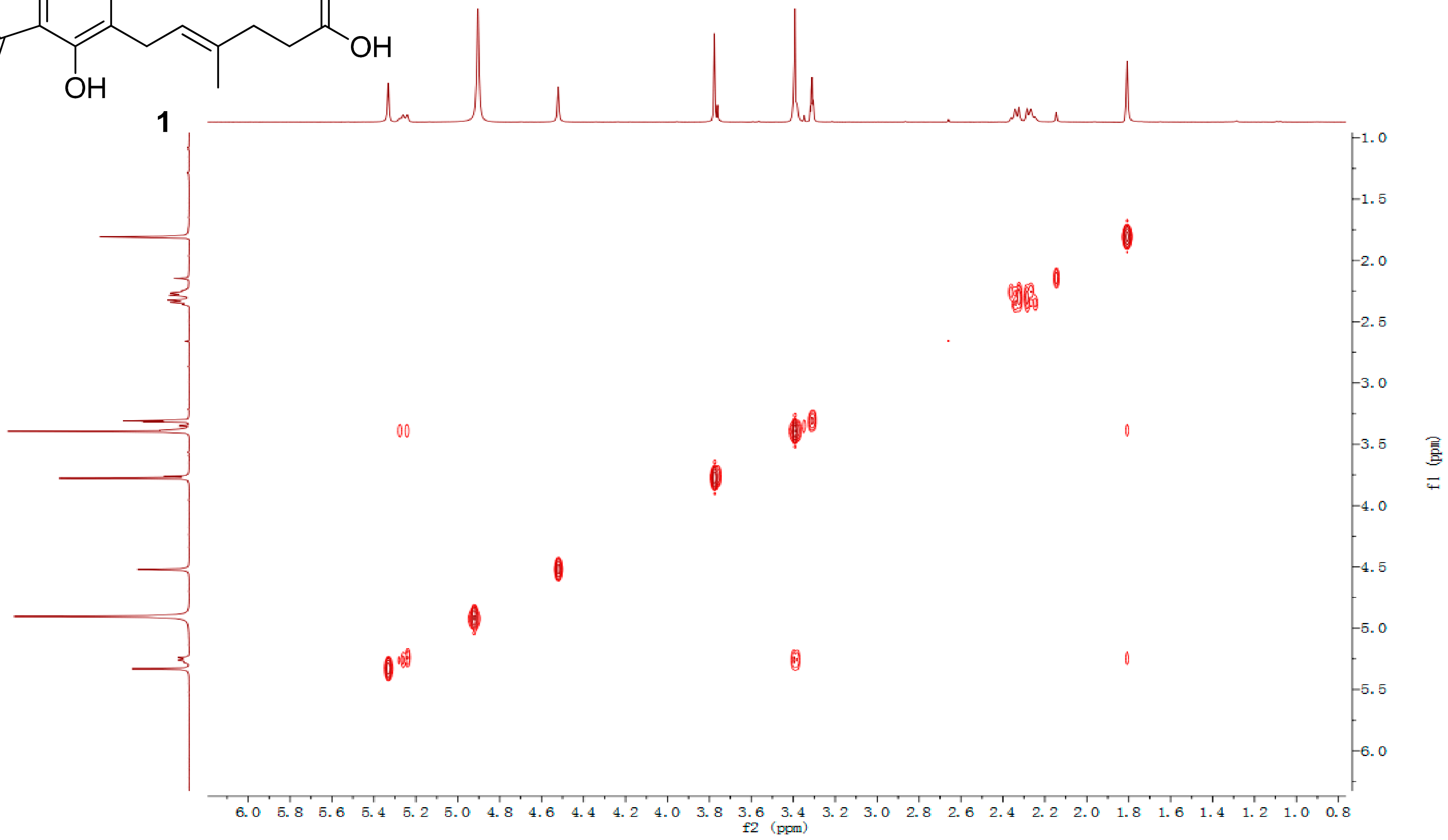

Figure S6. ^{1}H–^{1}H COSY spectrum of compound **1** (Recorded in CD_3OD)

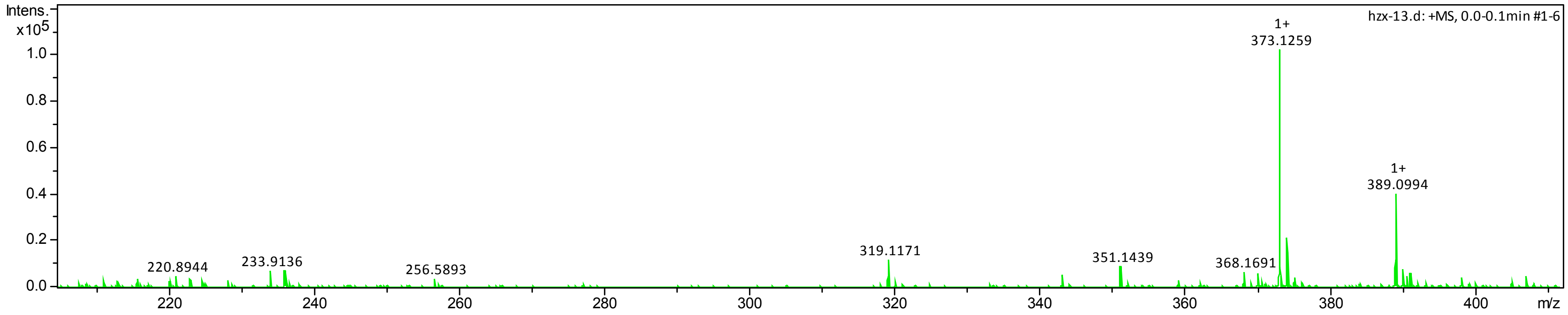

Figure S7. HRESIMS spectrum of compound **1**

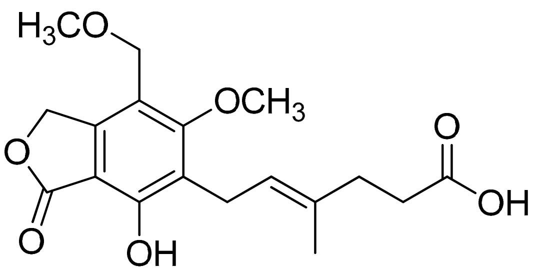

1

E:\20170419\yby\13.0

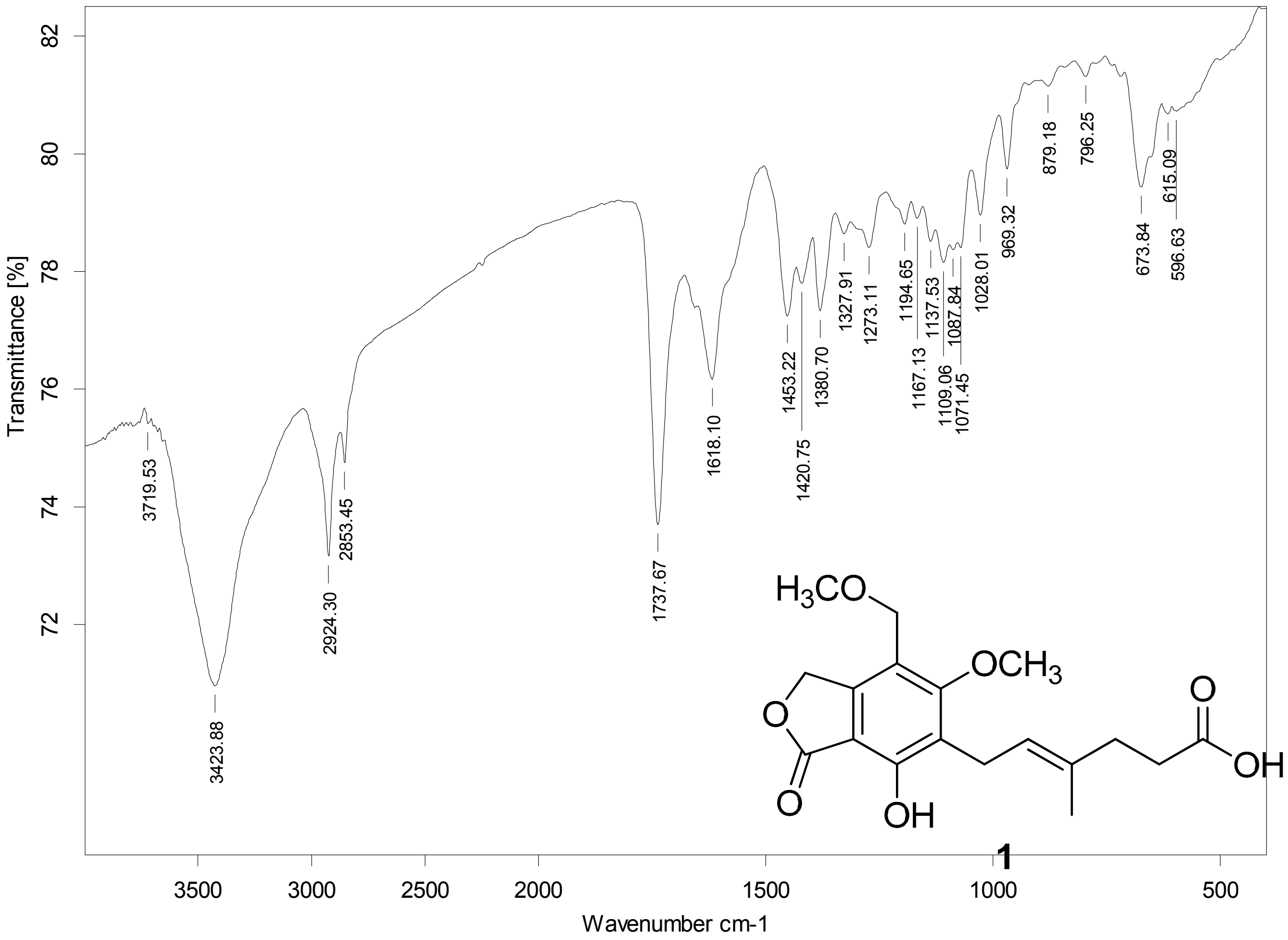

Figure S8. IR spectrum of compound **1**

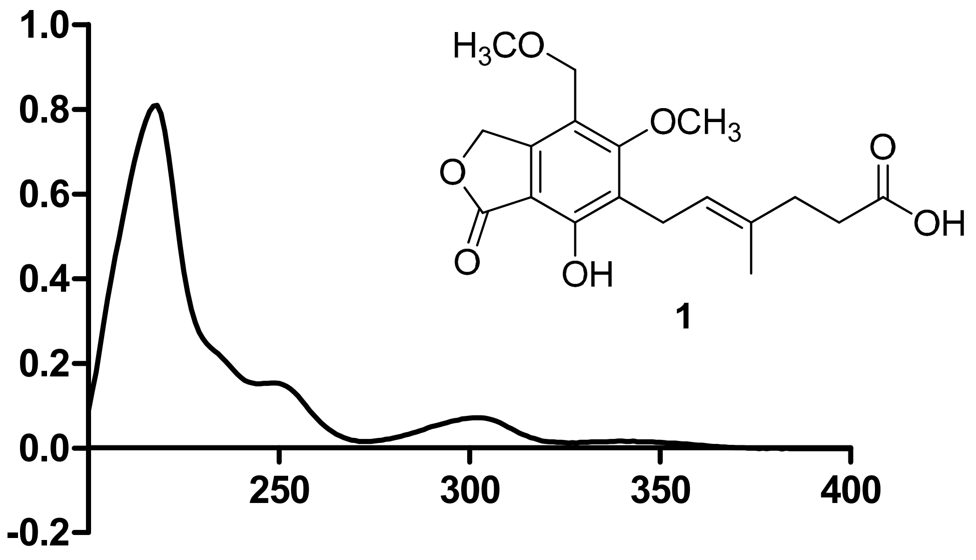

Figure S9. UV spectrum of compound **1**

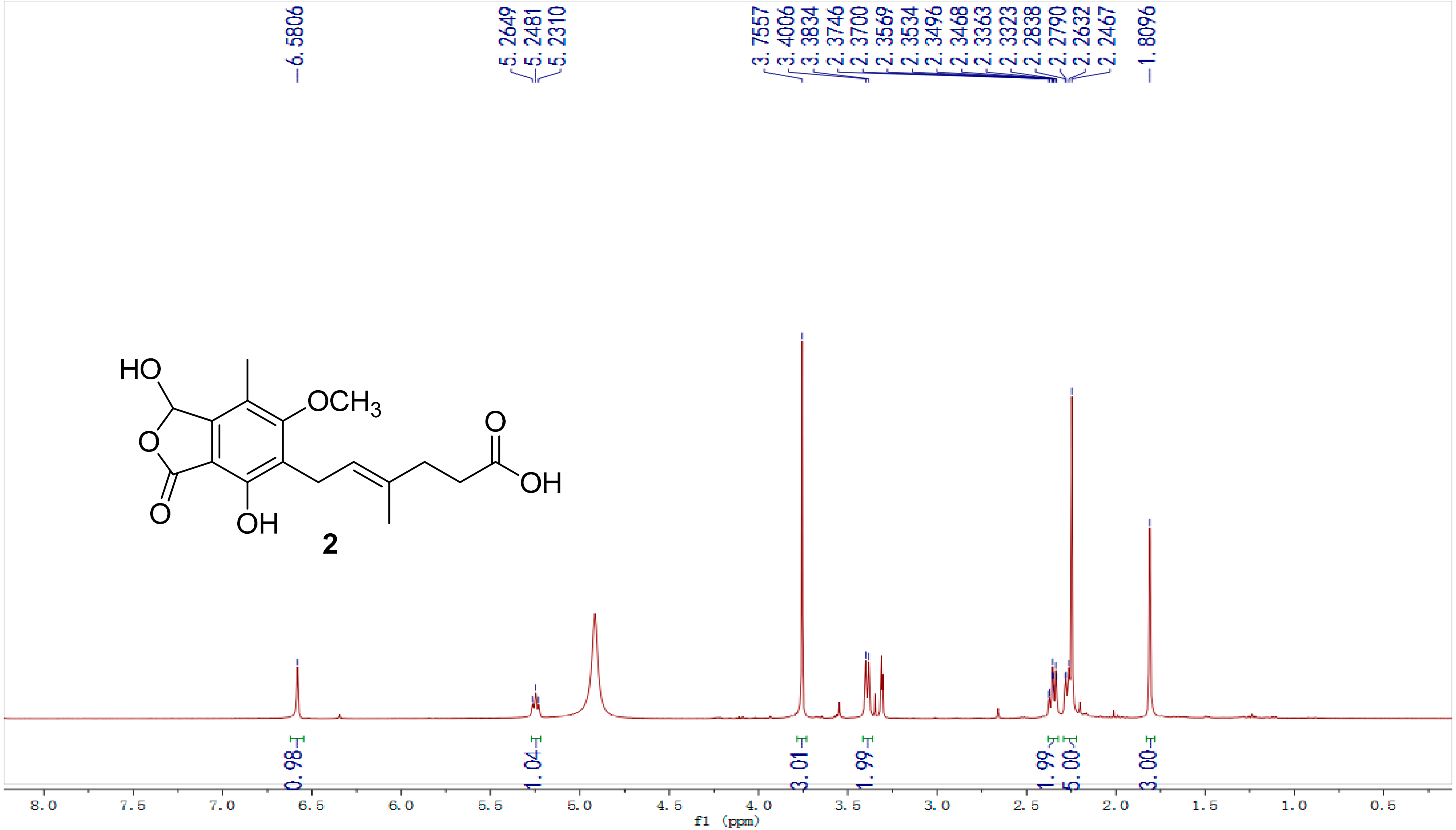

Figure S10. ^{1}H NMR spectrum of compound **2** (Recorded in CD_3OD)

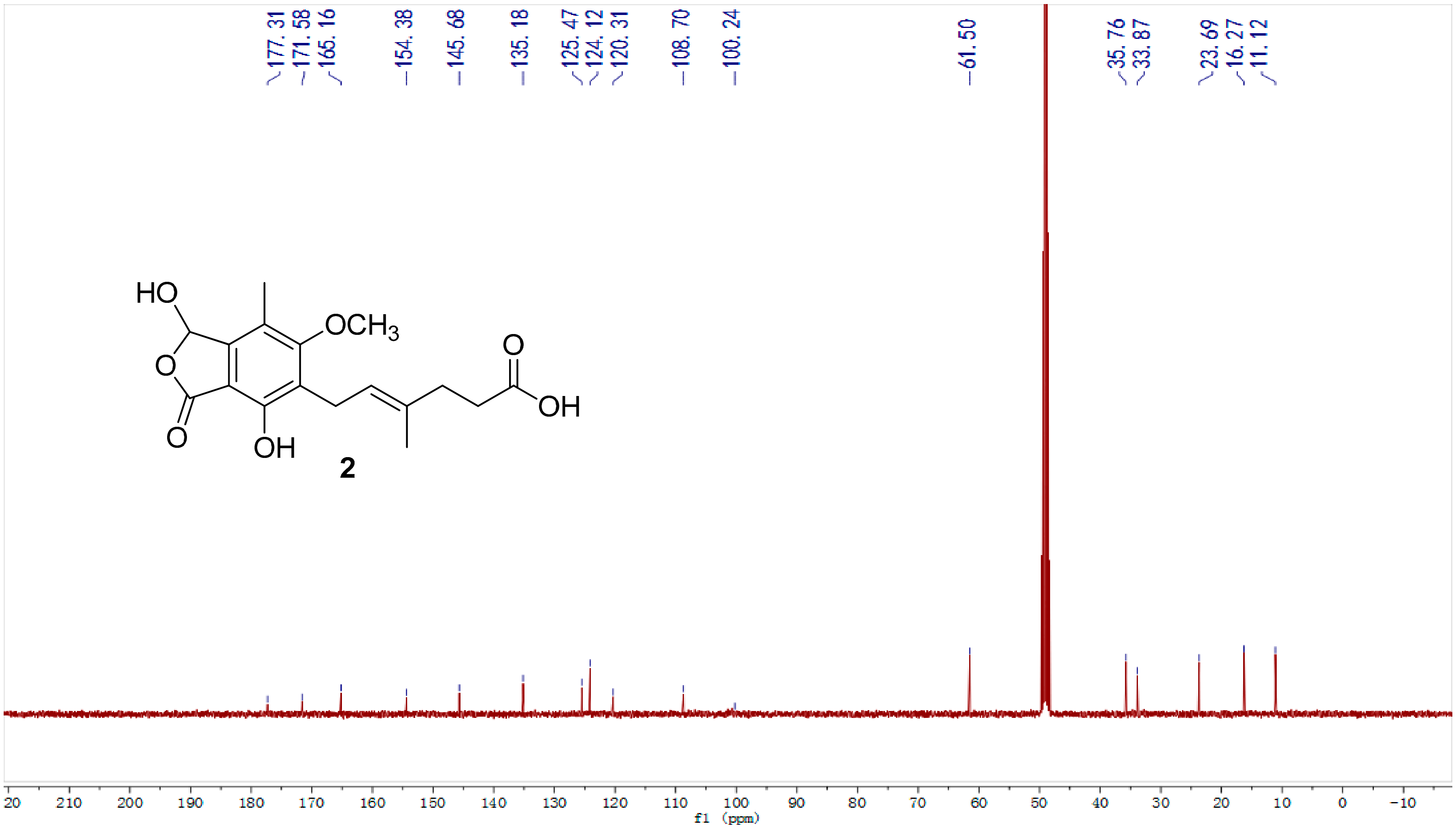

Figure S11. ^{13}C NMR spectrum of compound **2** (Recorded in CD_3OD)

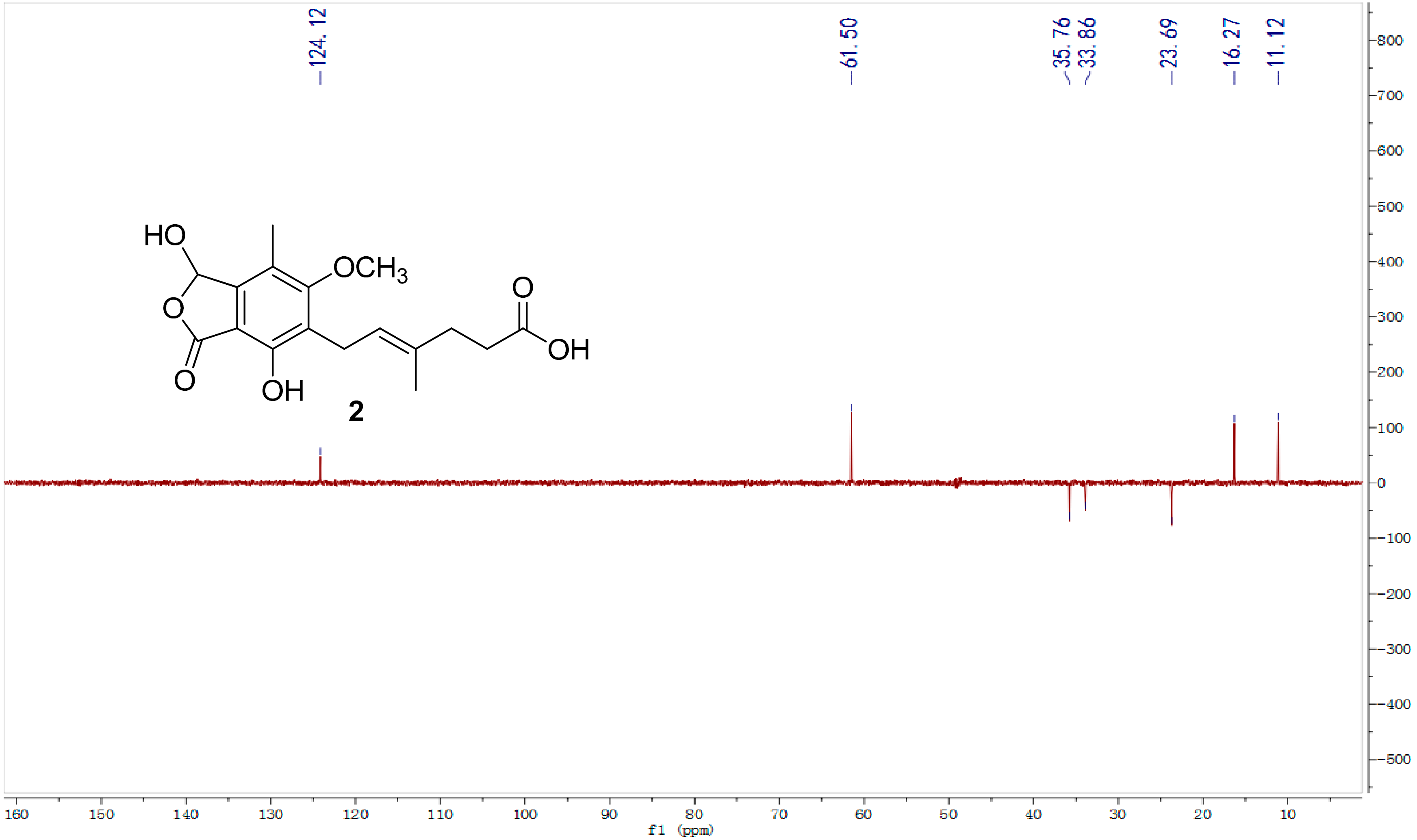

Figure S12. DEPT spectrum of compound **2** (Recorded in CD_3OD)

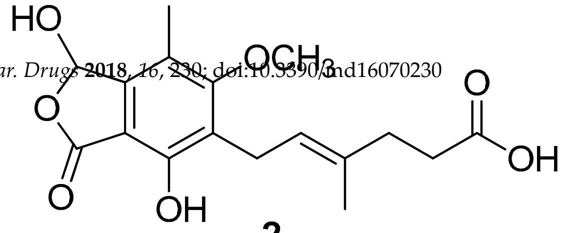

2

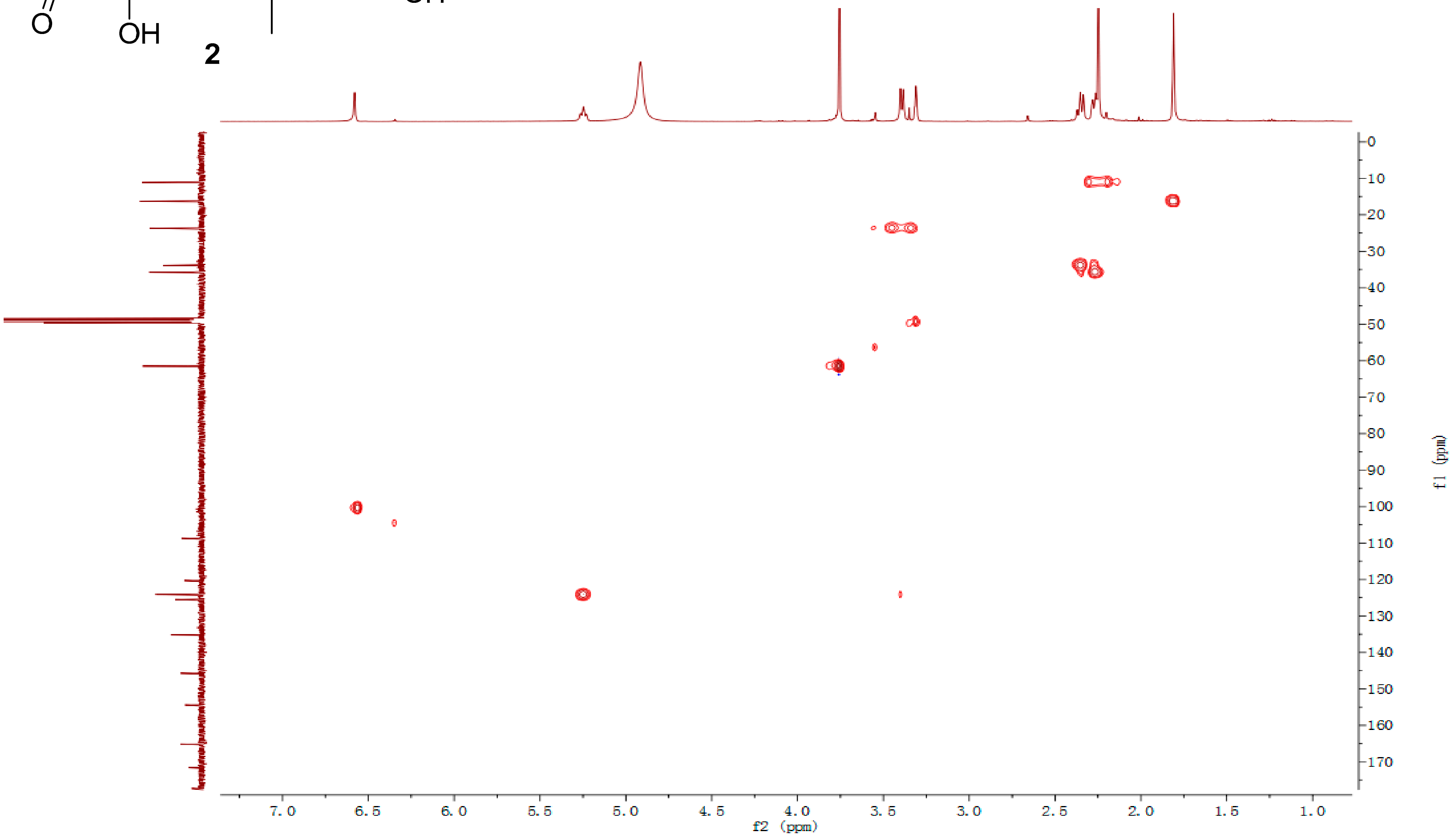

Figure S13. HSQC spectrum of compound **2** (Recorded in CD_3OD)

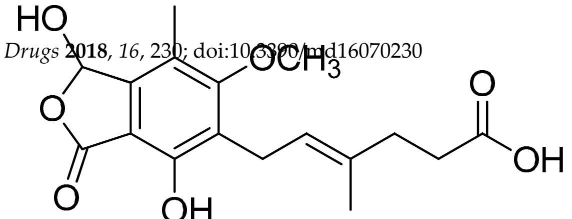

2

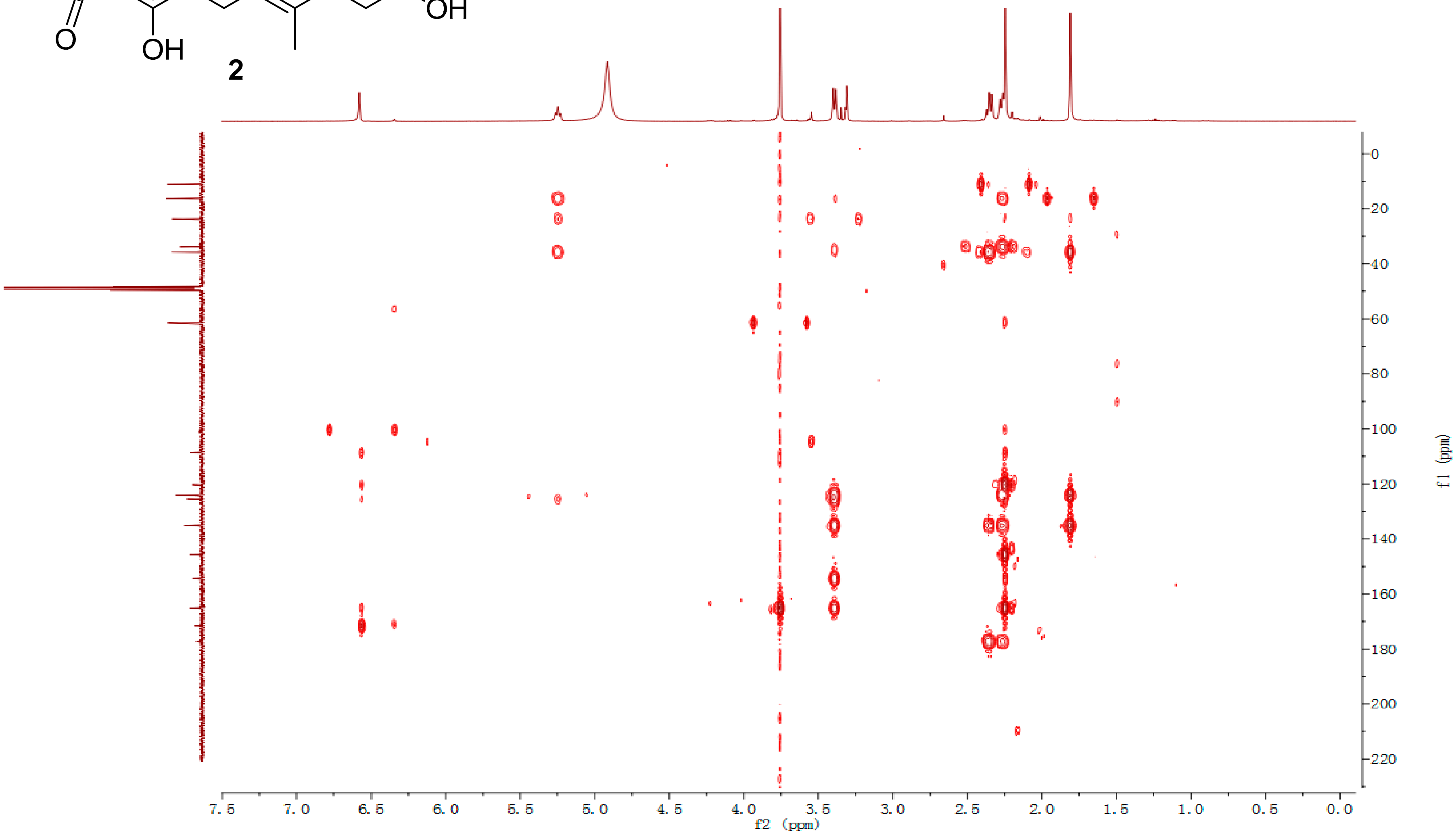

Figure S14. HMBC spectrum of compound **2** (Recorded in CD3OD)

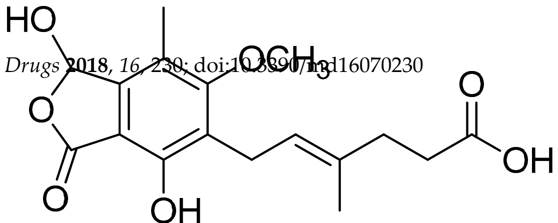

2

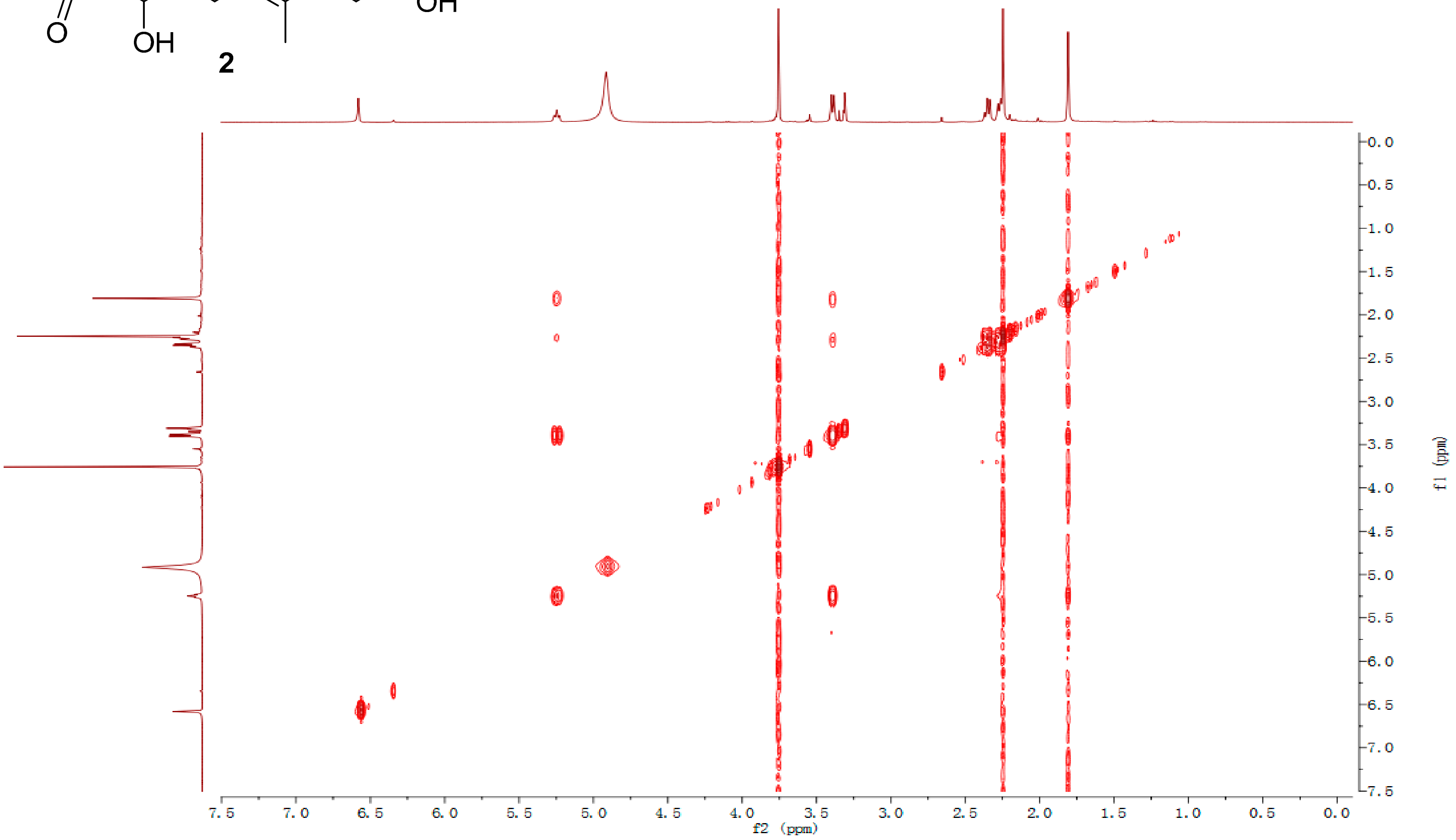

Figure S15. ^{1}H–^{1}H COSY spectrum of compound **2** (Recorded in CD_3OD)

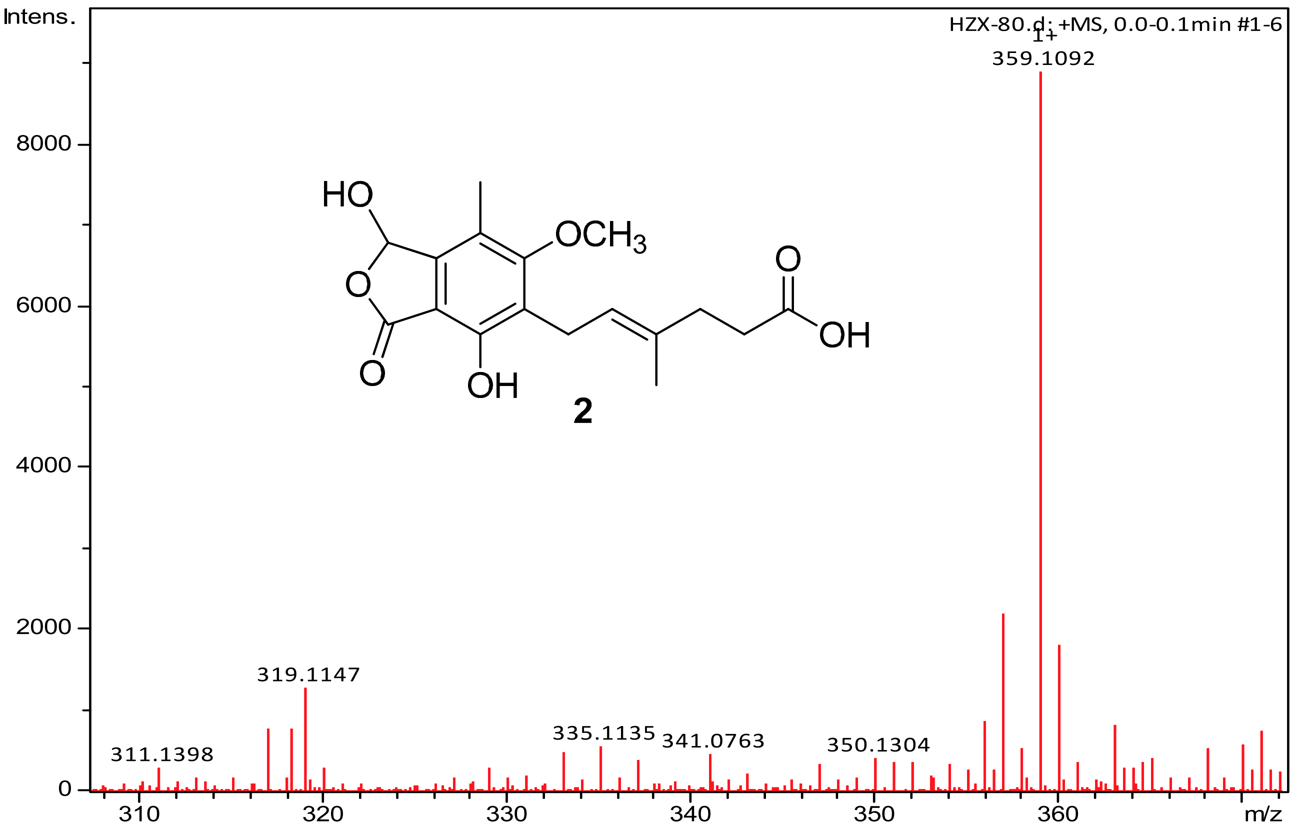

Figure S16. HRESIMS spectrum of compound **2**

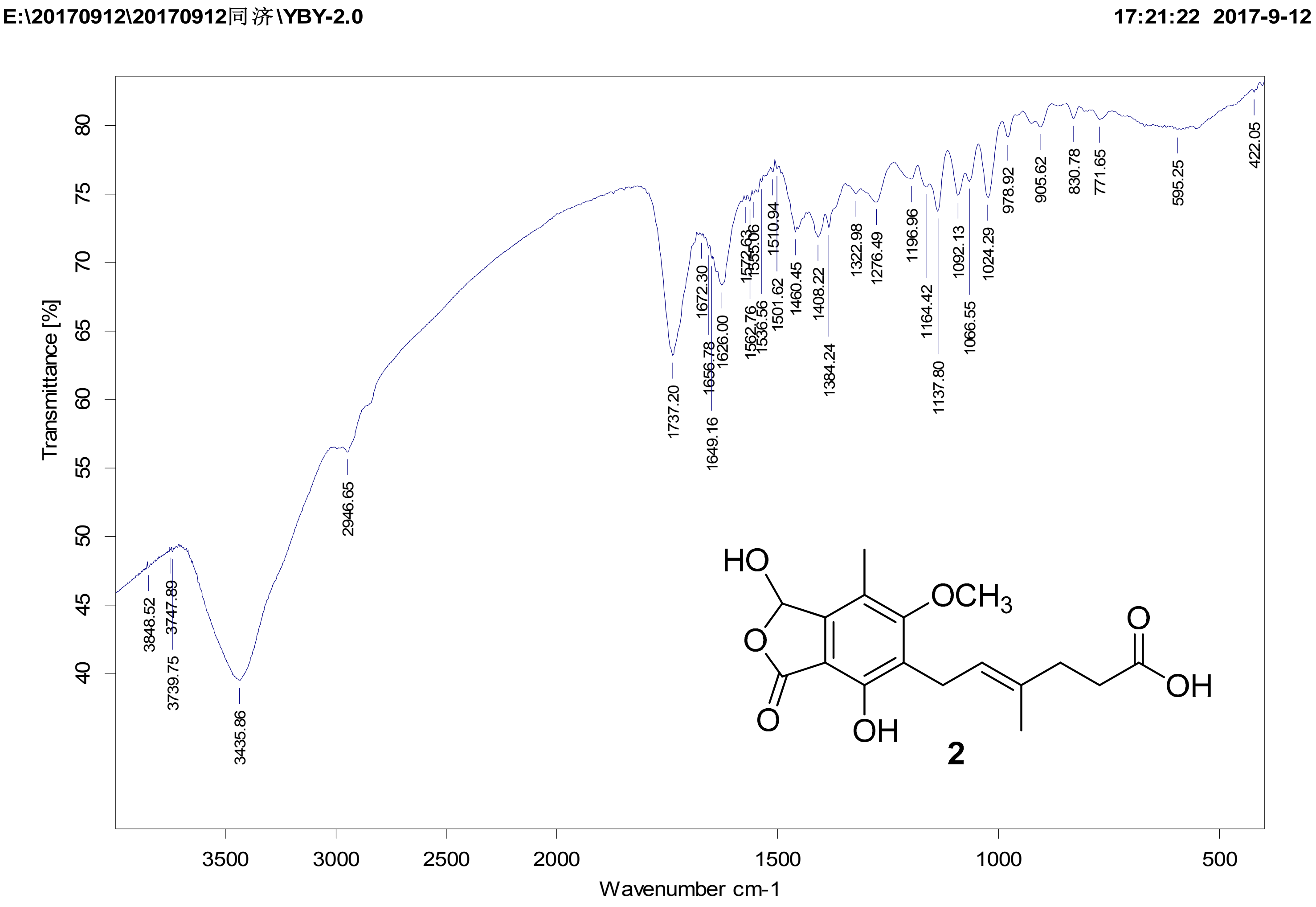

Figure S17. IR spectrum of compound **2**

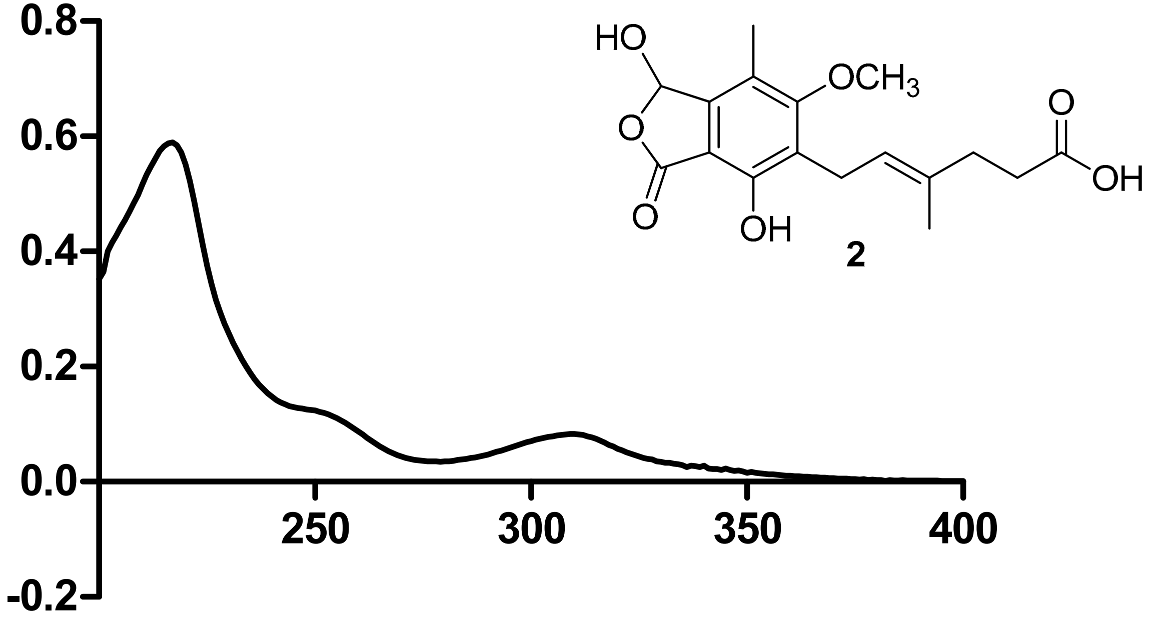

Figure S18. UV spectrum of compound **2**

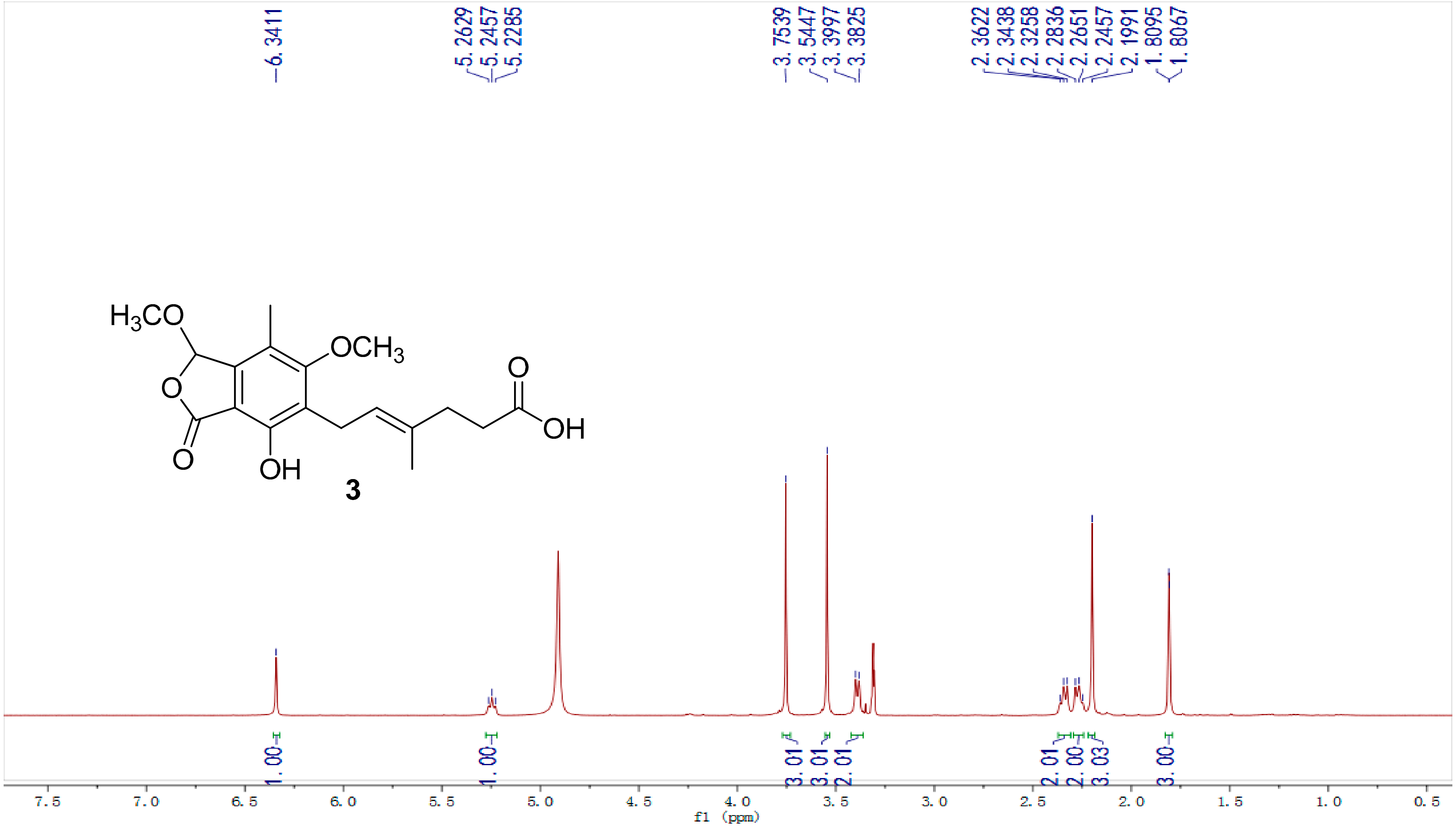

Figure S19. ^{1}H NMR spectrum of compound **3** (Recorded in CD_3OD)

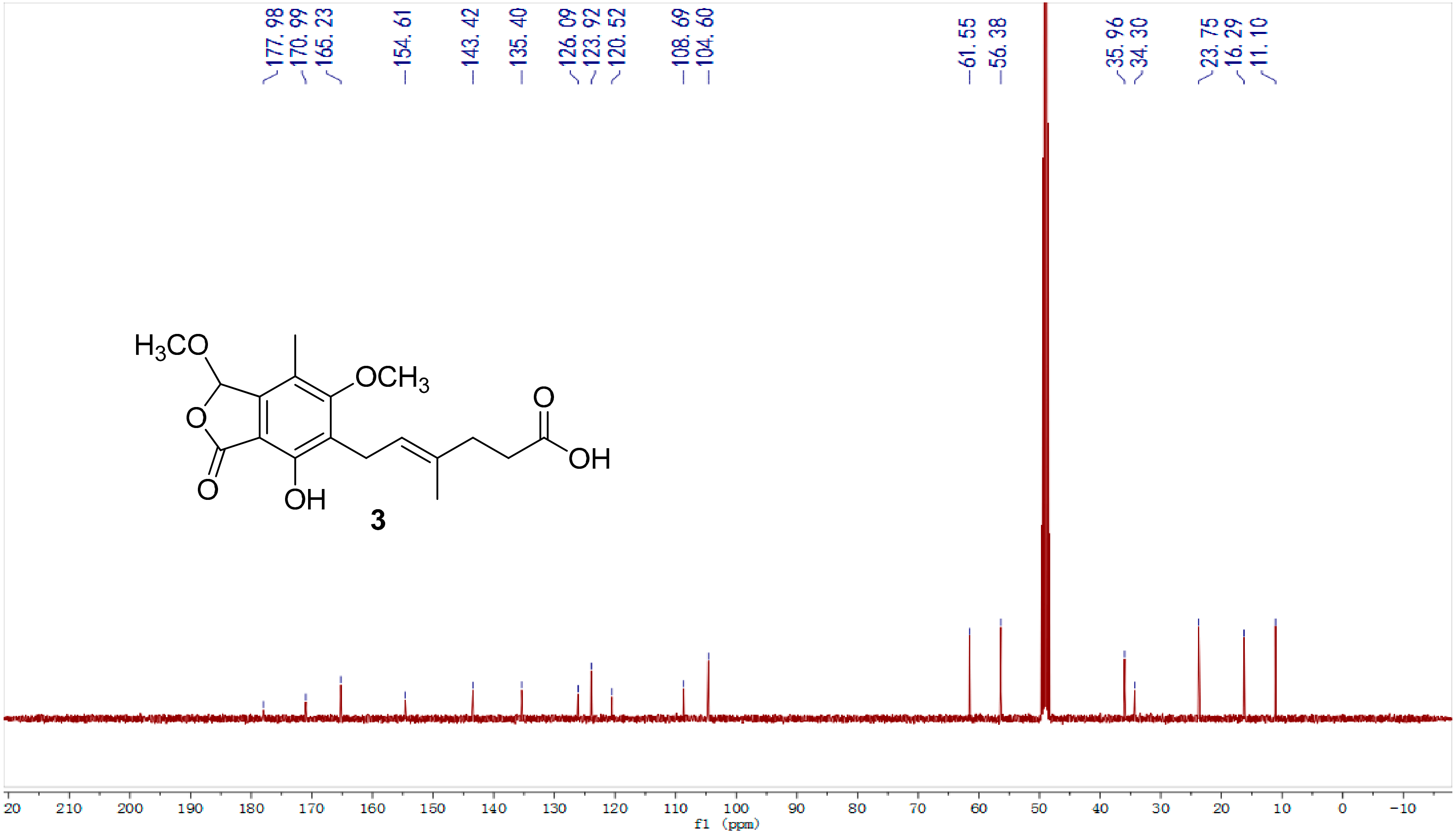

Figure S20. ^{13}C NMR spectrum of compound **3** (Recorded in CD_3OD)

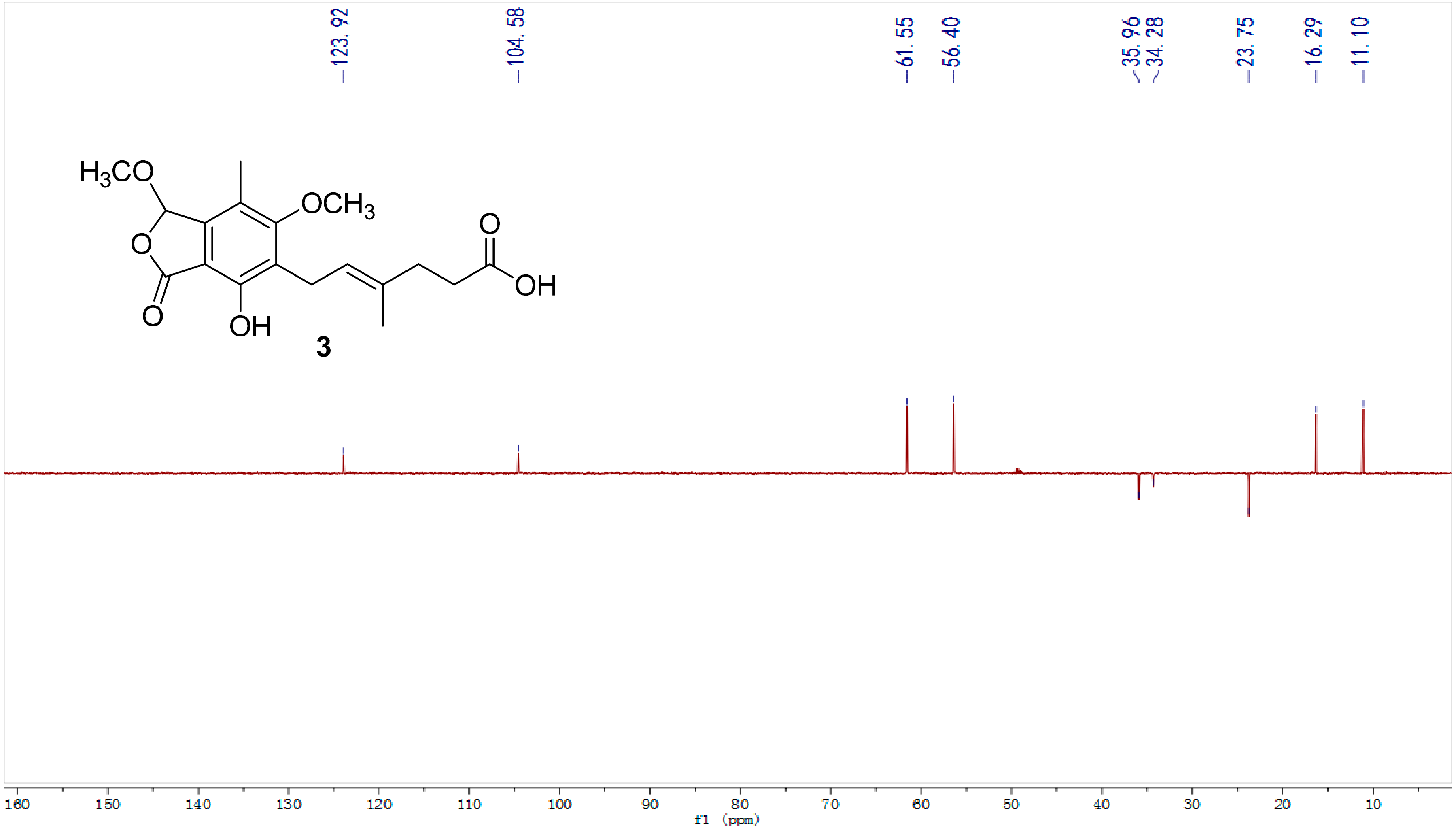

Figure S21. DEPT spectrum of compound **3** (Recorded in CD_3OD)

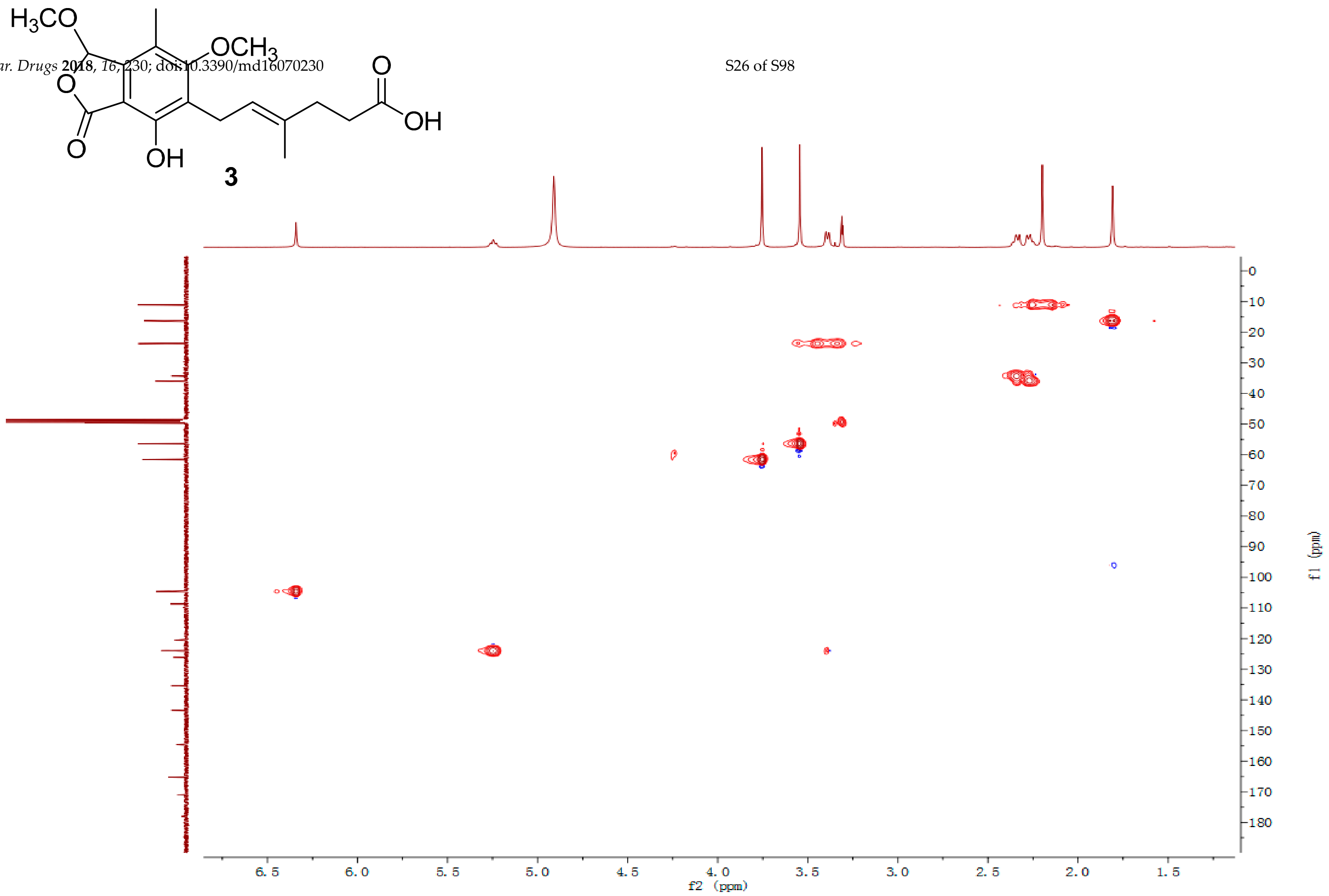

Figure S22. HSQC spectrum of compound **3** (Recorded in CD_3OD)

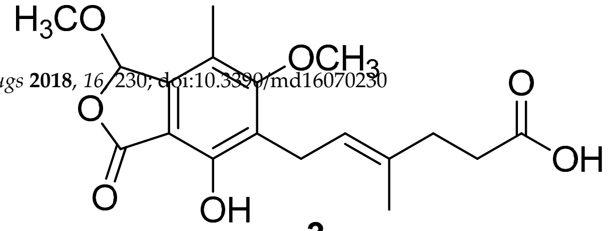

3

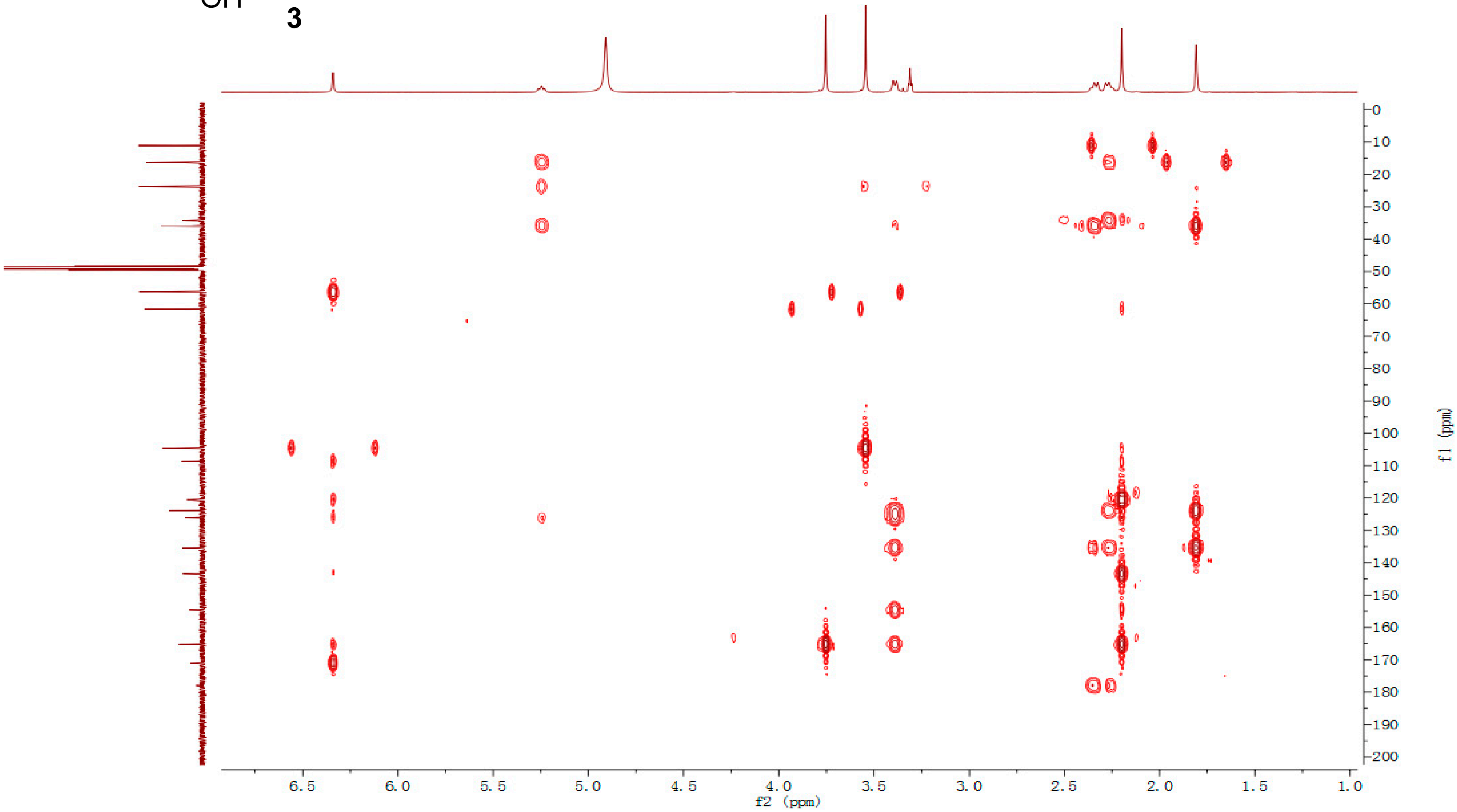

Figure S23. HMBC spectrum of compound **3** (Recorded in CD3OD)

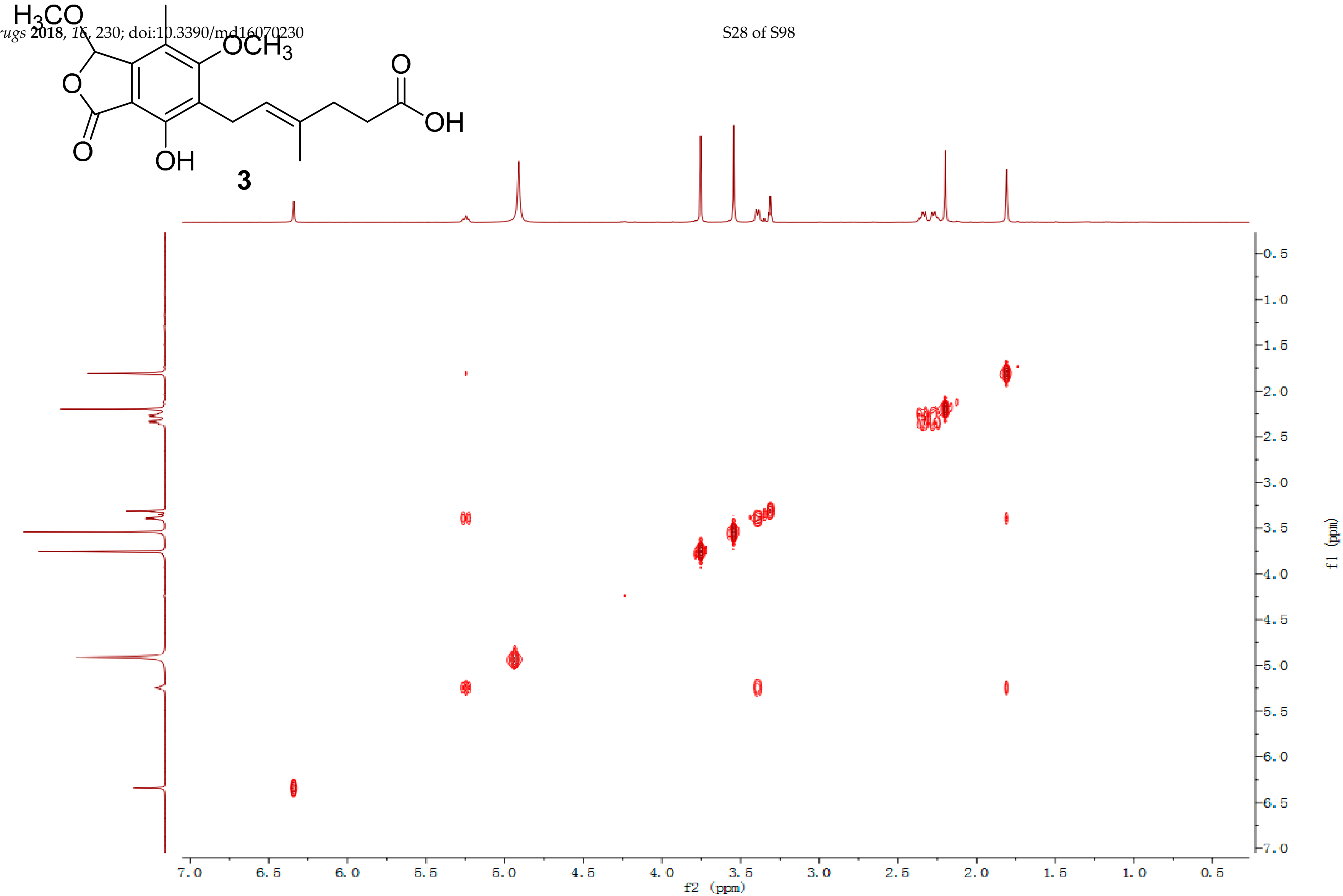

Figure S24. ^{1}H–^{1}H COSY spectrum of compound **3** (Recorded in CD_3OD)

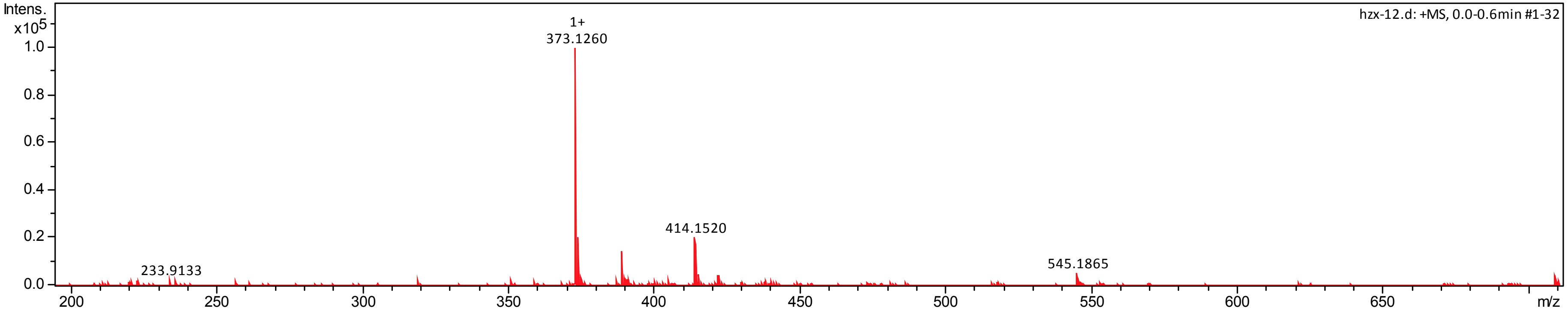

Figure S25. HRESIMS spectrum of compound **3**

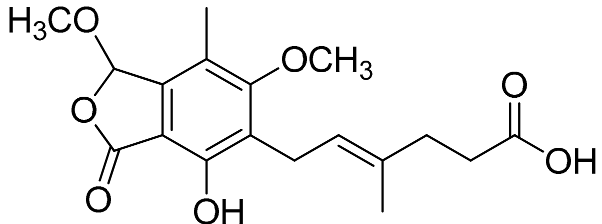

3

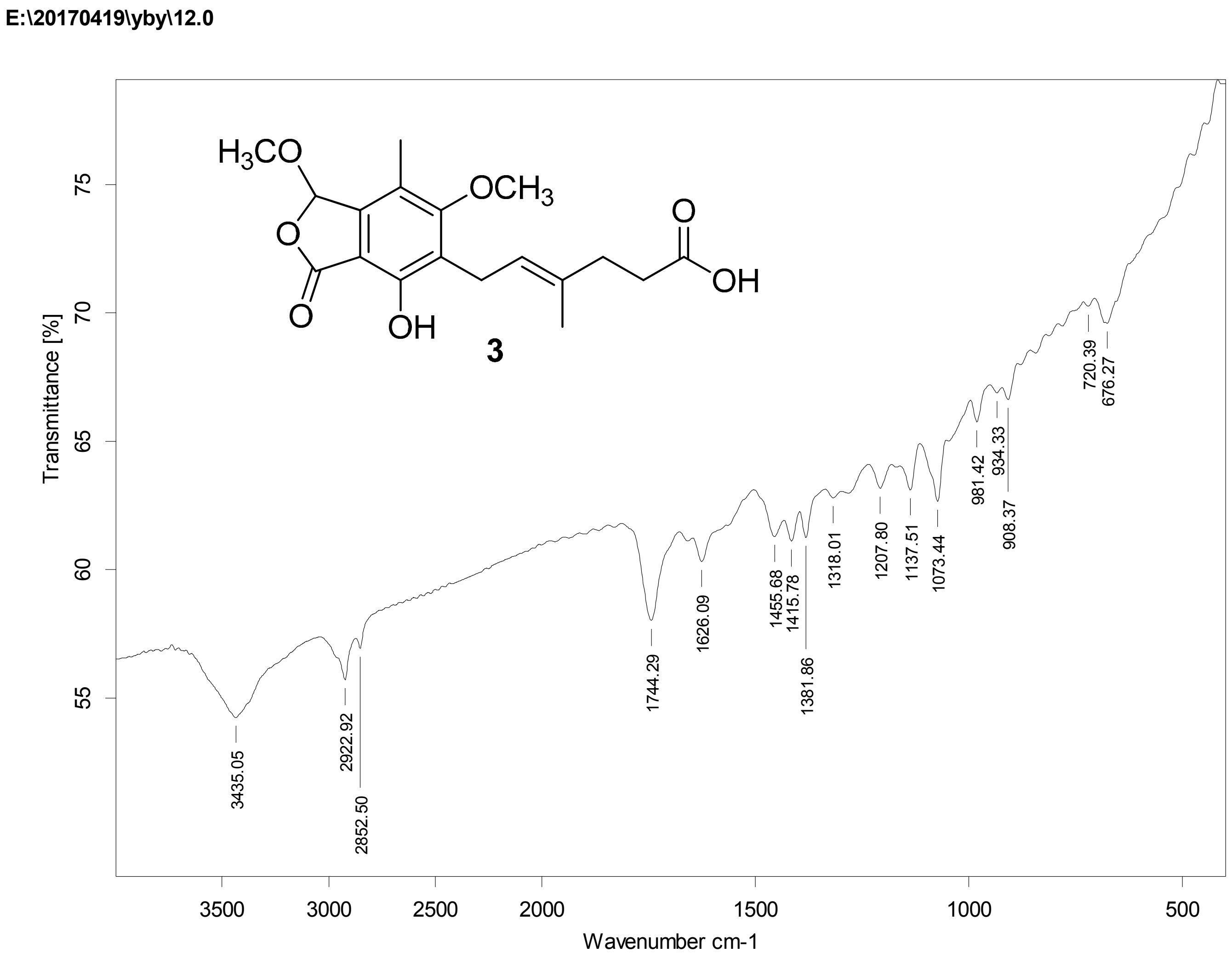

Figure S26. IR spectrum of compound **3**

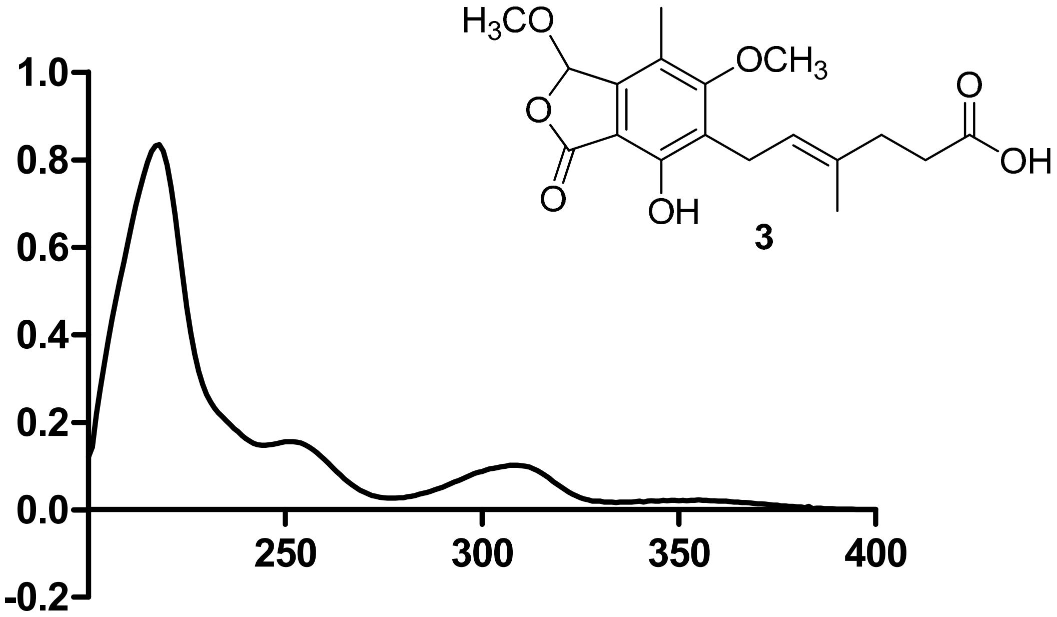

Figure S27. UV spectrum of compound **3**

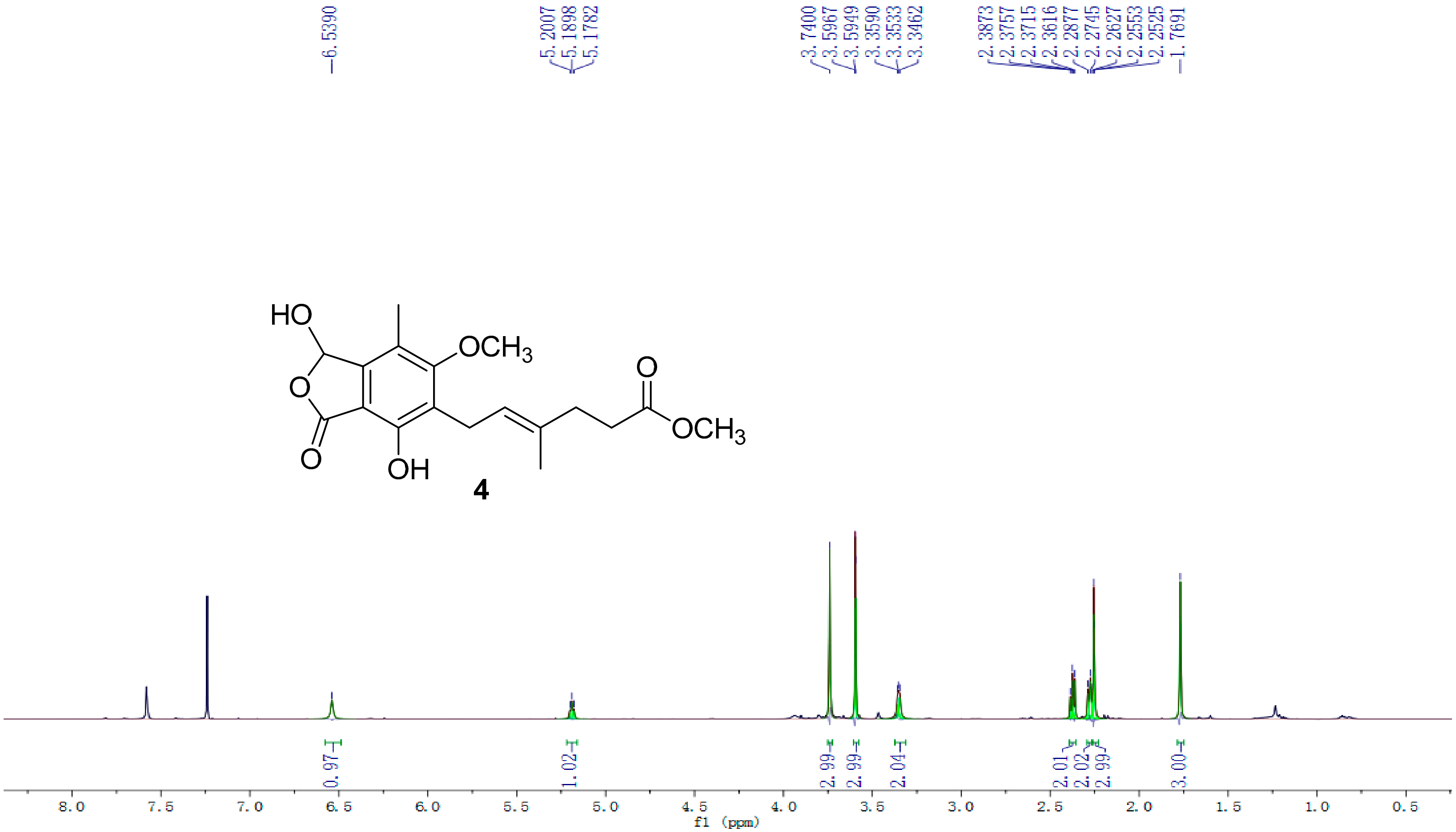

Figure S28. ^{1}H NMR spectrum of compound **4** (Recorded in CD_3OD)

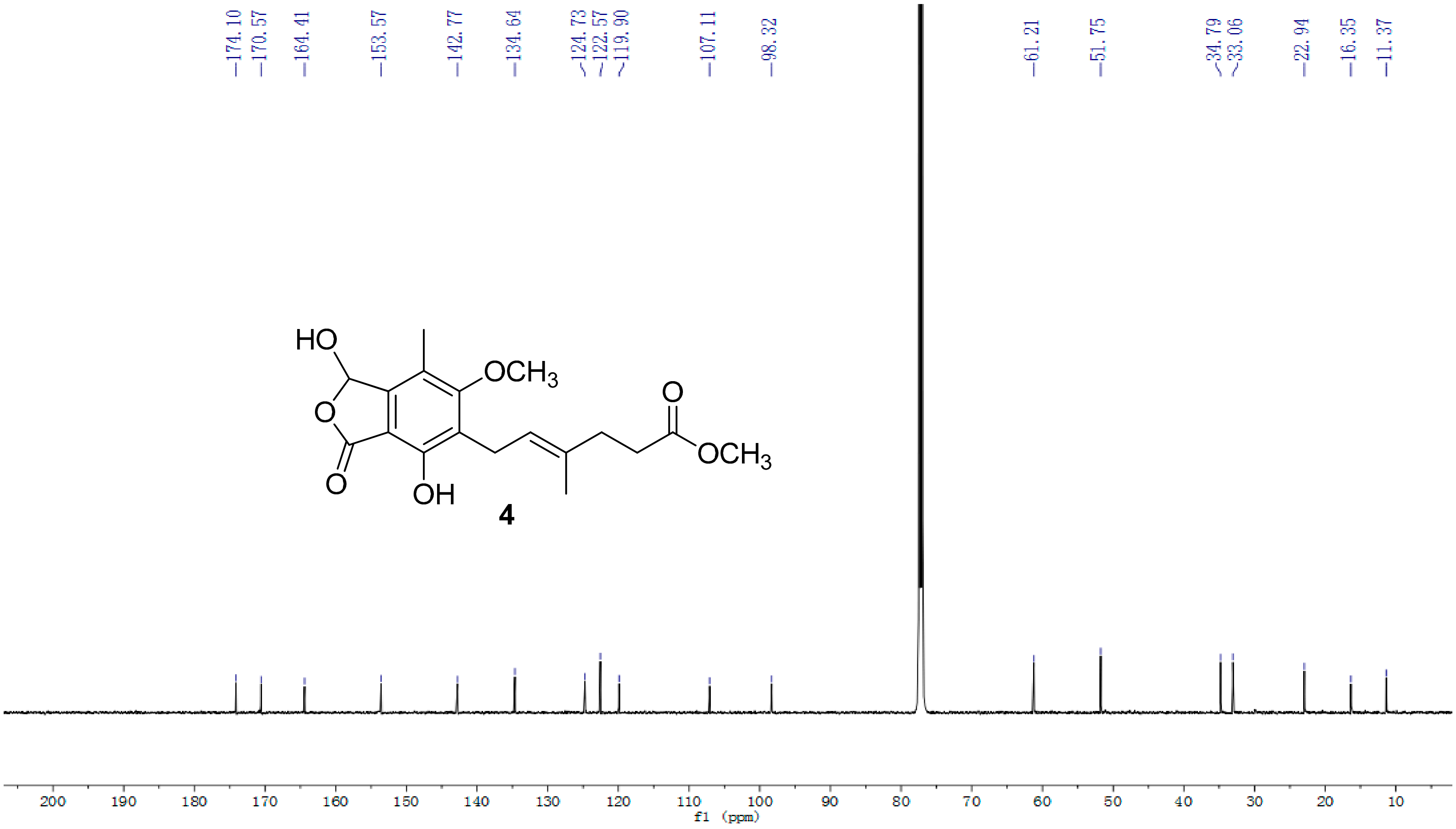

Figure S29. ^{13}C NMR spectrum of compound **4** (Recorded in CD_3OD)

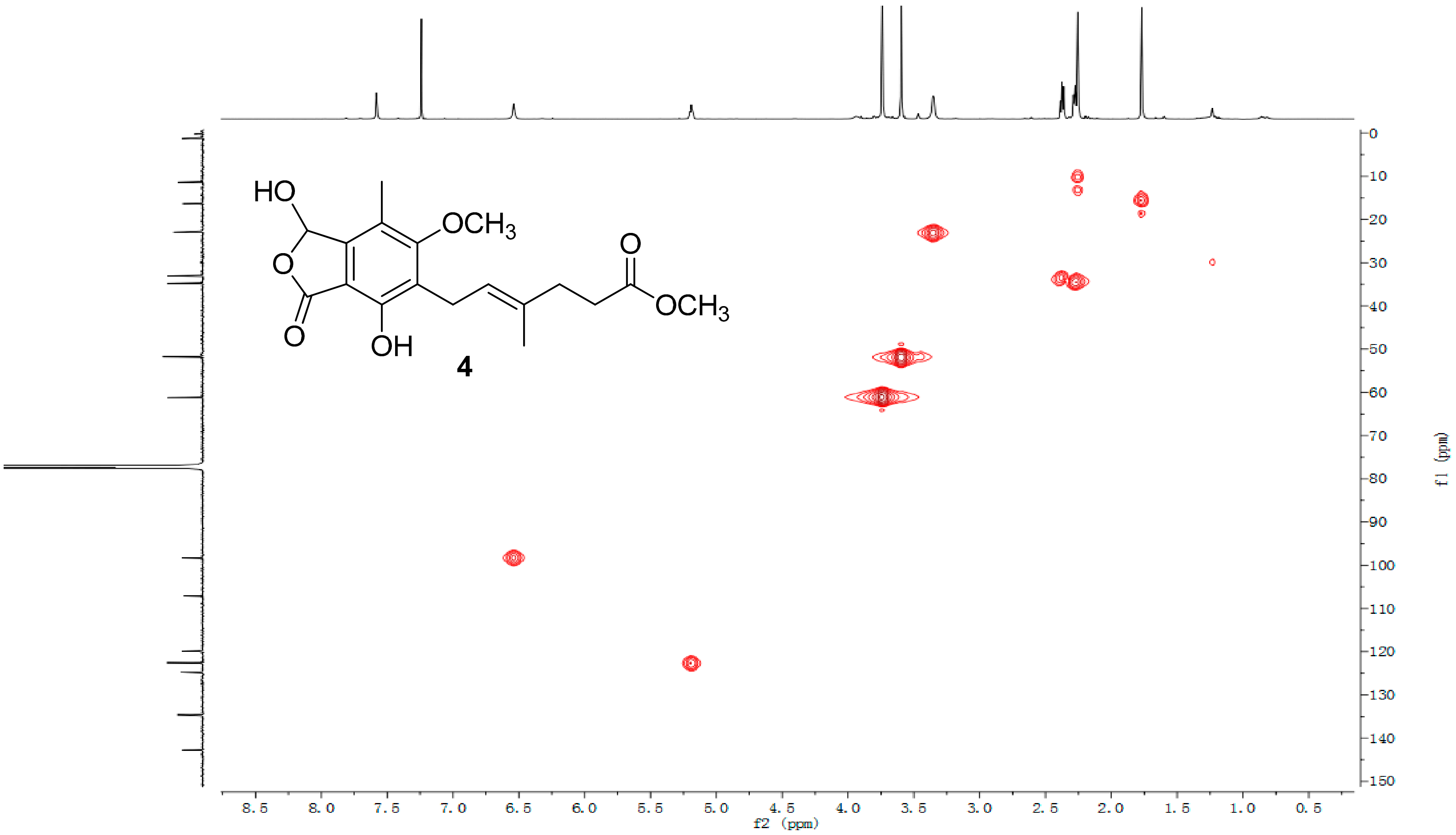

Figure S30. HSQC spectrum of compound **4** (Recorded in CD_3OD)

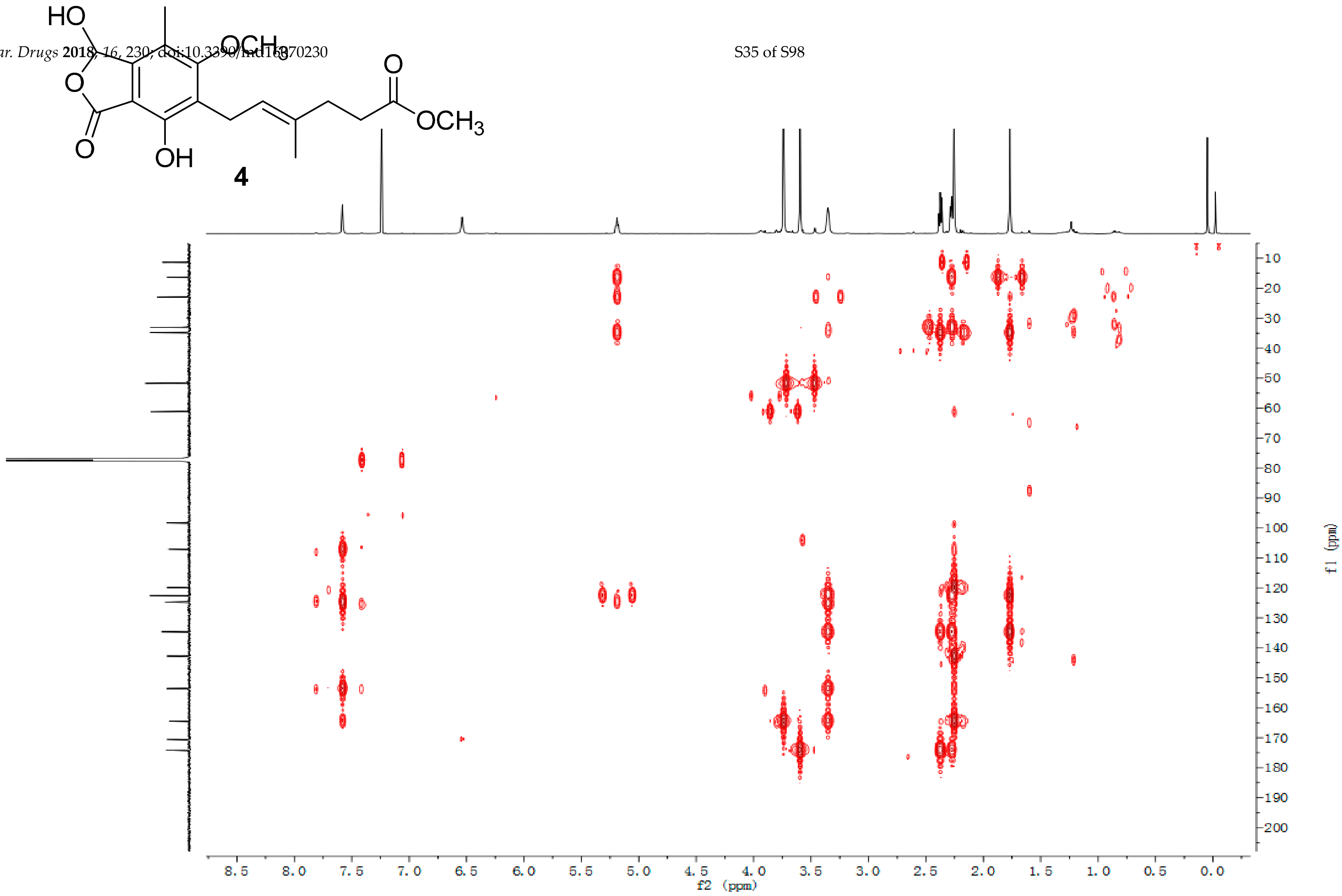

Figure S31. HMBC spectrum of compound **4** (Recorded in CD3OD)

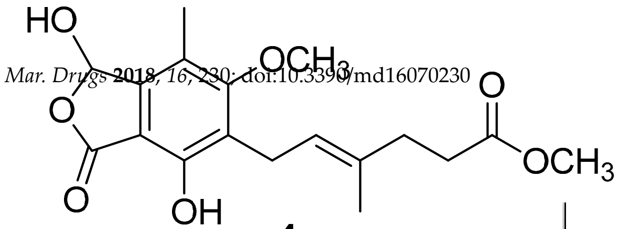

4

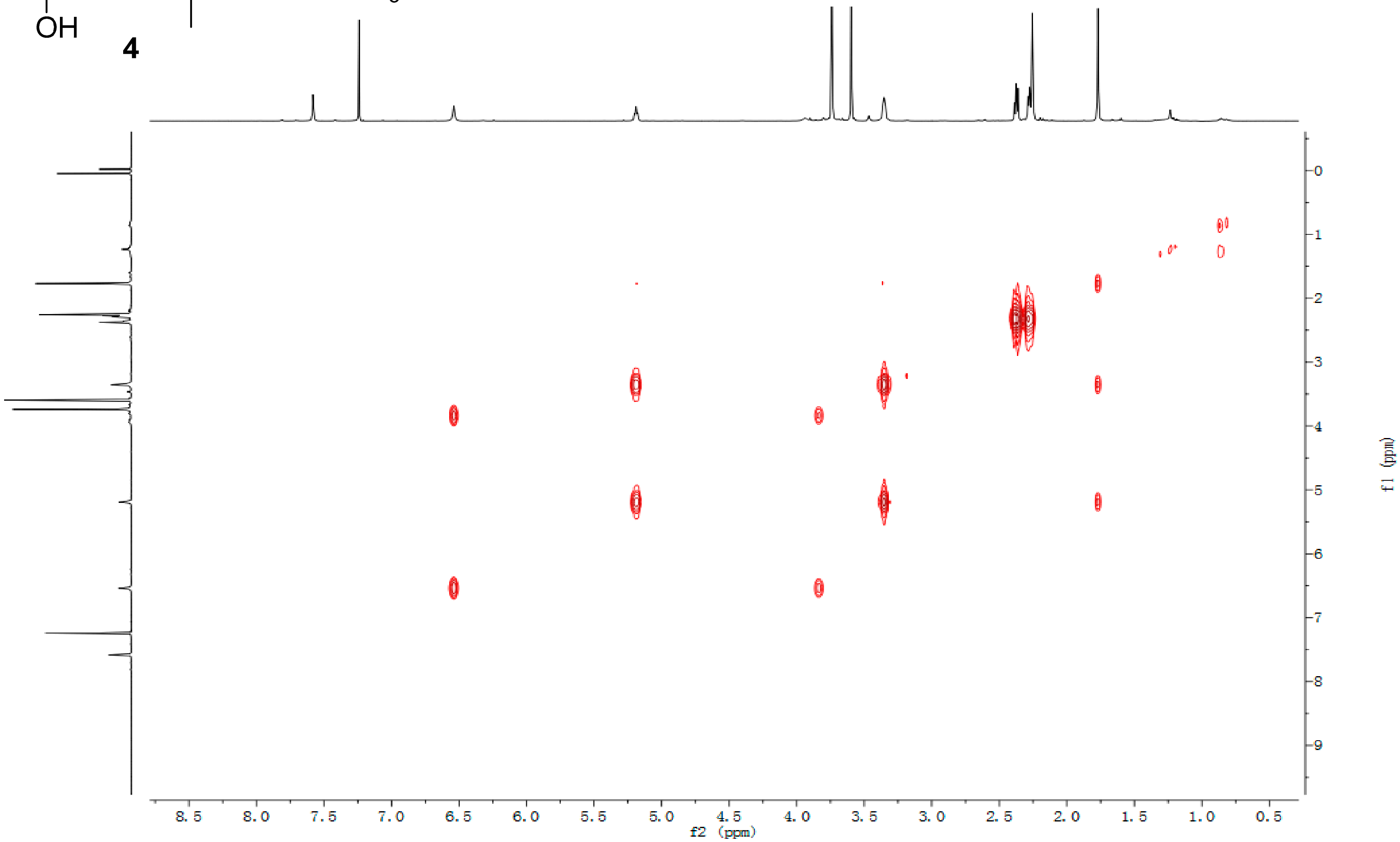

Figure S32. ^{1}H–^{1}H COSY spectrum of compound **4** (Recorded in CD_3OD)

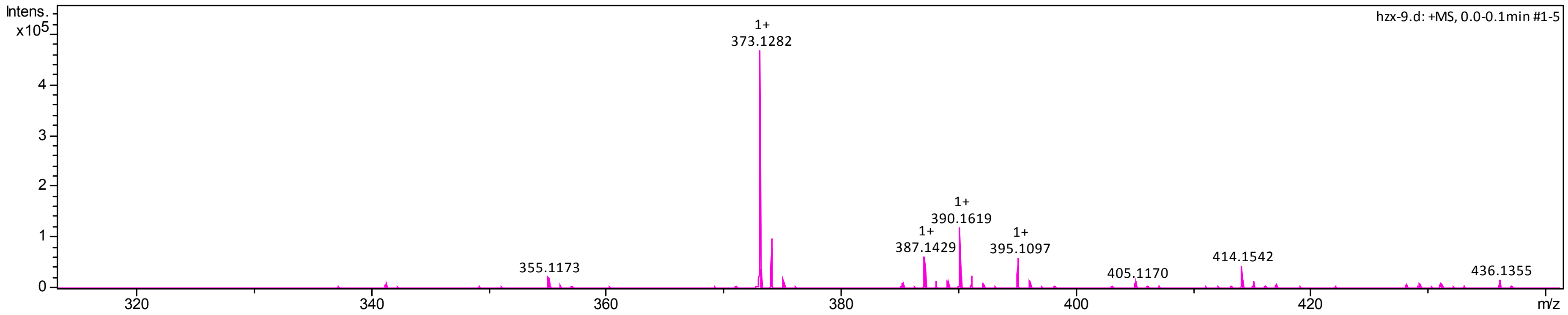

Figure S33. HRESIMS spectrum of compound **4**

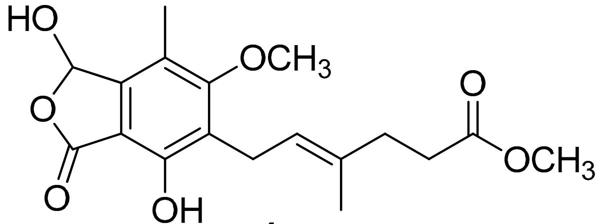

4

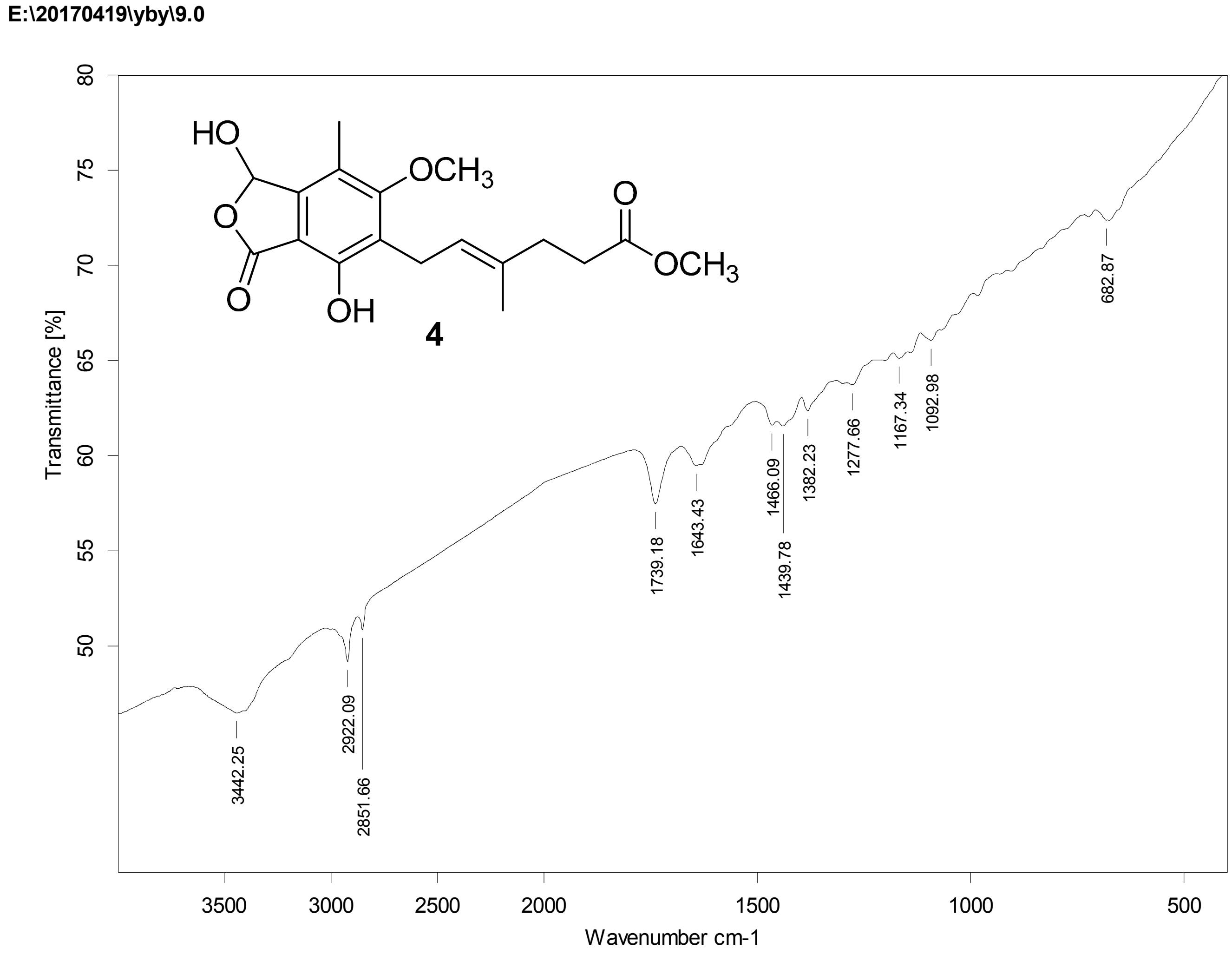

Figure S34. IR spectrum of compound **4**

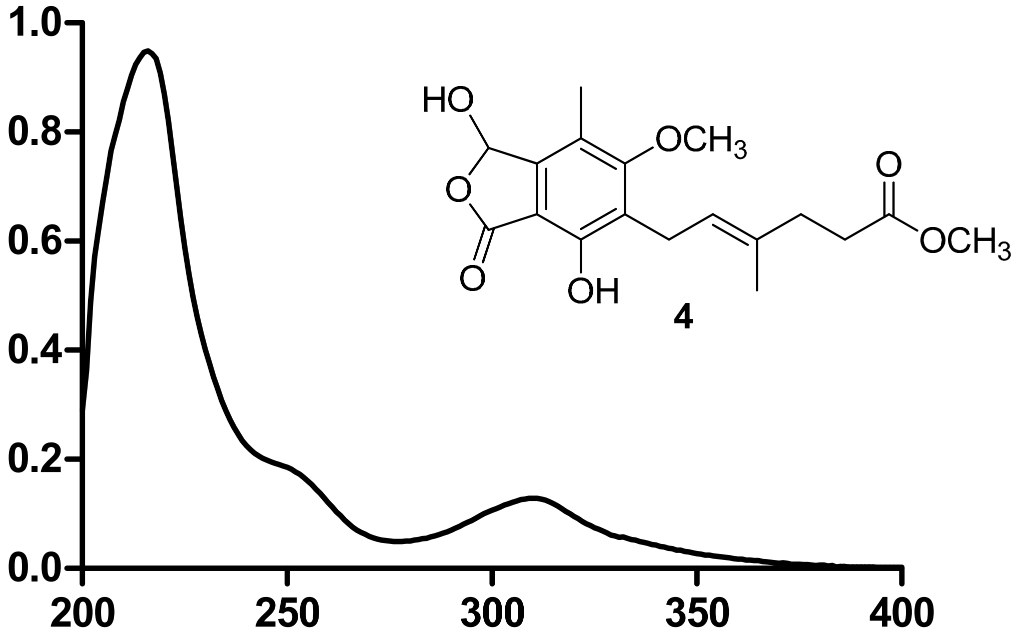

Figure S35. UV spectrum of compound **4**

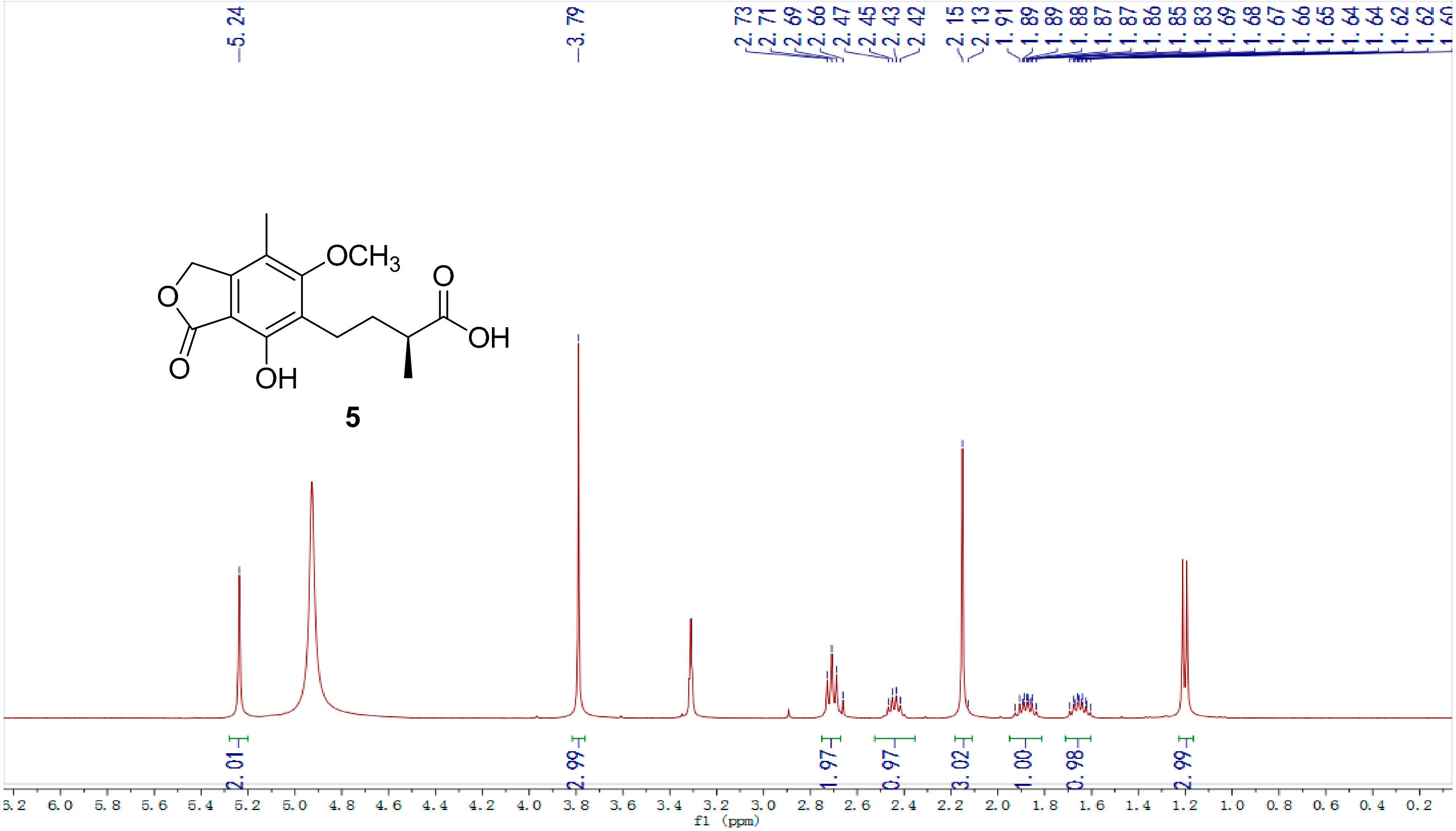

Figure S36. ^{1}H NMR spectrum of compound **5** (Recorded in CD_3OD)

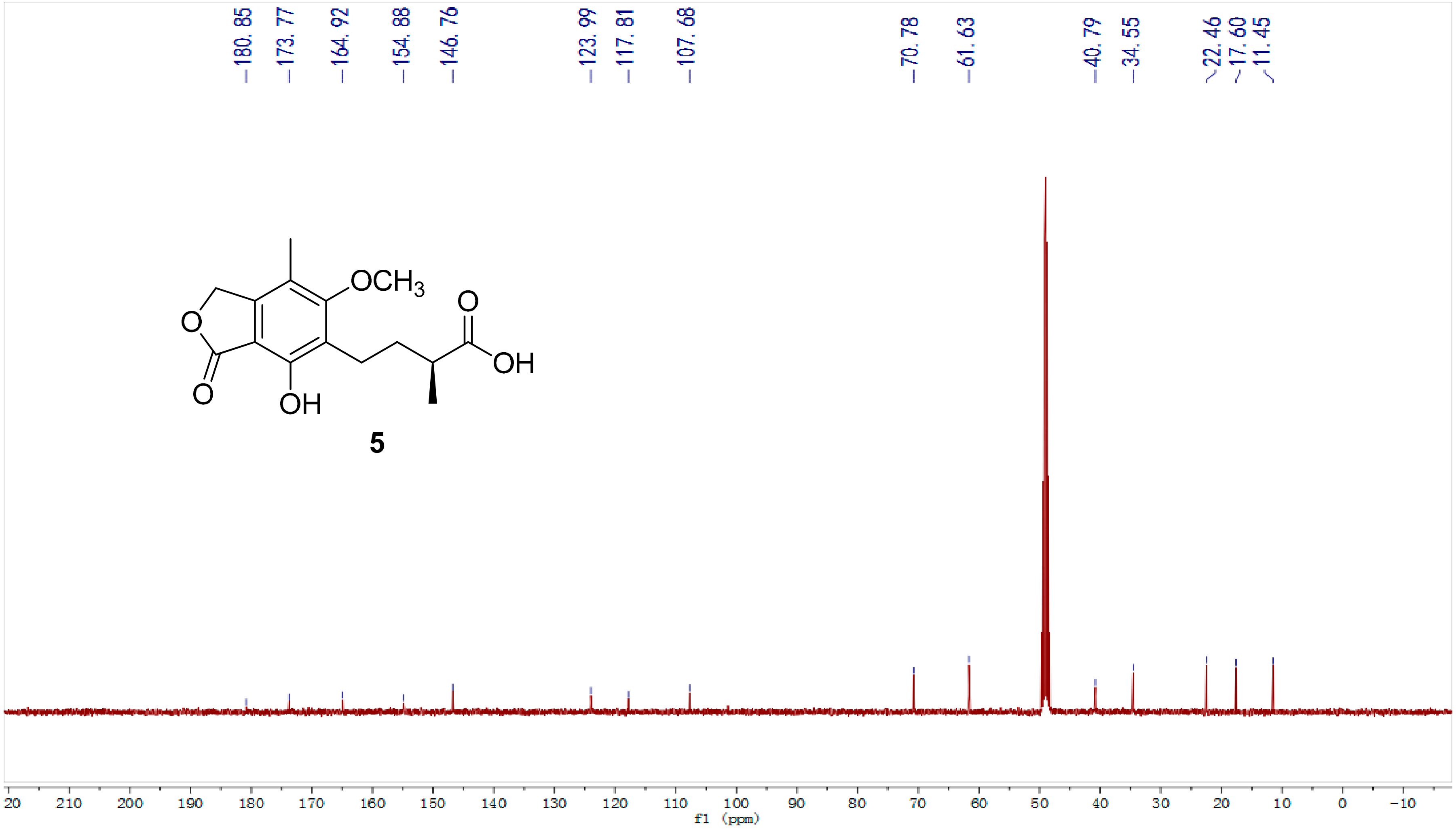

Figure S37. ^{13}C NMR spectrum of compound **5** (Recorded in CD_3OD)

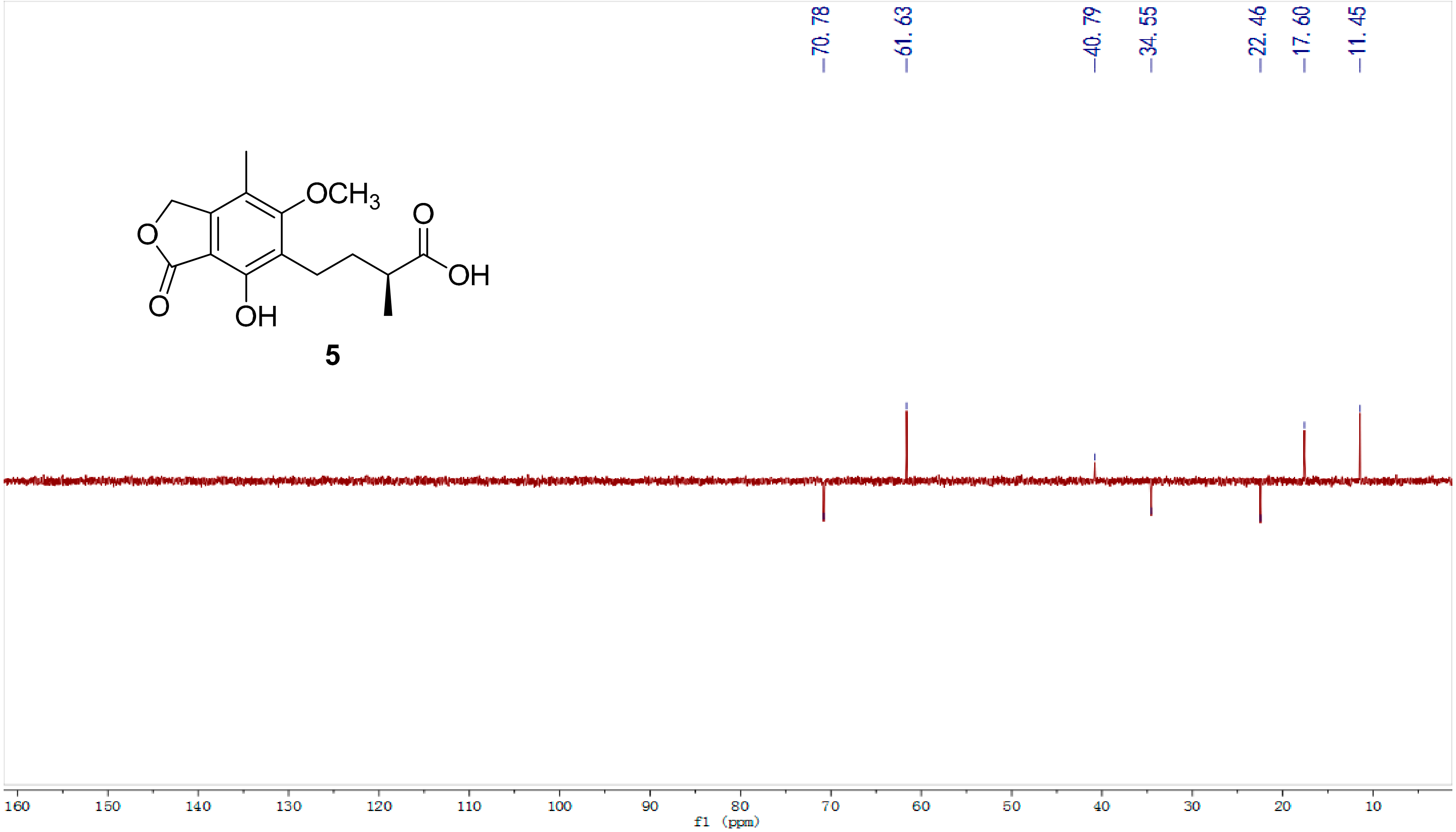

Figure S38. DEPT spectrum of compound **5** (Recorded in CD_3OD)

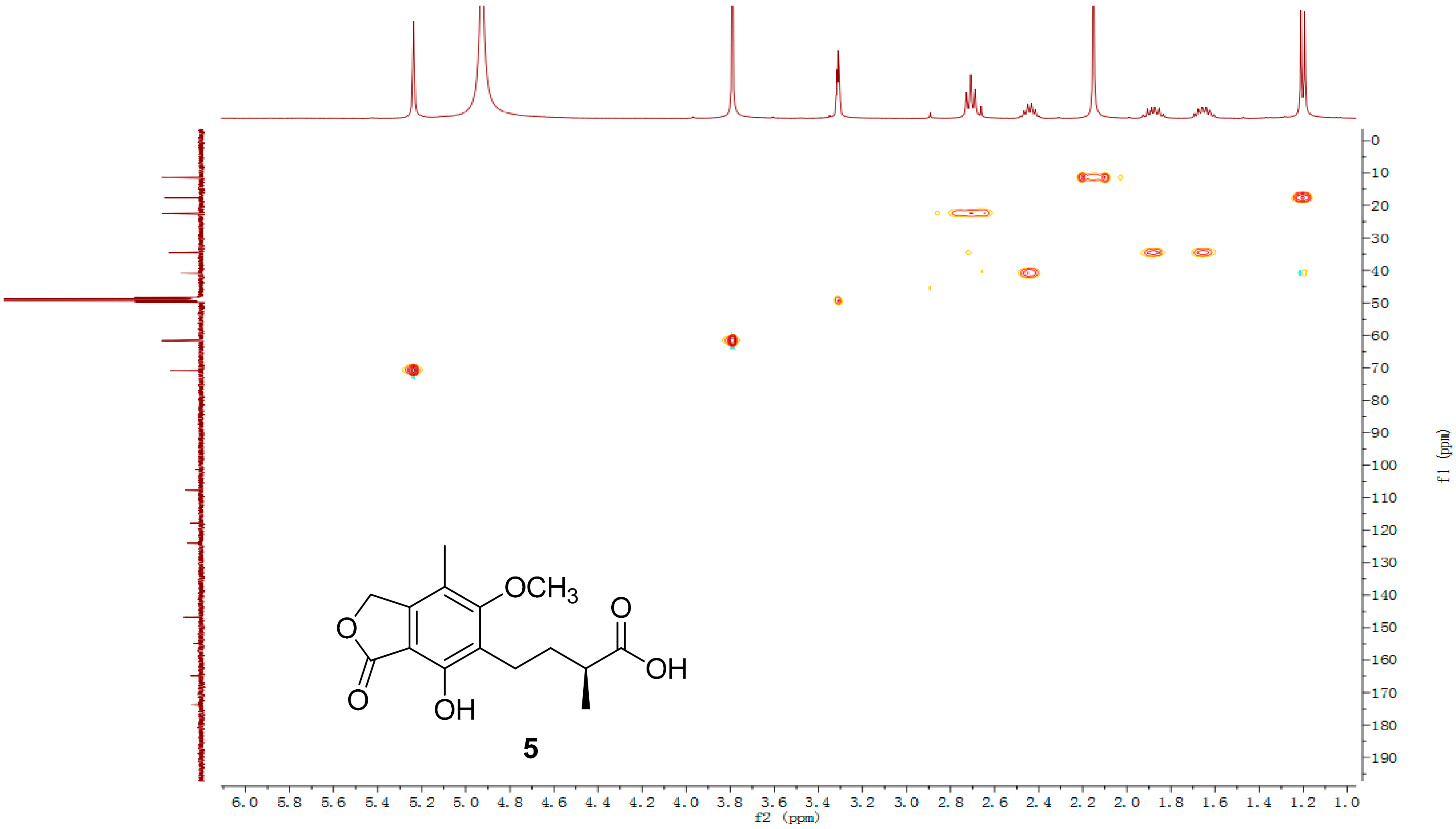

Figure S39. HSQC spectrum of compound **5** (Recorded in CD_3OD)

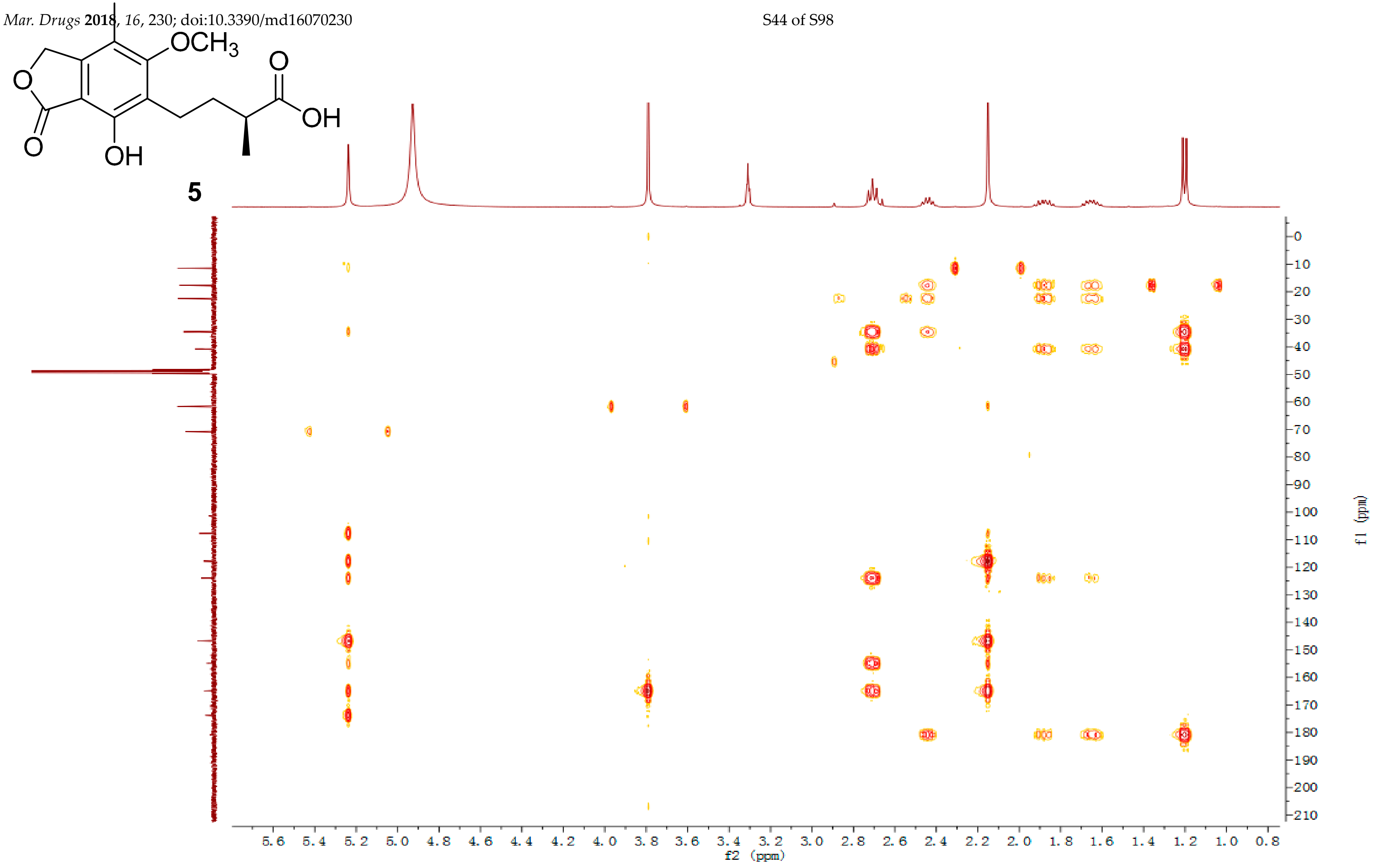

Figure S40. HMBC spectrum of compound **5** (Recorded in CD3OD)

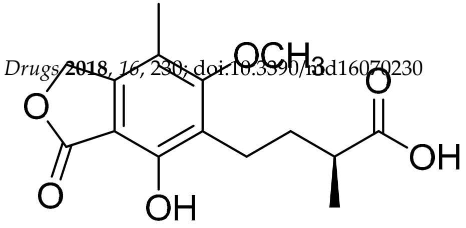

5

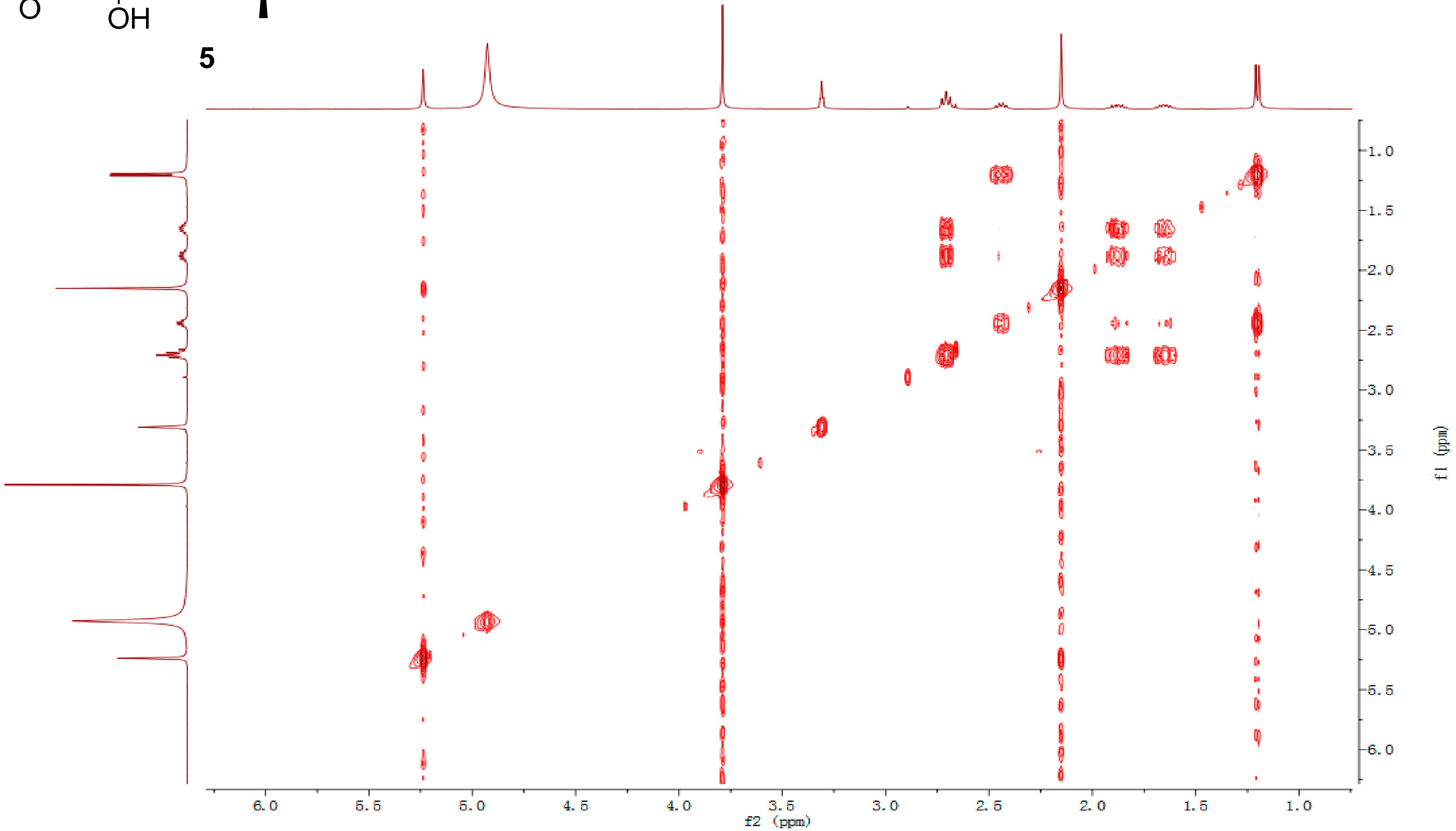

Figure S41. ^{1}H–^{1}H COSY spectrum of compound **5** (Recorded in CD_3OD)

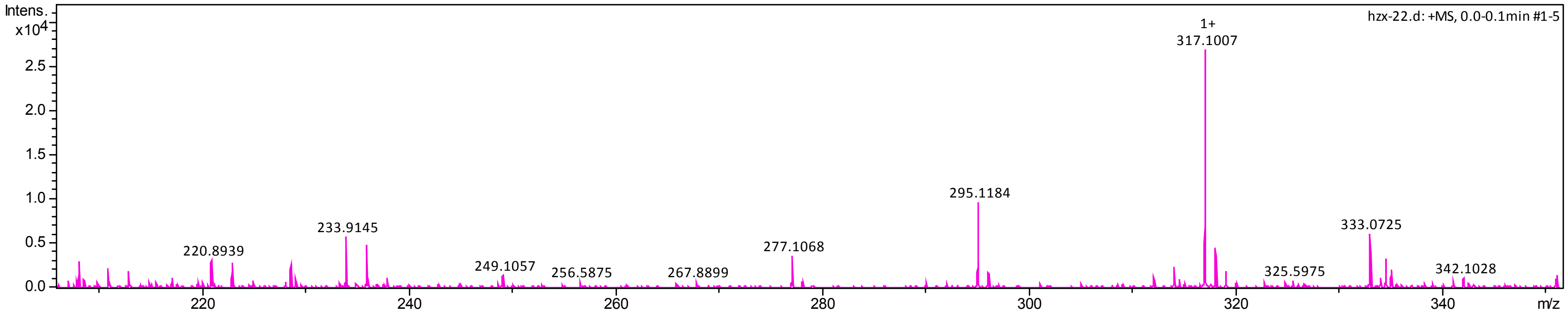

Figure S42. HRESIMS spectrum of compound **5**

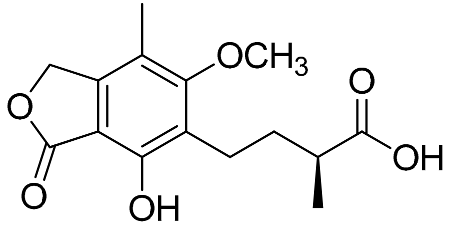

5

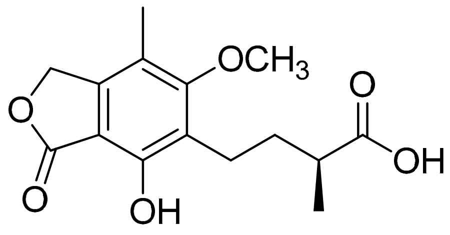

5

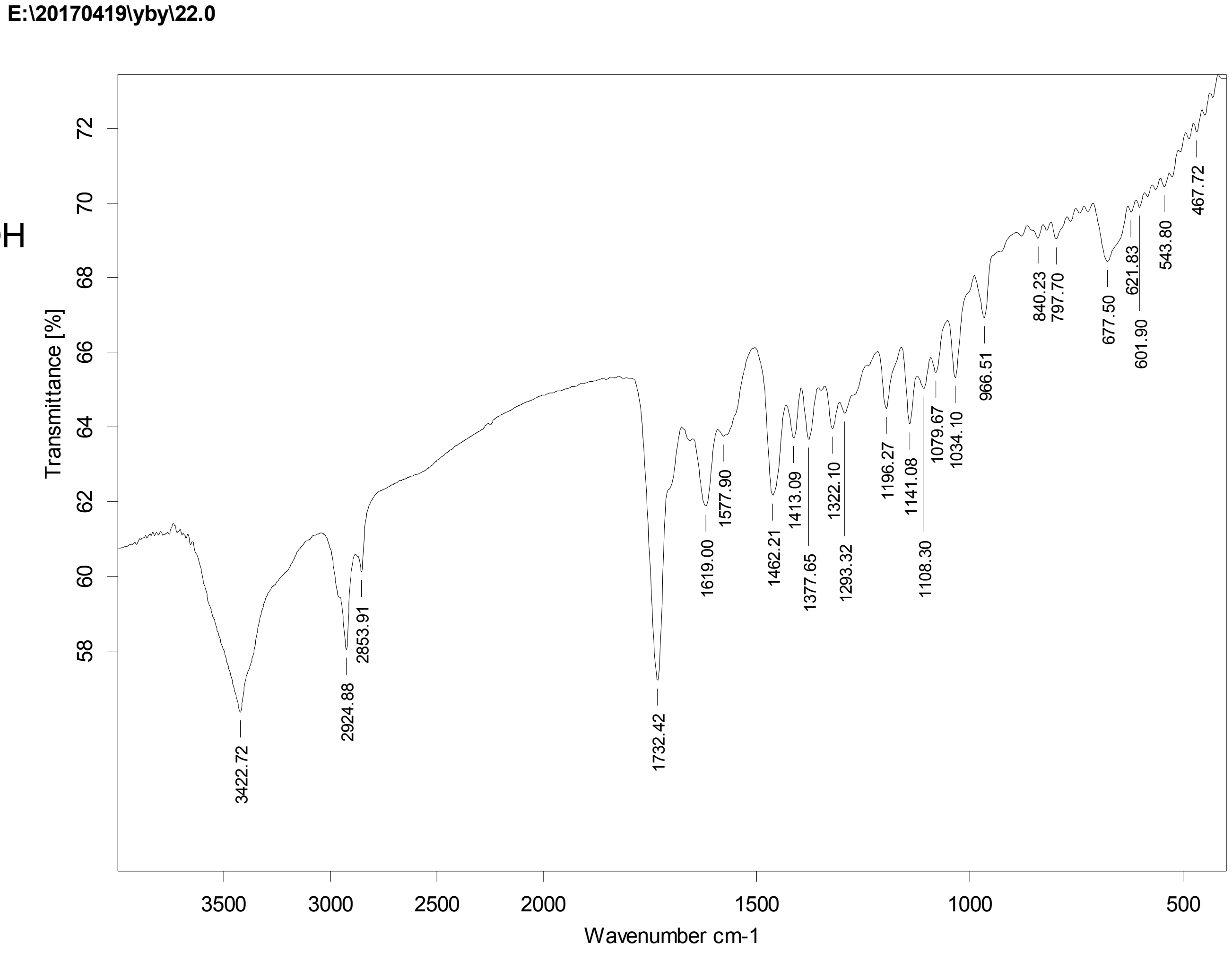

Figure S43. IR spectrum of compound **5**

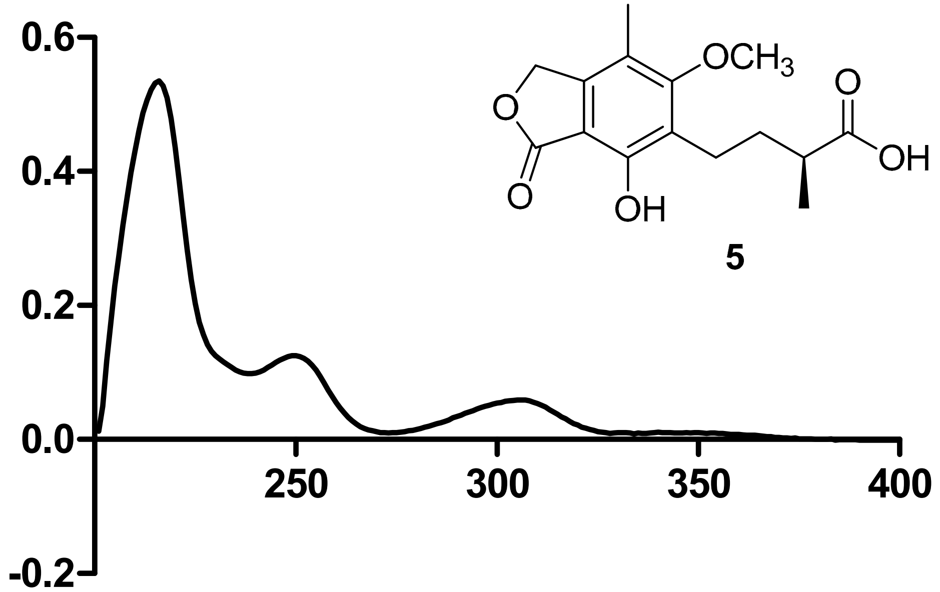

Figure S44. UV spectrum of compound **5**

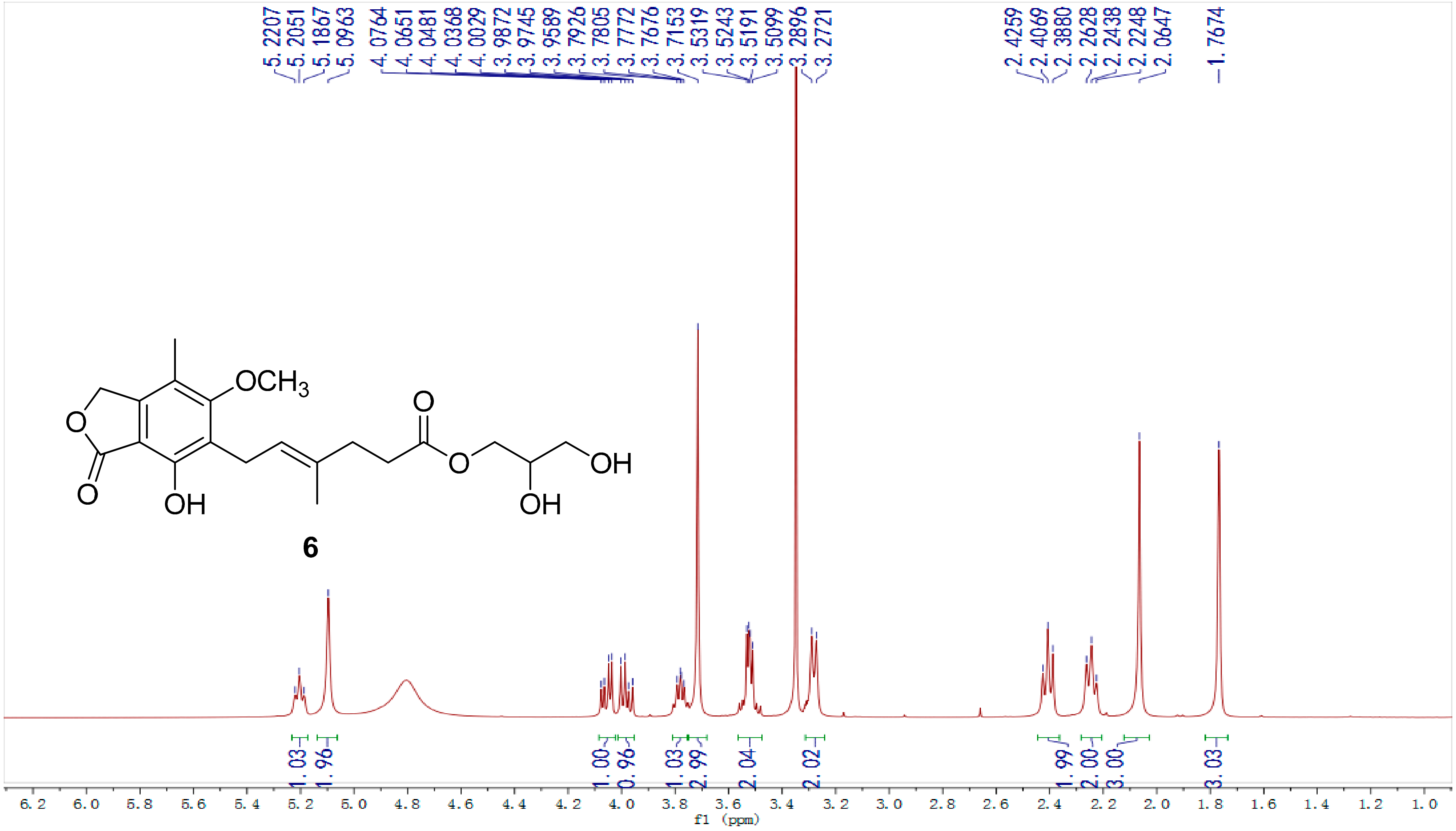

Figure S45. ^{1}H NMR spectrum of compound **6** (Recorded in CD_3OD)

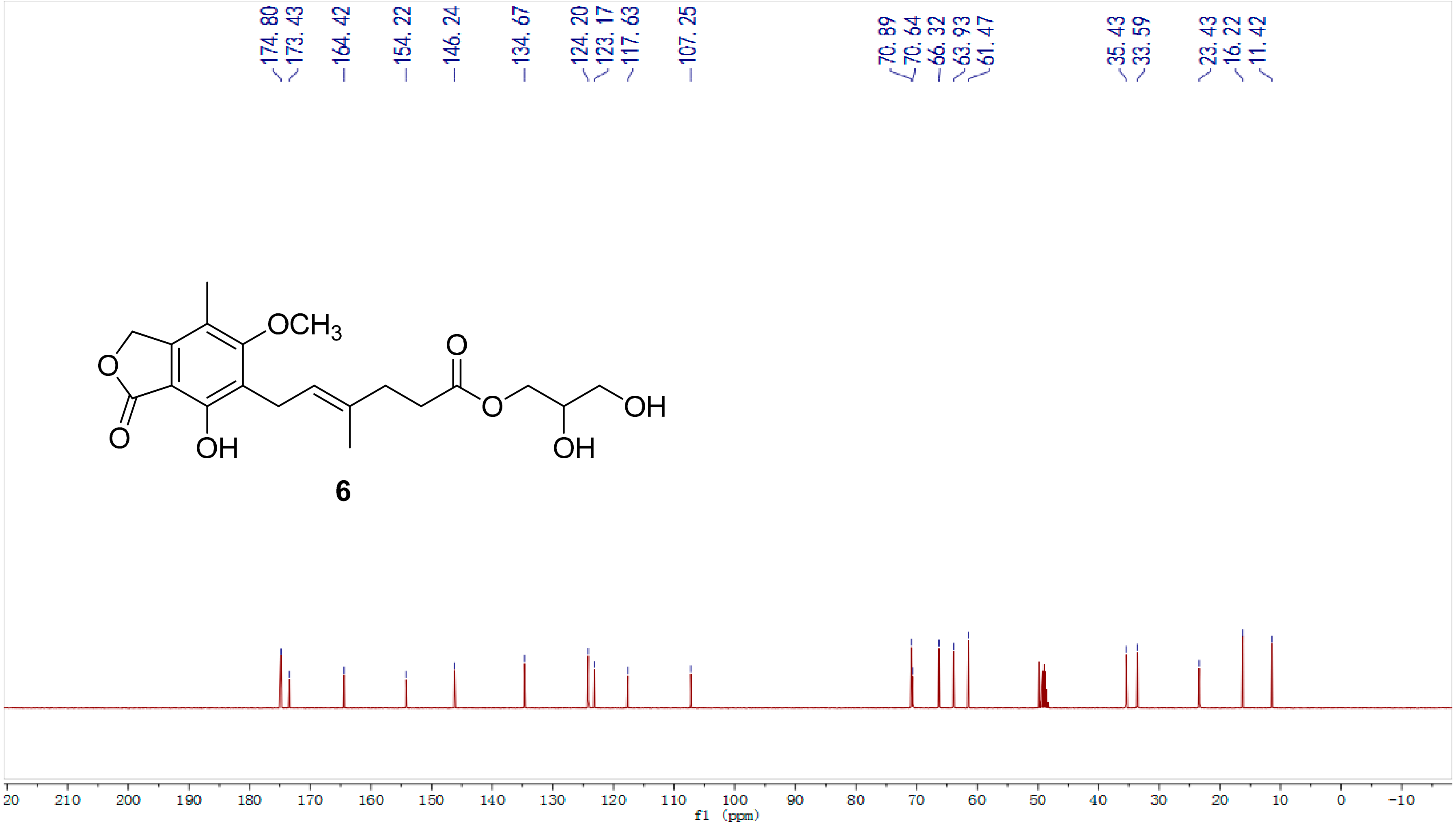

Figure S46. ^{13}C NMR spectrum of compound **6** (Recorded in CD_3OD)

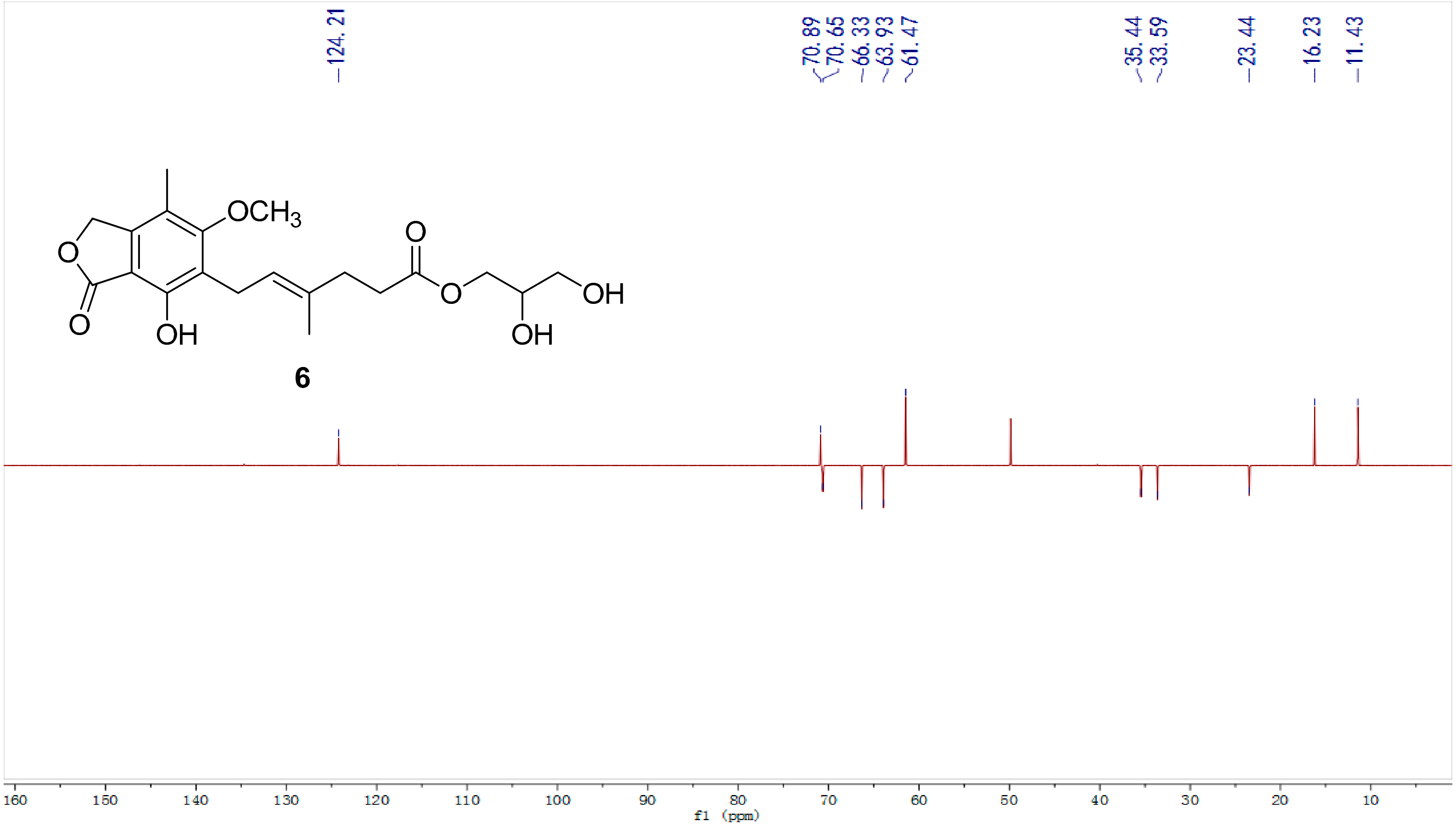

Figure S47. DEPT spectrum of compound **6** (Recorded in CD_3OD)

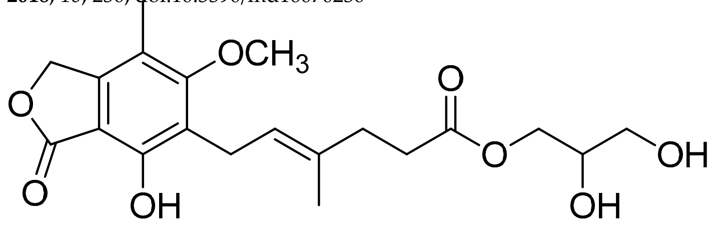

6

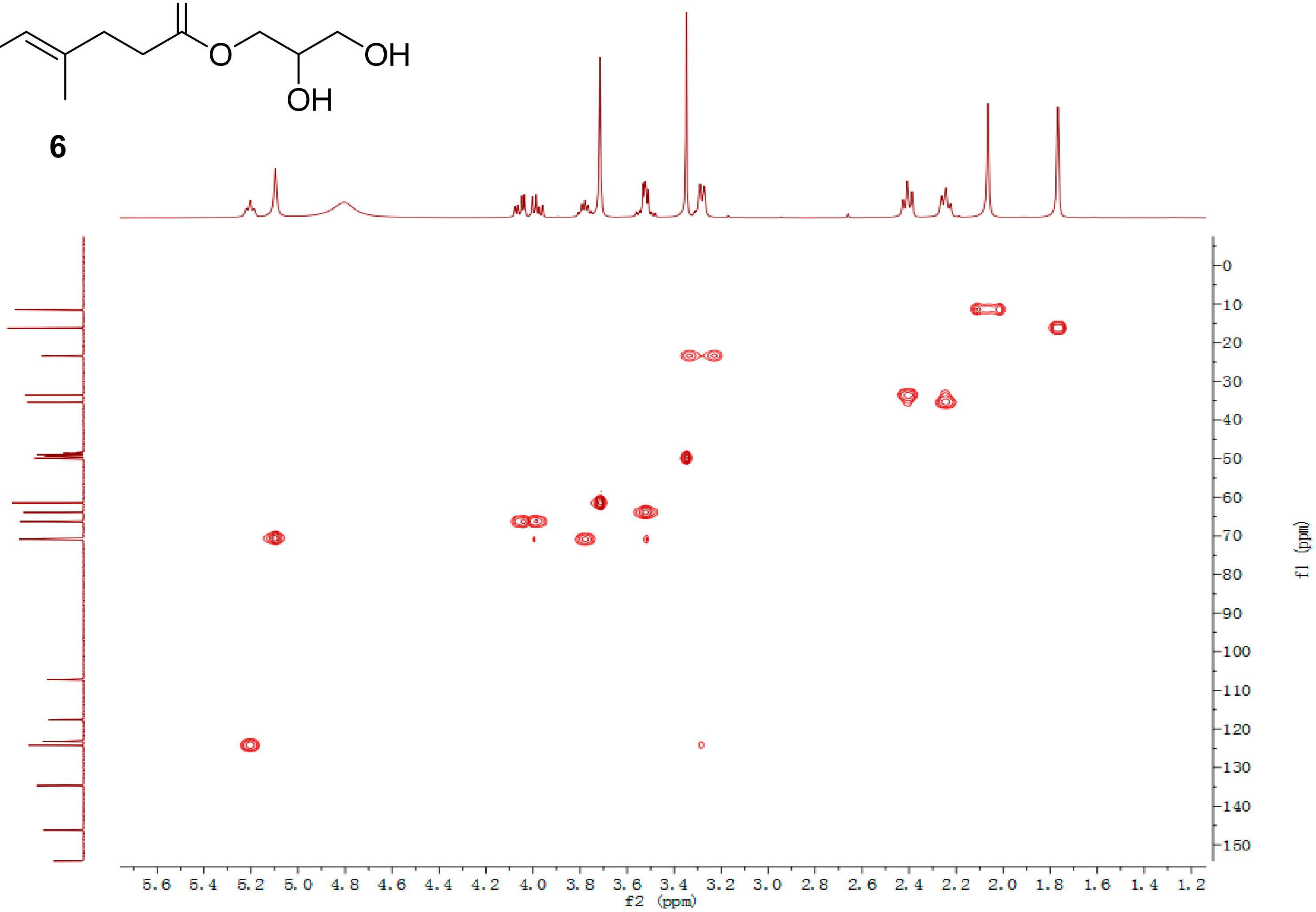

Figure S48. HSQC spectrum of compound **6** (Recorded in CD_3OD)

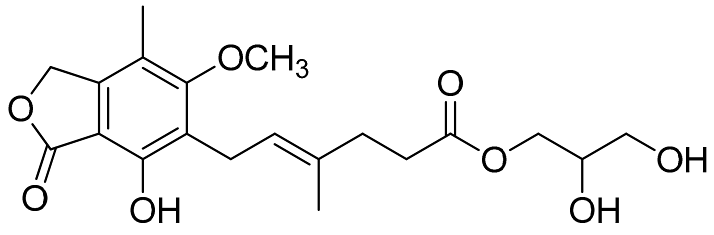

6

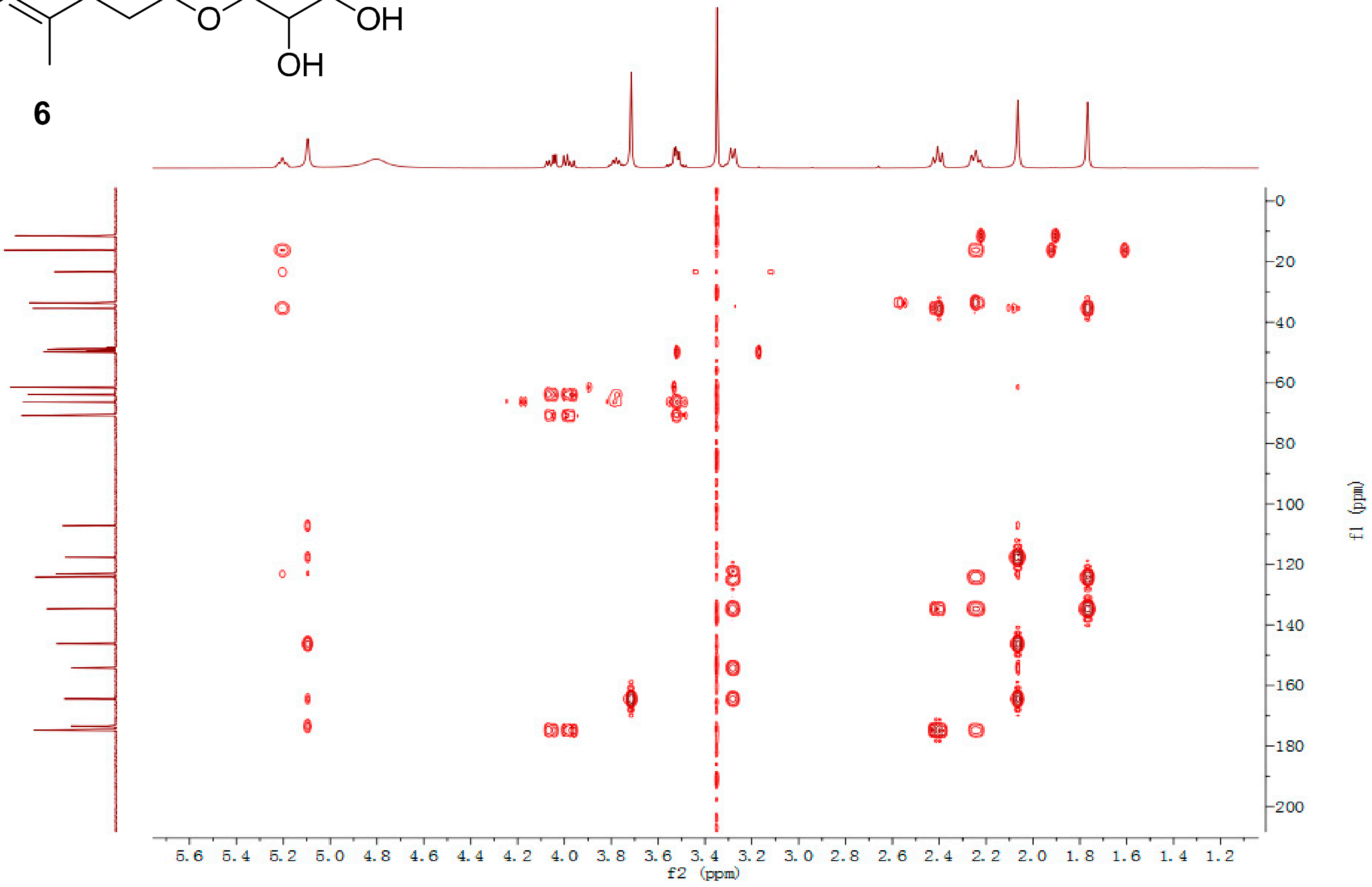

Figure S49. HMBC spectrum of compound **6** (Recorded in CD_3OD)

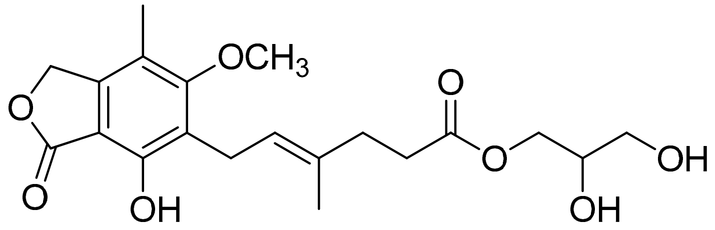

6

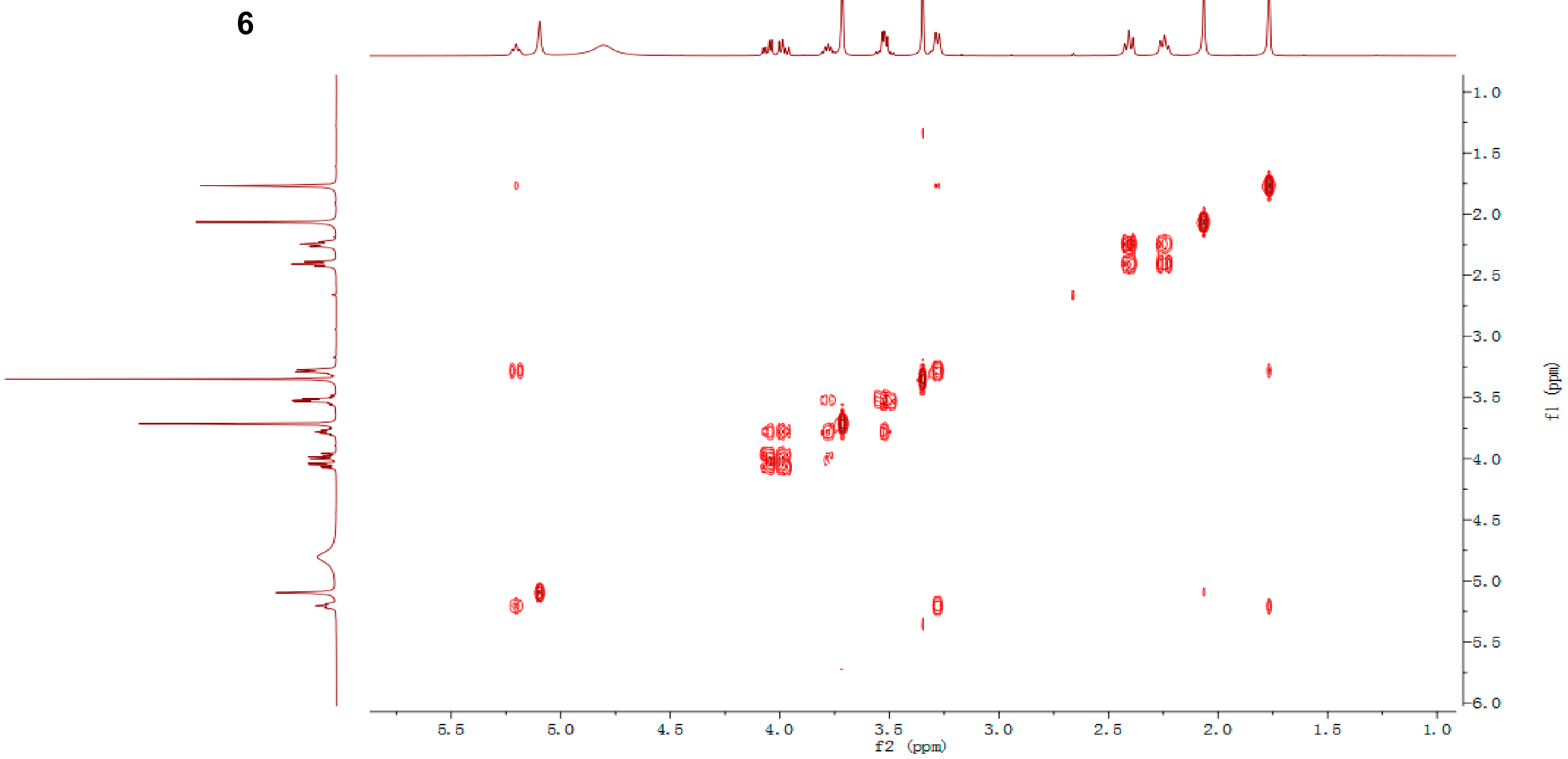

Figure S50. $^{1}H–^{1}H$ COSY spectrum of compound **6** (Recorded in CD_3OD)

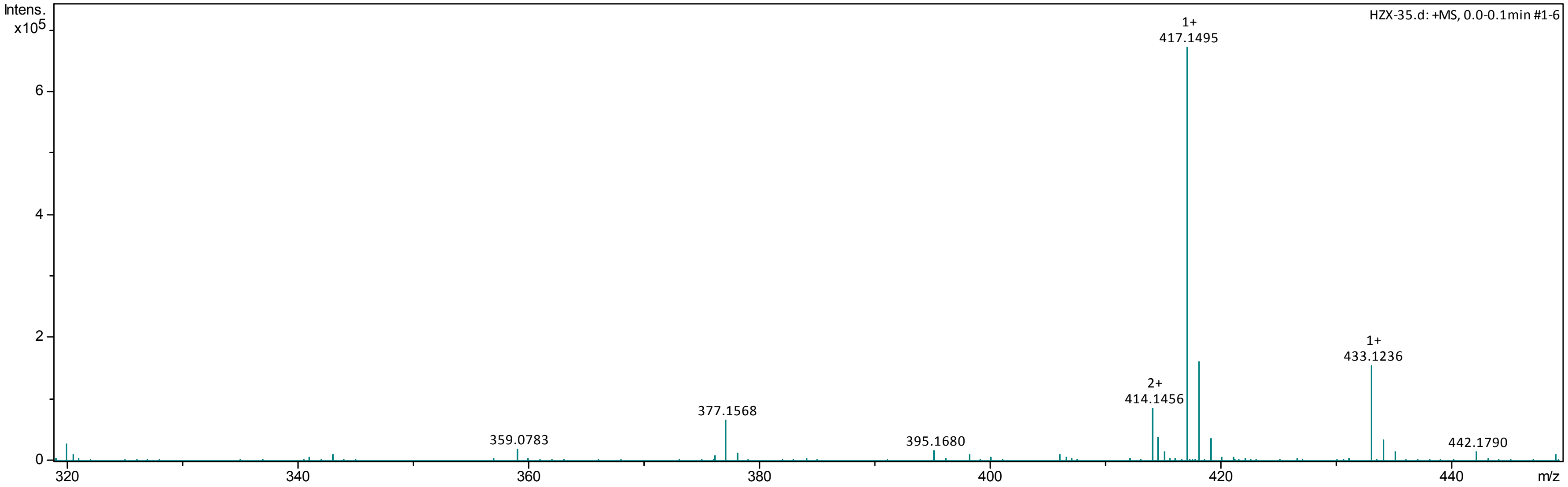

Figure S51. HRESIMS spectrum of compound **6**

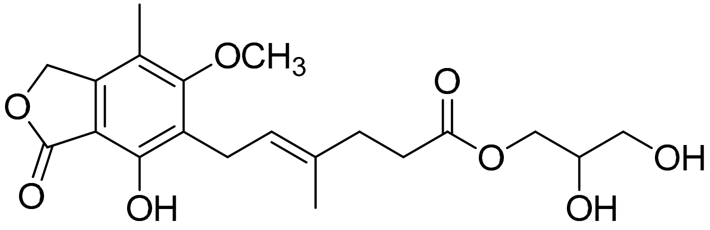

6

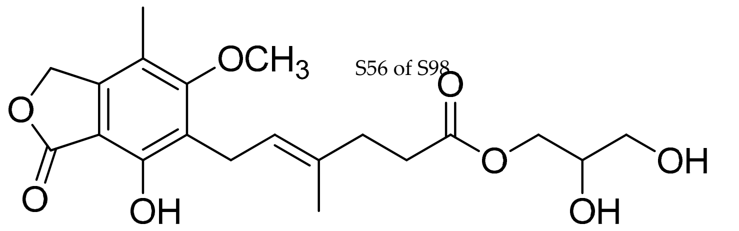

E:\20170419\yby\35.0

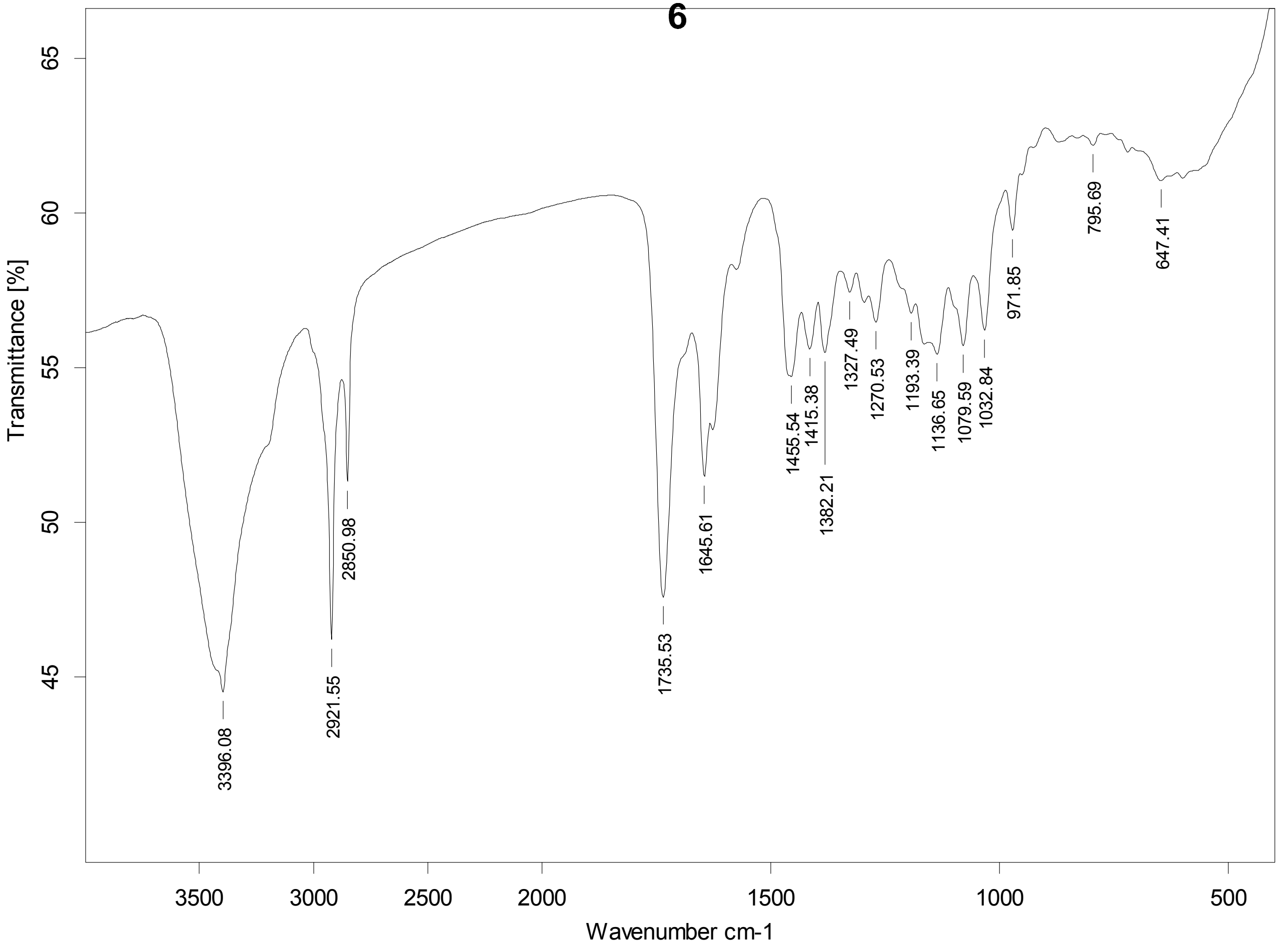

Figure S52. IR spectrum of compound **6**

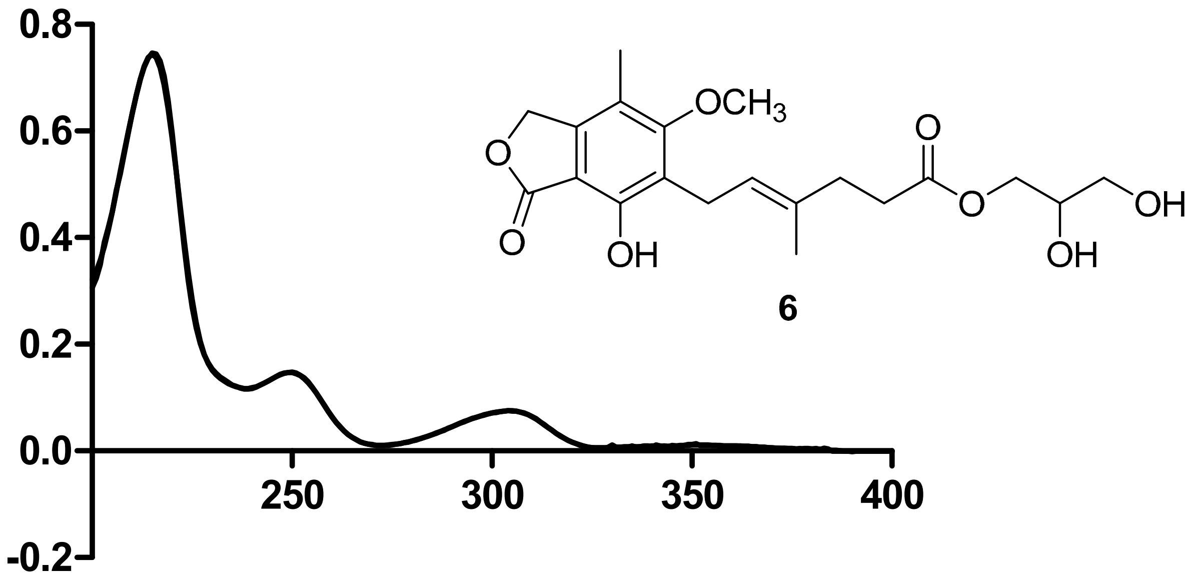

Figure S53. UV spectrum of compound **6**

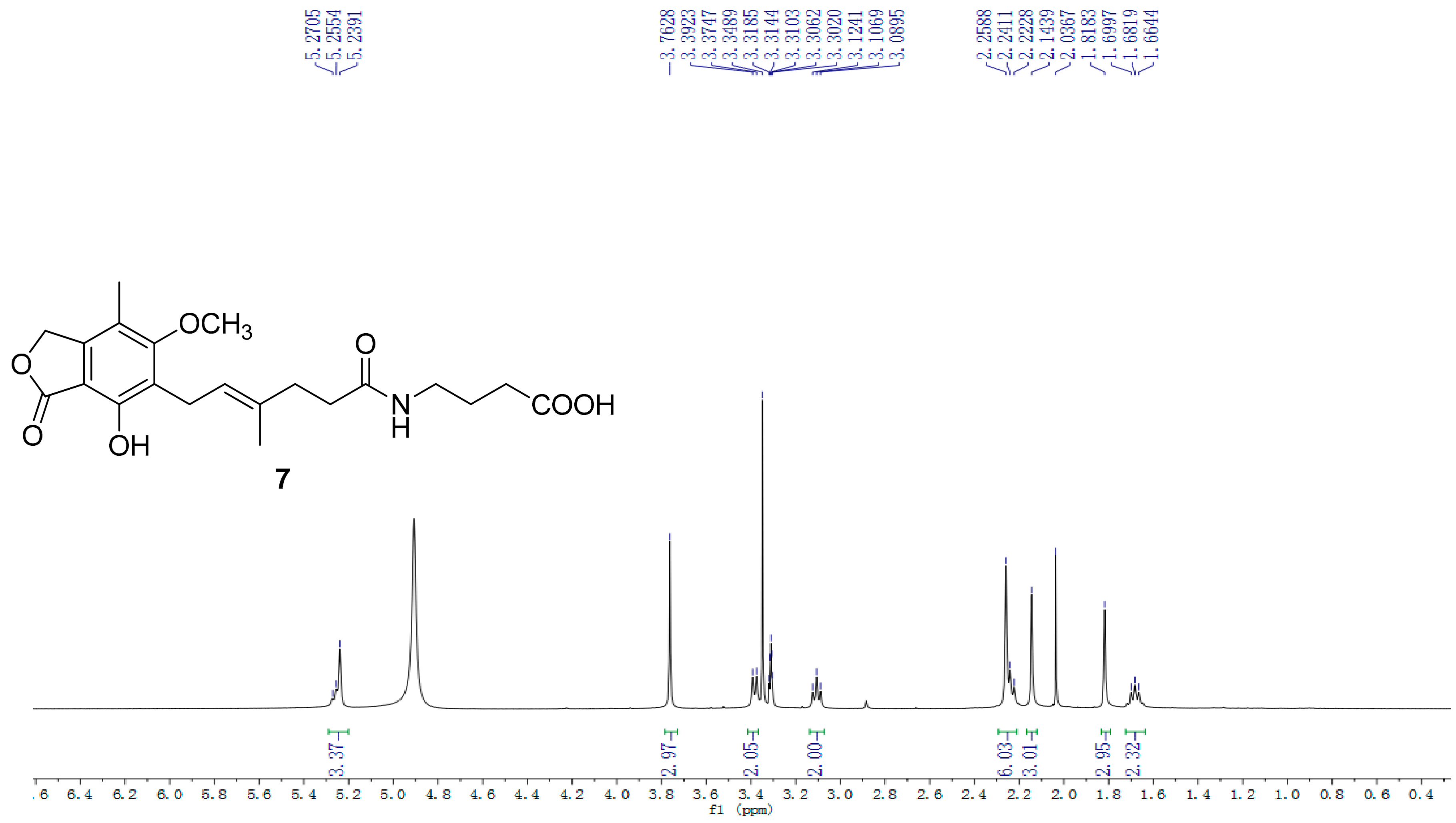

Figure S54. ¹H NMR spectrum of compound **7** (Recorded in CD3OD)

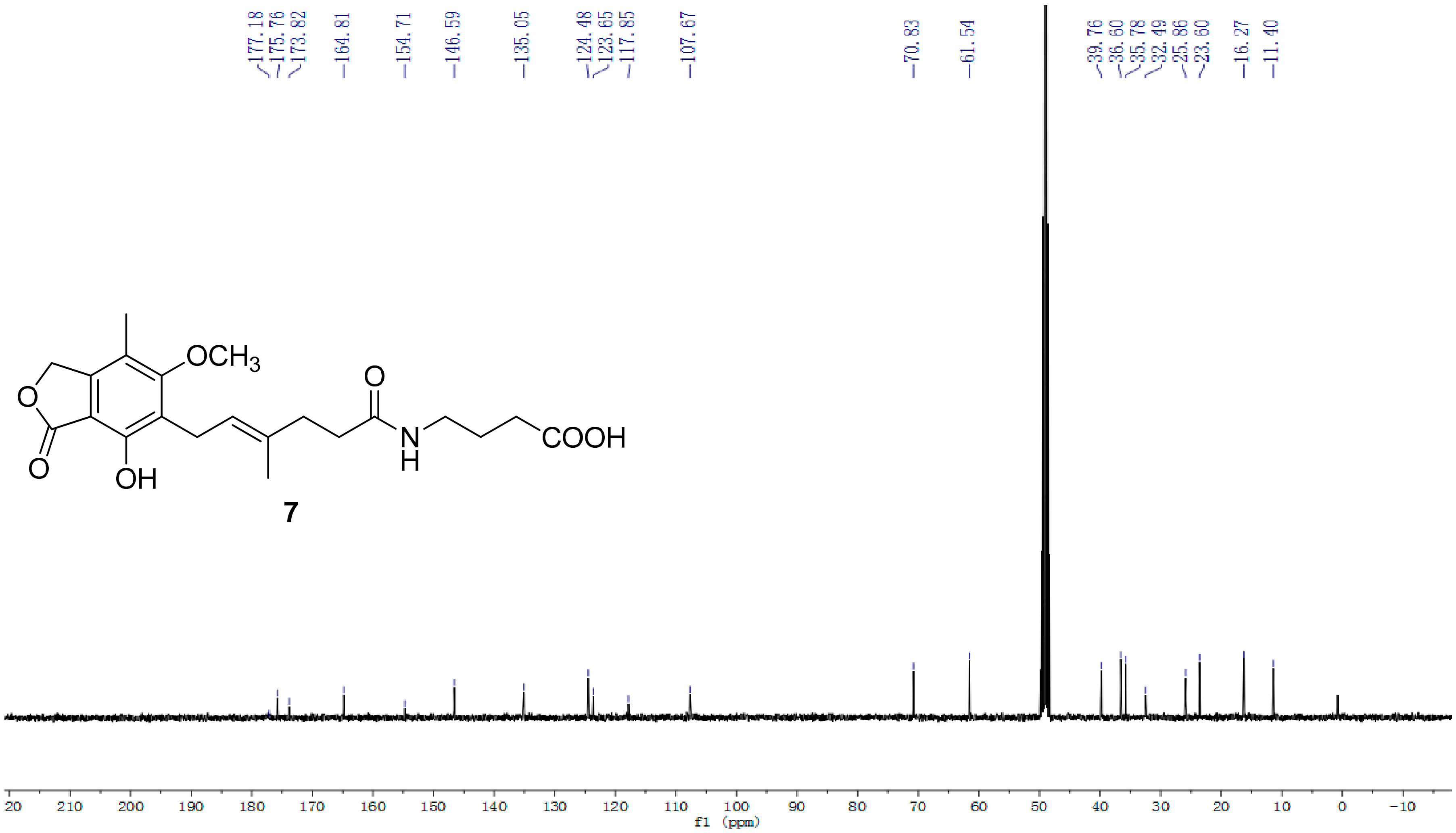

Figure S55. ^{13}C NMR spectrum of compound **7** (Recorded in CD_3OD)

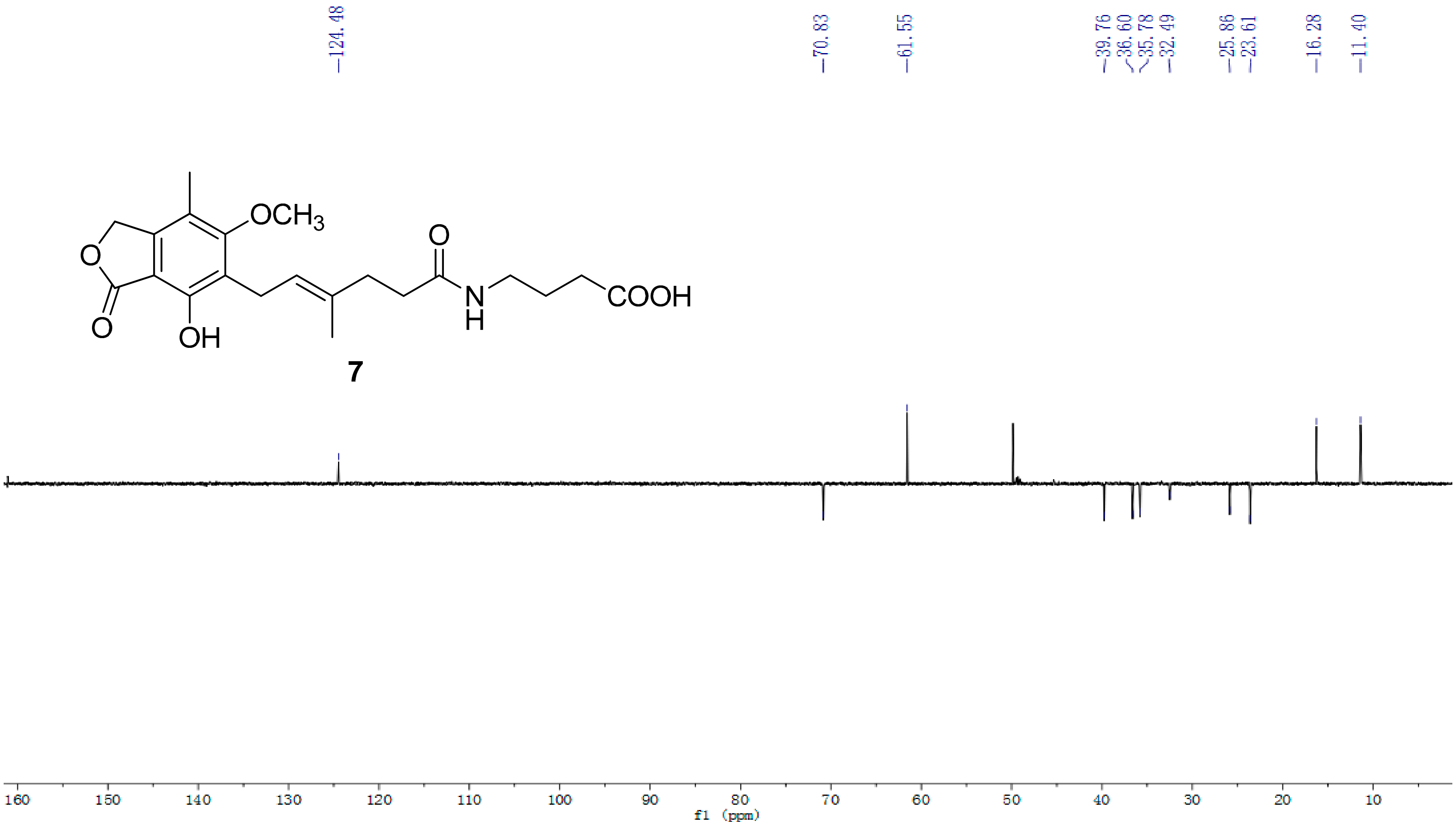

Figure S56. DEPT spectrum of compound **7** (Recorded in CD_3OD)

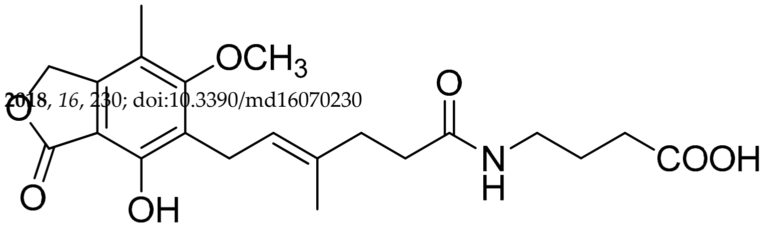

7

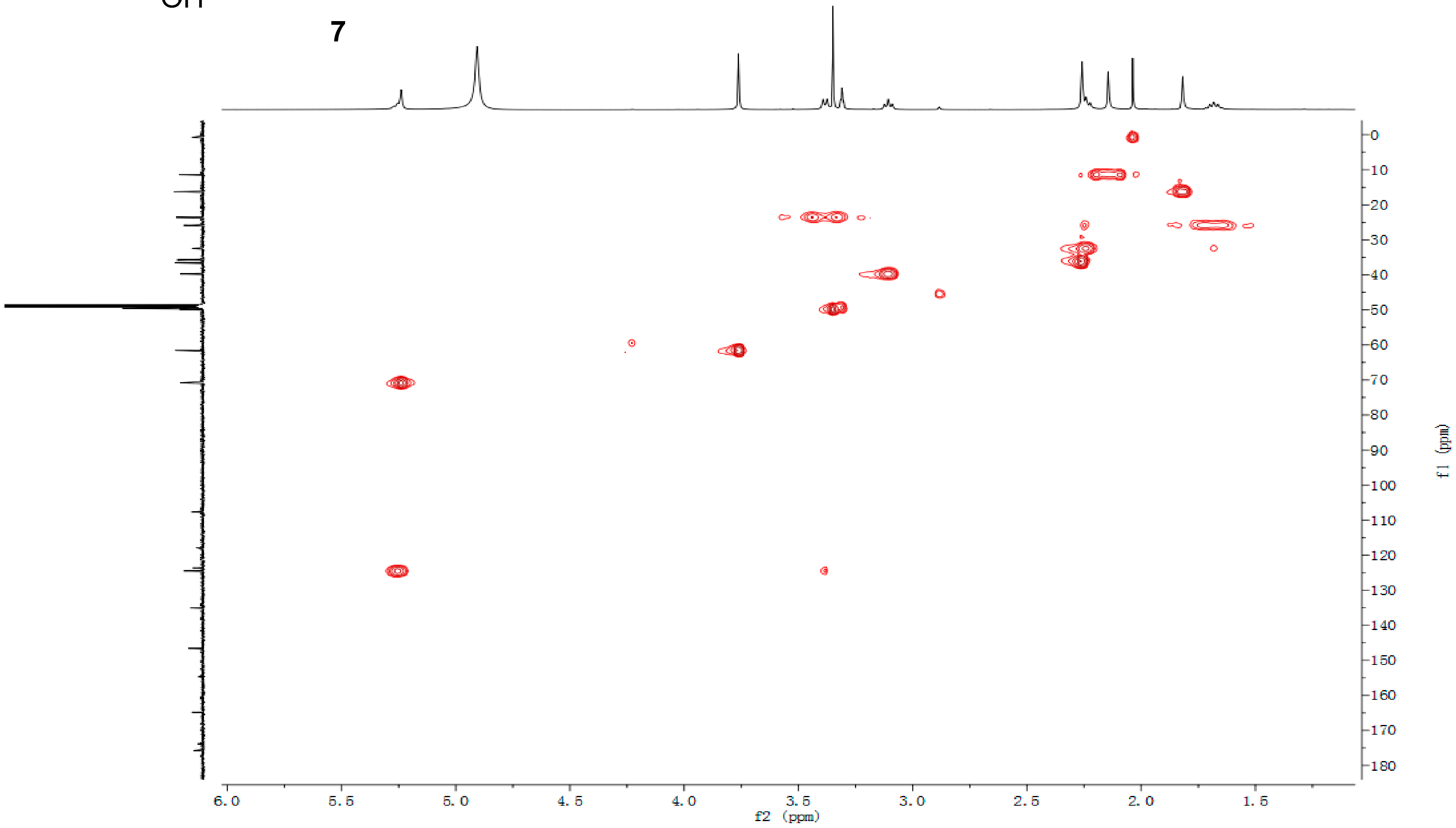

Figure S57. HSQC spectrum of compound **7** (Recorded in CD_3OD)

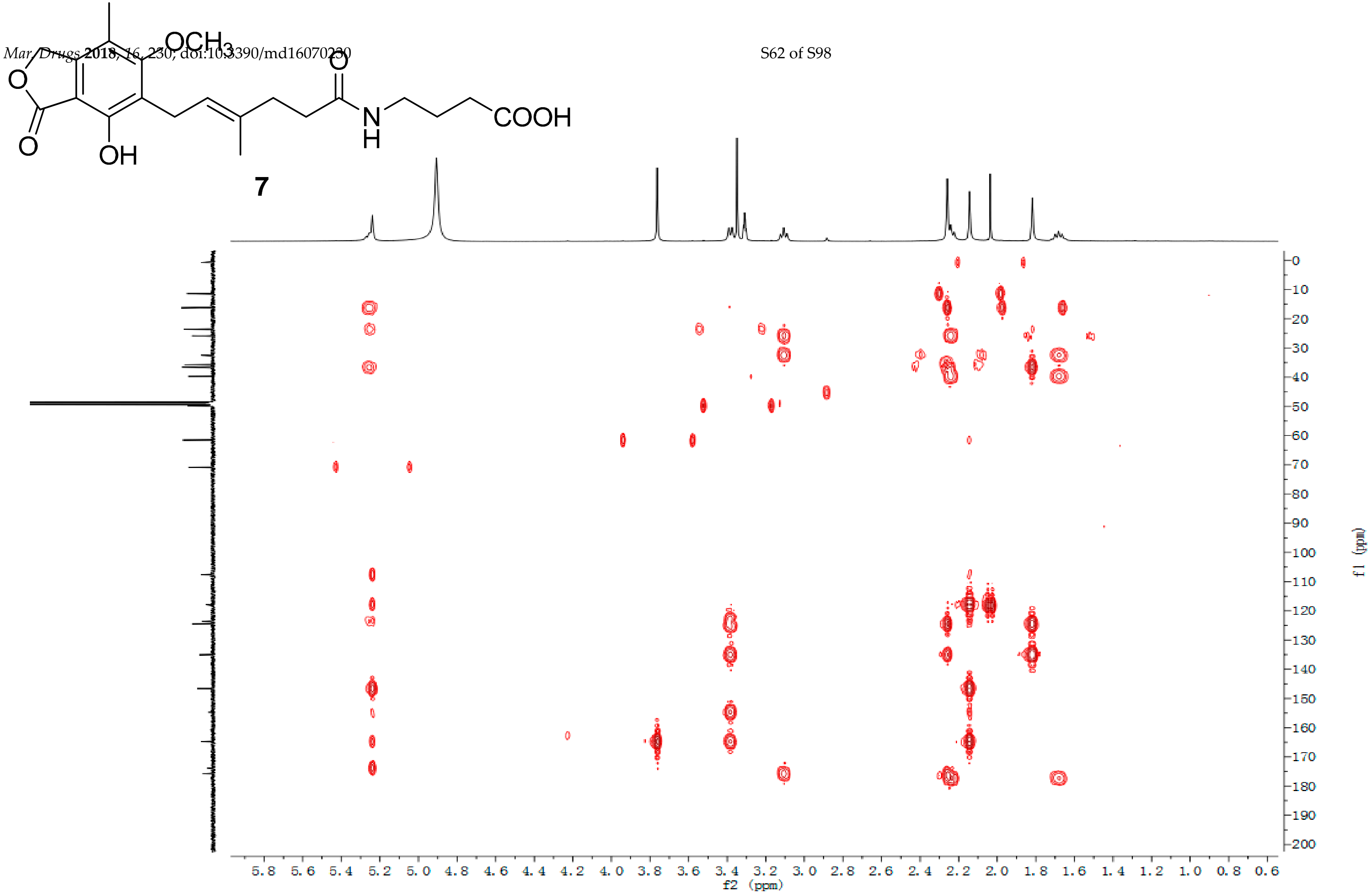

Figure S58. HMBC spectrum of compound **7** (Recorded in CD_3OD)

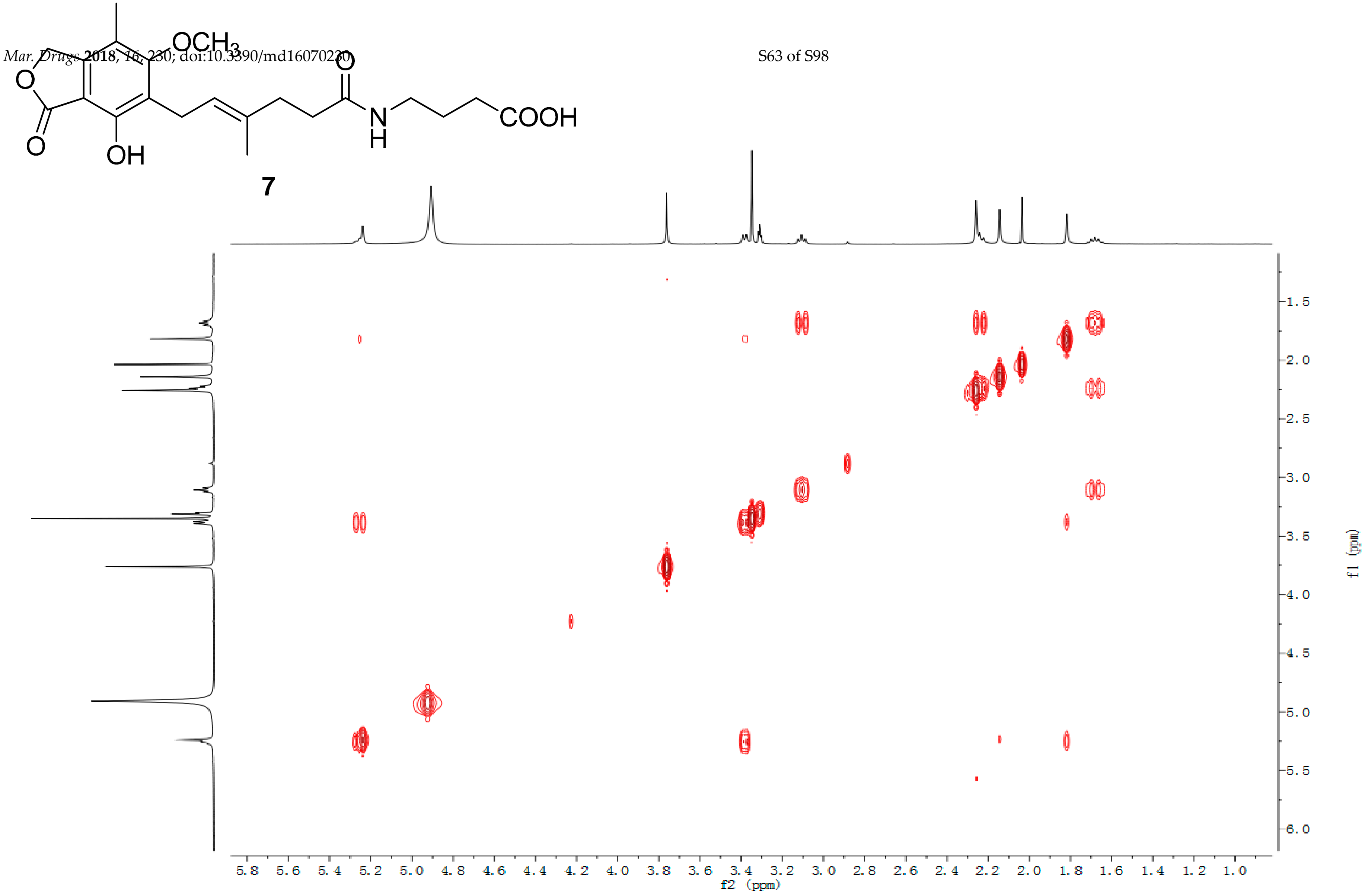

Figure S59. ^{1}H–^{1}H COSY spectrum of compound **7** (Recorded in CD3OD)

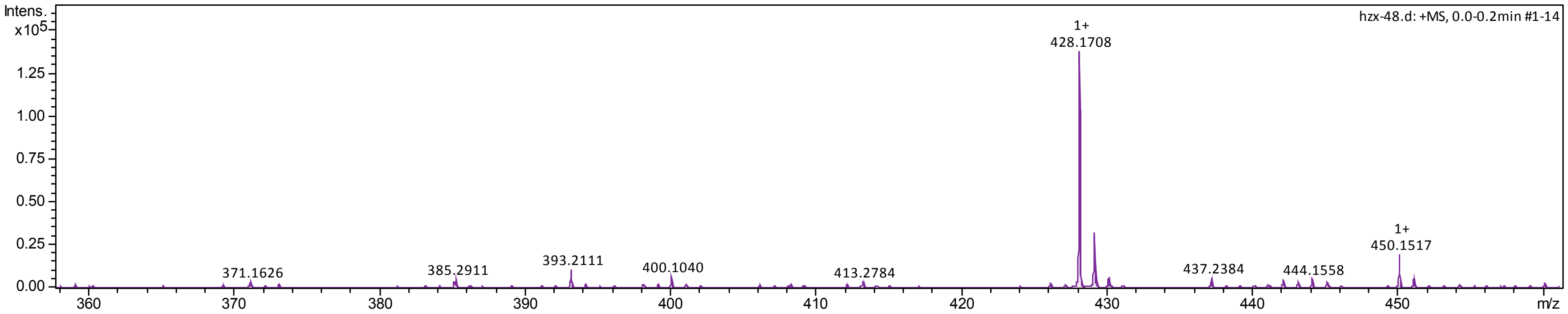

Figure S60. HRESIMS spectrum of compound **7**

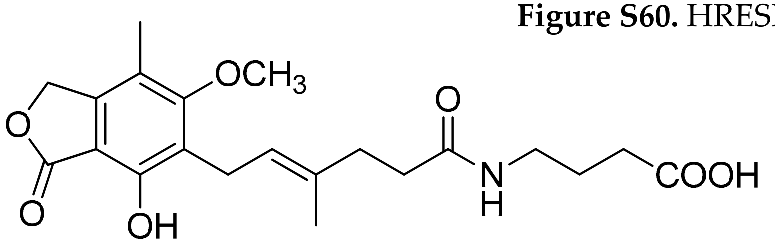

7

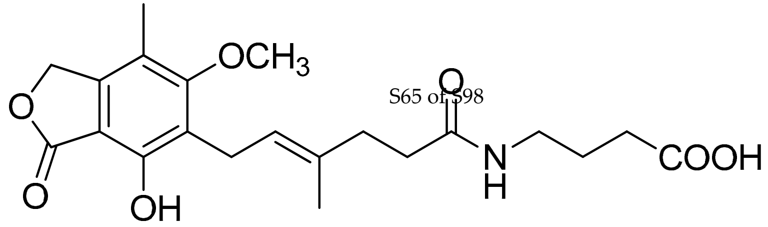

7

E:\20170419\yby\48.0

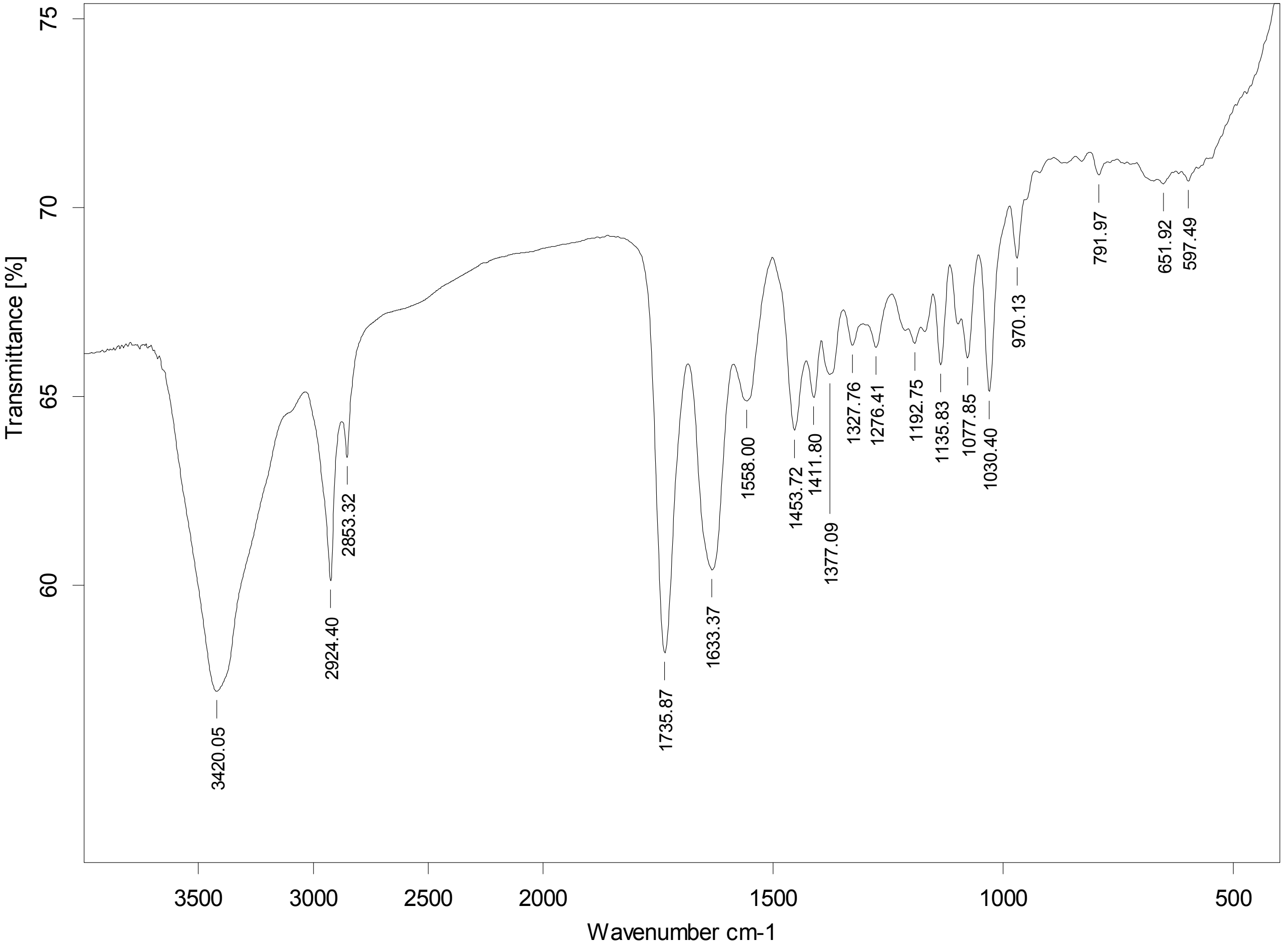

Figure S61. IR spectrum of compound **7**

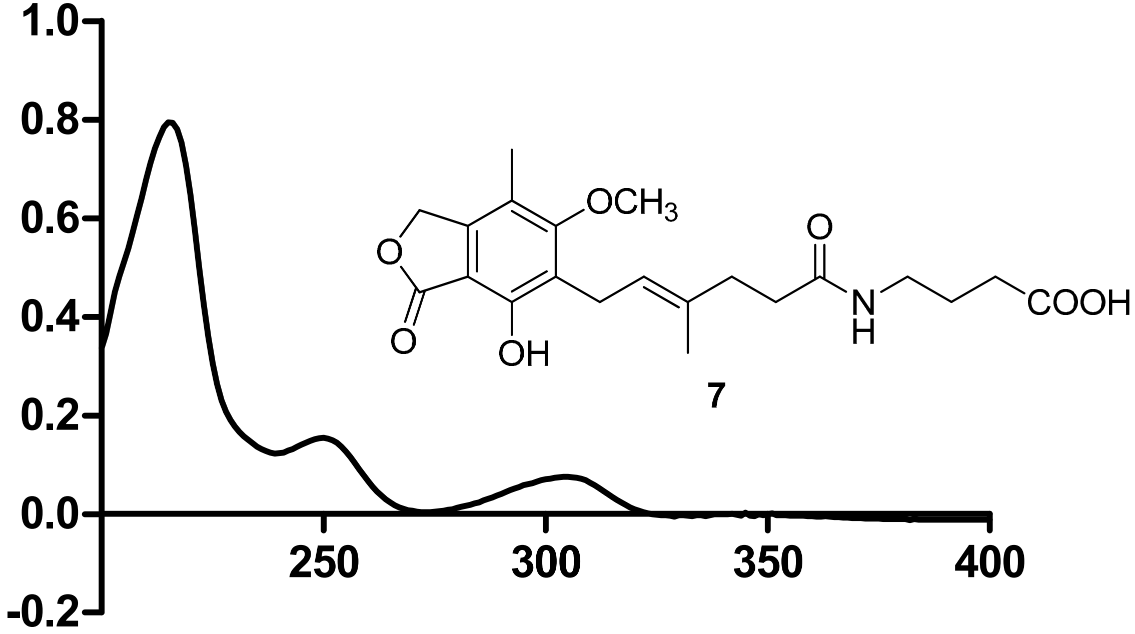

Figure S62. UV spectrum of compound **7**

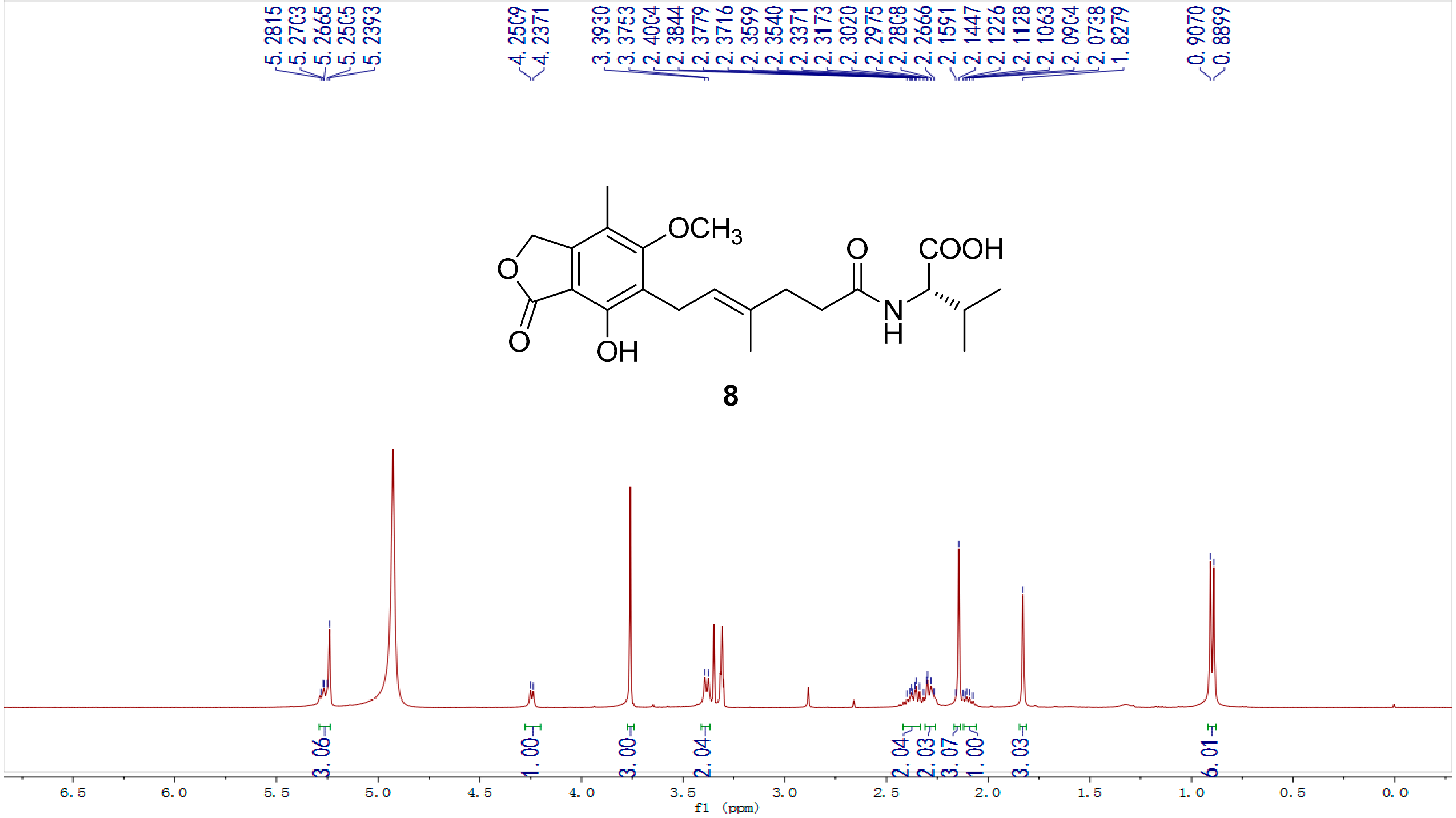

Figure S63. ^{1}H NMR spectrum of compound **8** (Recorded in CD_3OD)

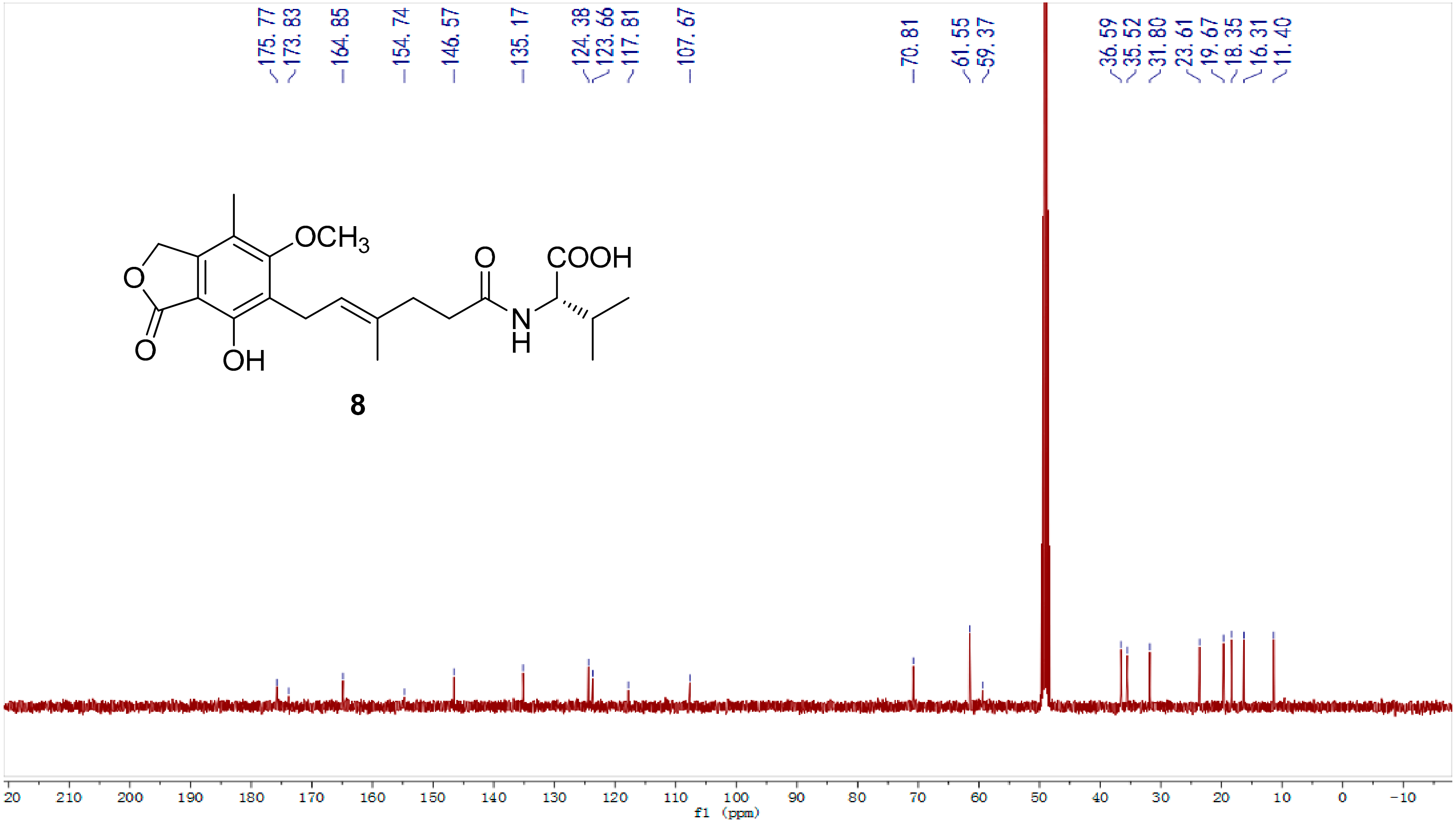

Figure S64. ^{13}C NMR spectrum of compound **8** (Recorded in CD_3OD)

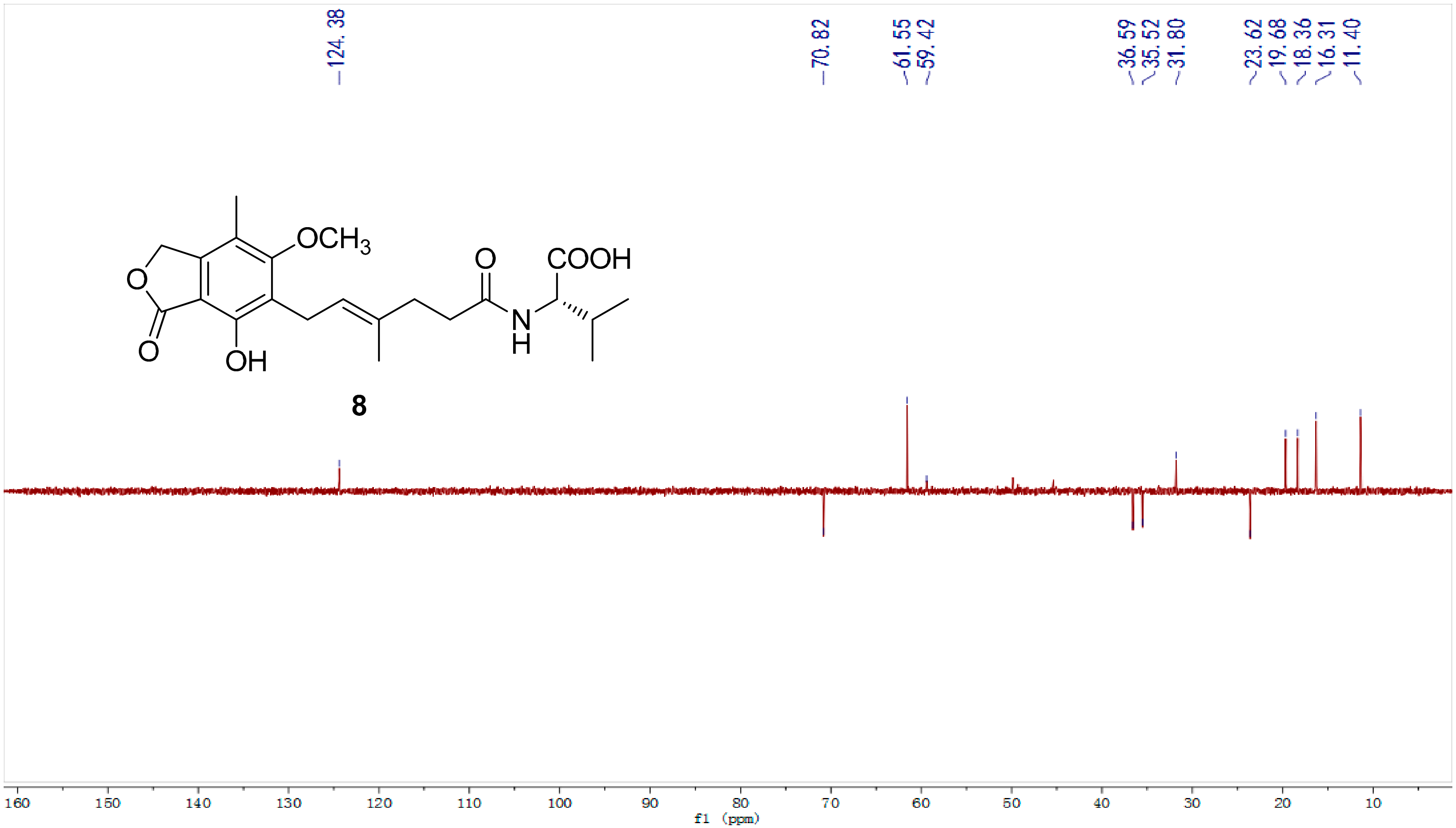

Figure S65. DEPT spectrum of compound **8** (Recorded in CD_3OD)

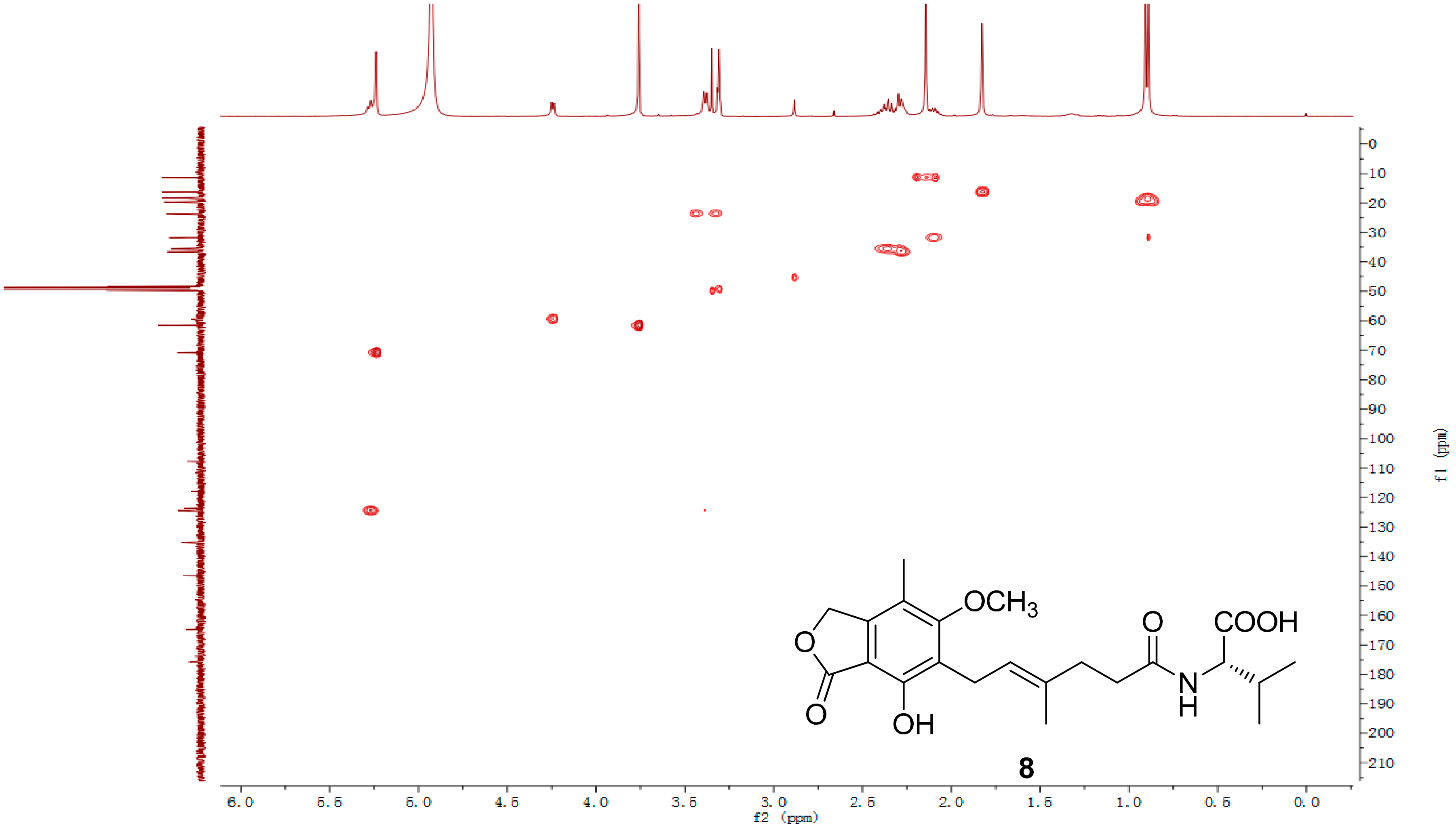

Figure S66. HSQC spectrum of compound **8** (Recorded in CD_3OD)

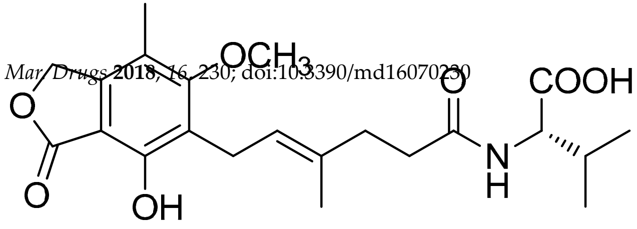

8

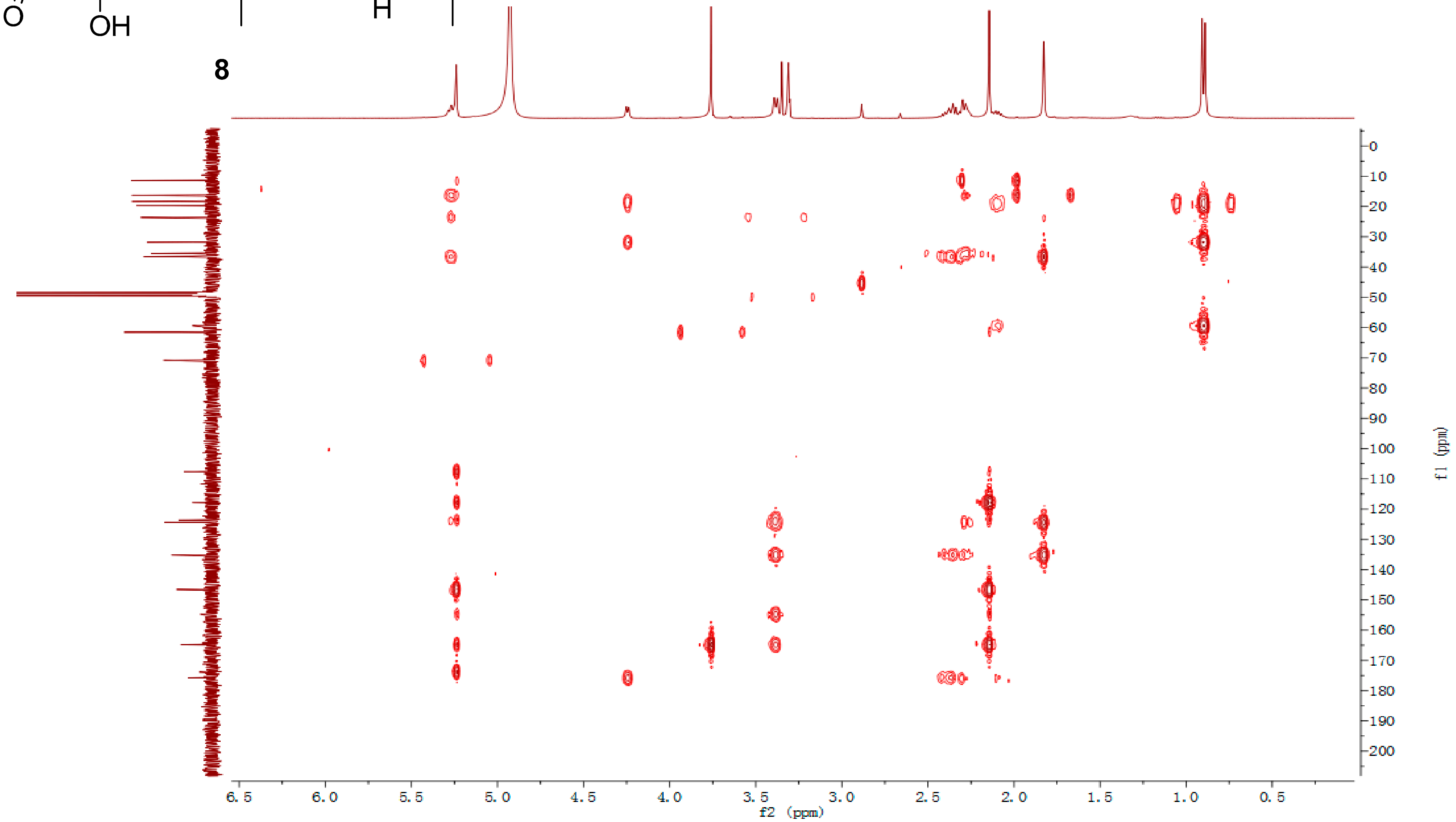

Figure S67. HMBC spectrum of compound **8** (Recorded in CD_3OD)

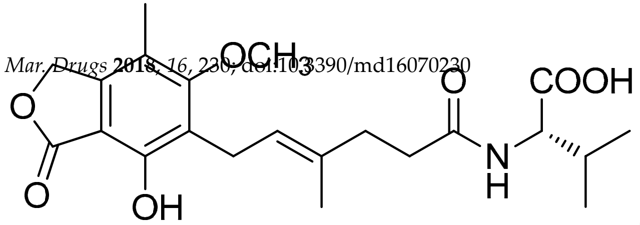

8

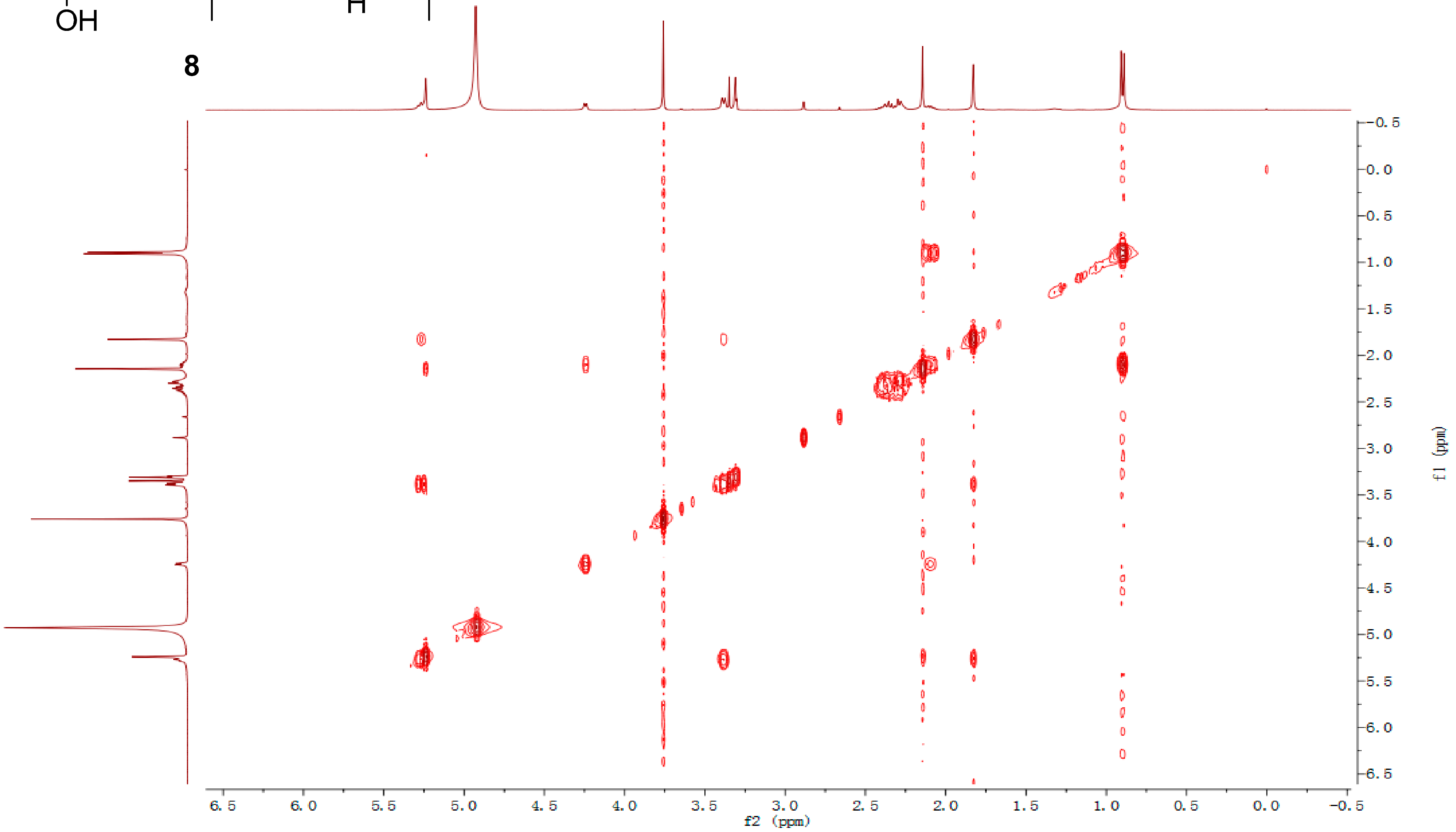

Figure S68. ^{1}H–^{1}H COSY spectrum of compound **8** (Recorded in CD_3OD)

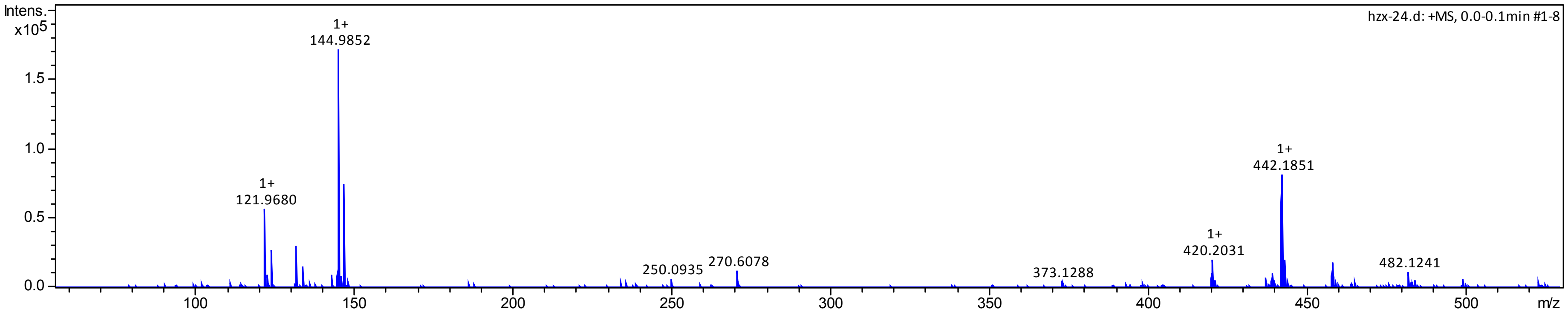

Figure S69. HRESIMS spectrum of compound **8**

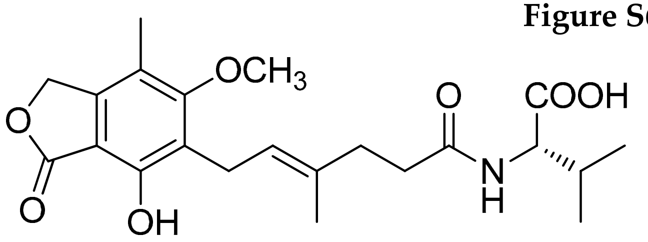

8

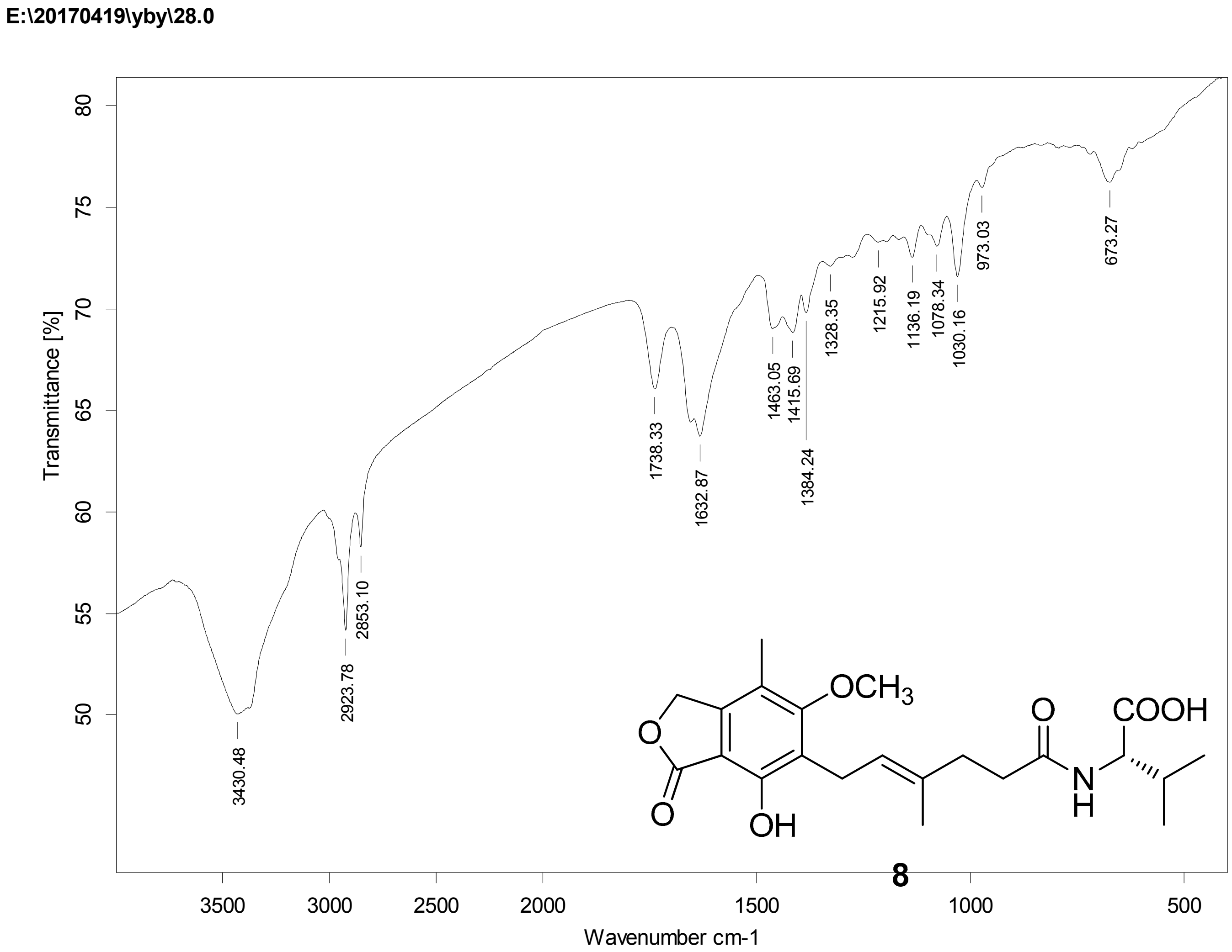

Figure S70. IR spectrum of compound **8**

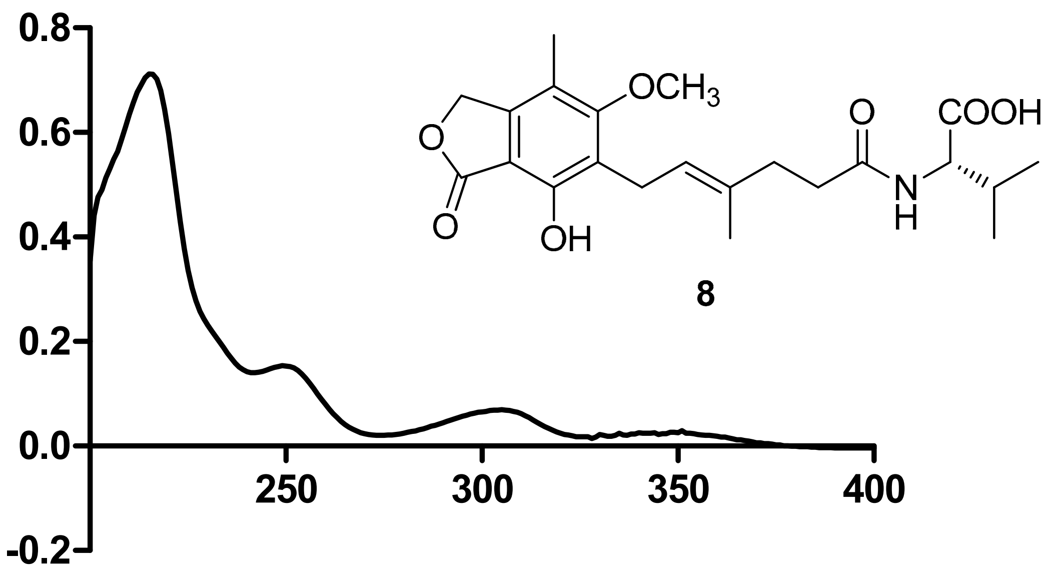

Figure S71. UV spectrum of compound **8**

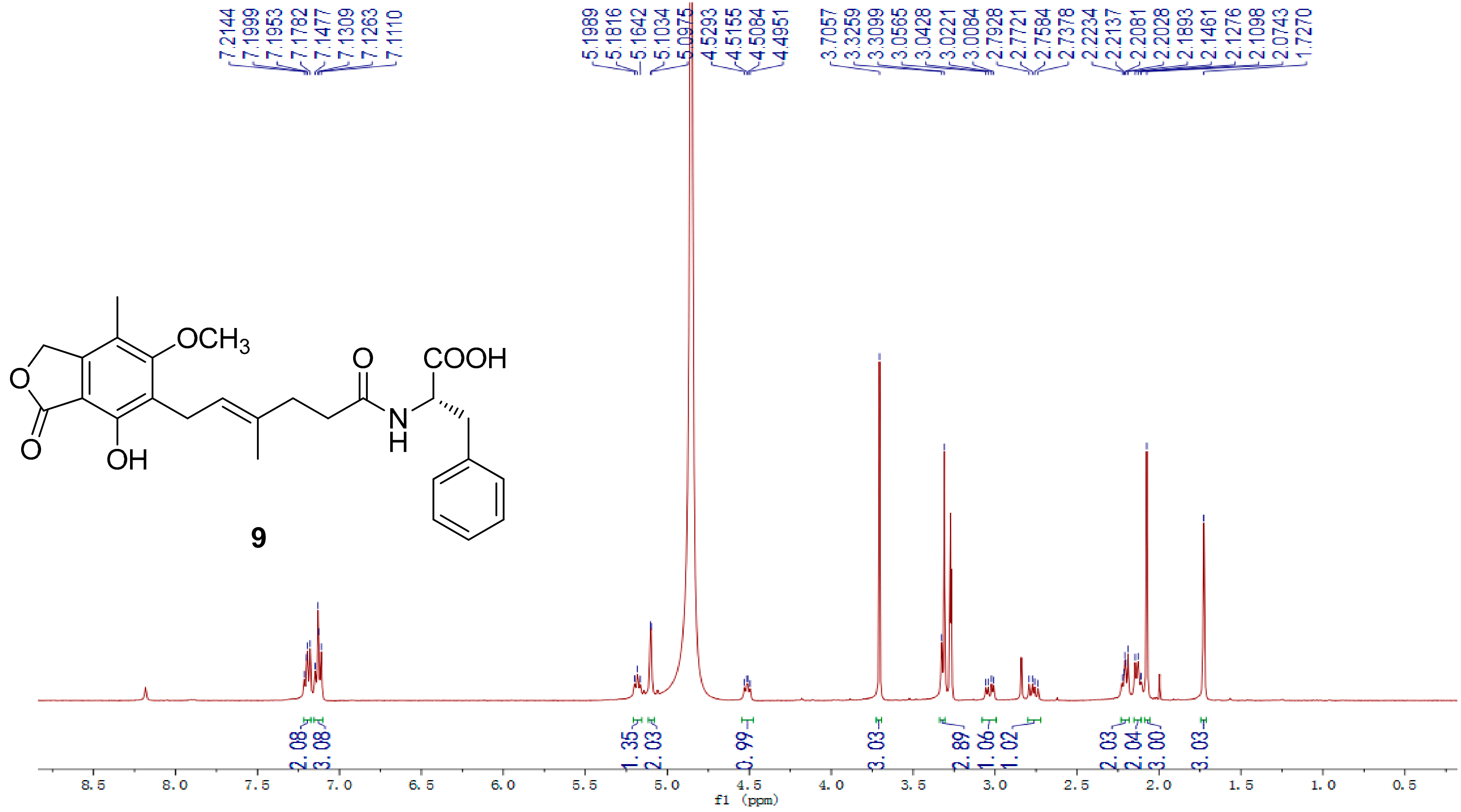

Figure S72. ^{1}H NMR spectrum of compound **9** (Recorded in CD_3OD)

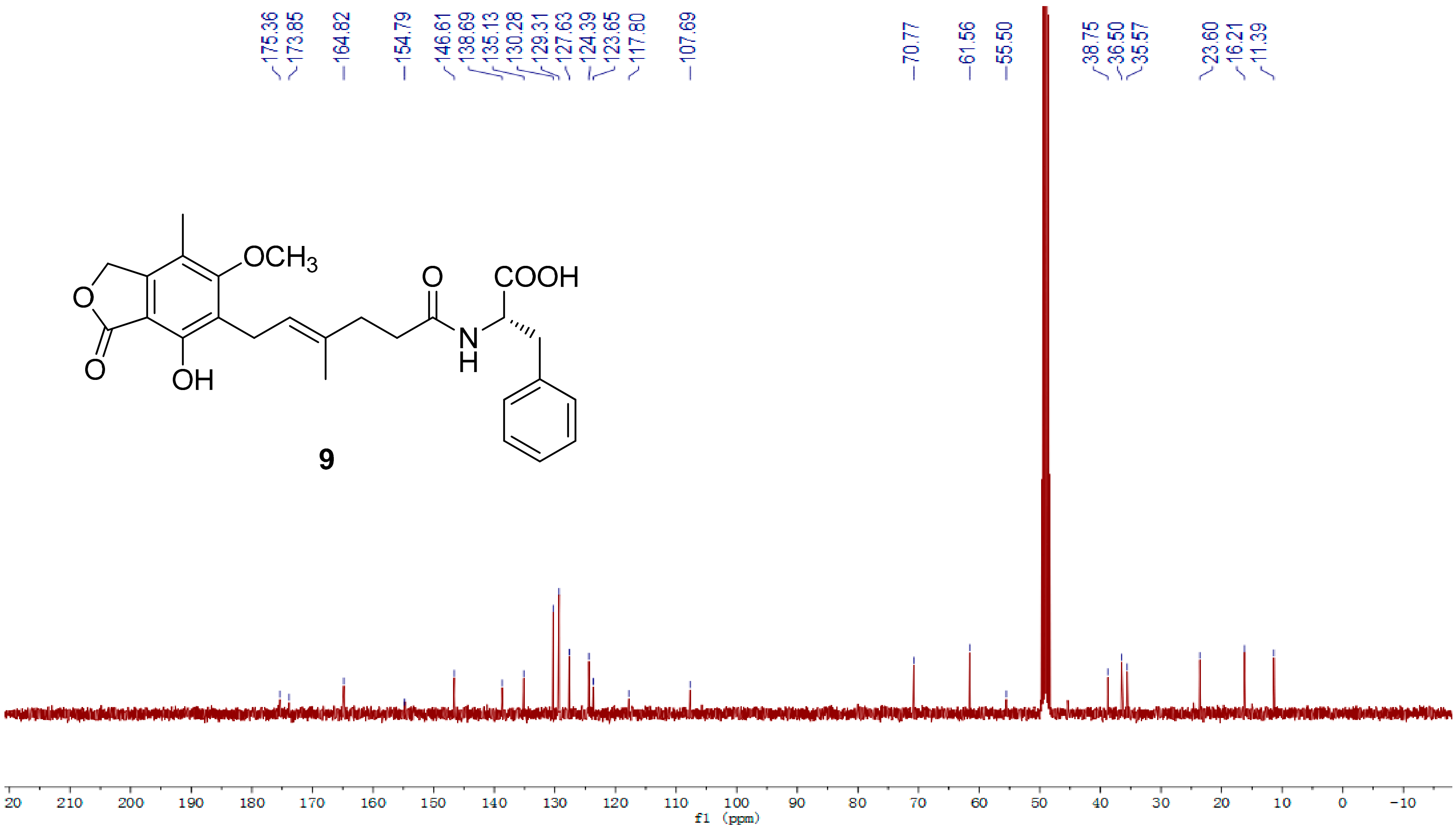

Figure S73. ^{13}C NMR spectrum of compound **9** (Recorded in CD_3OD)

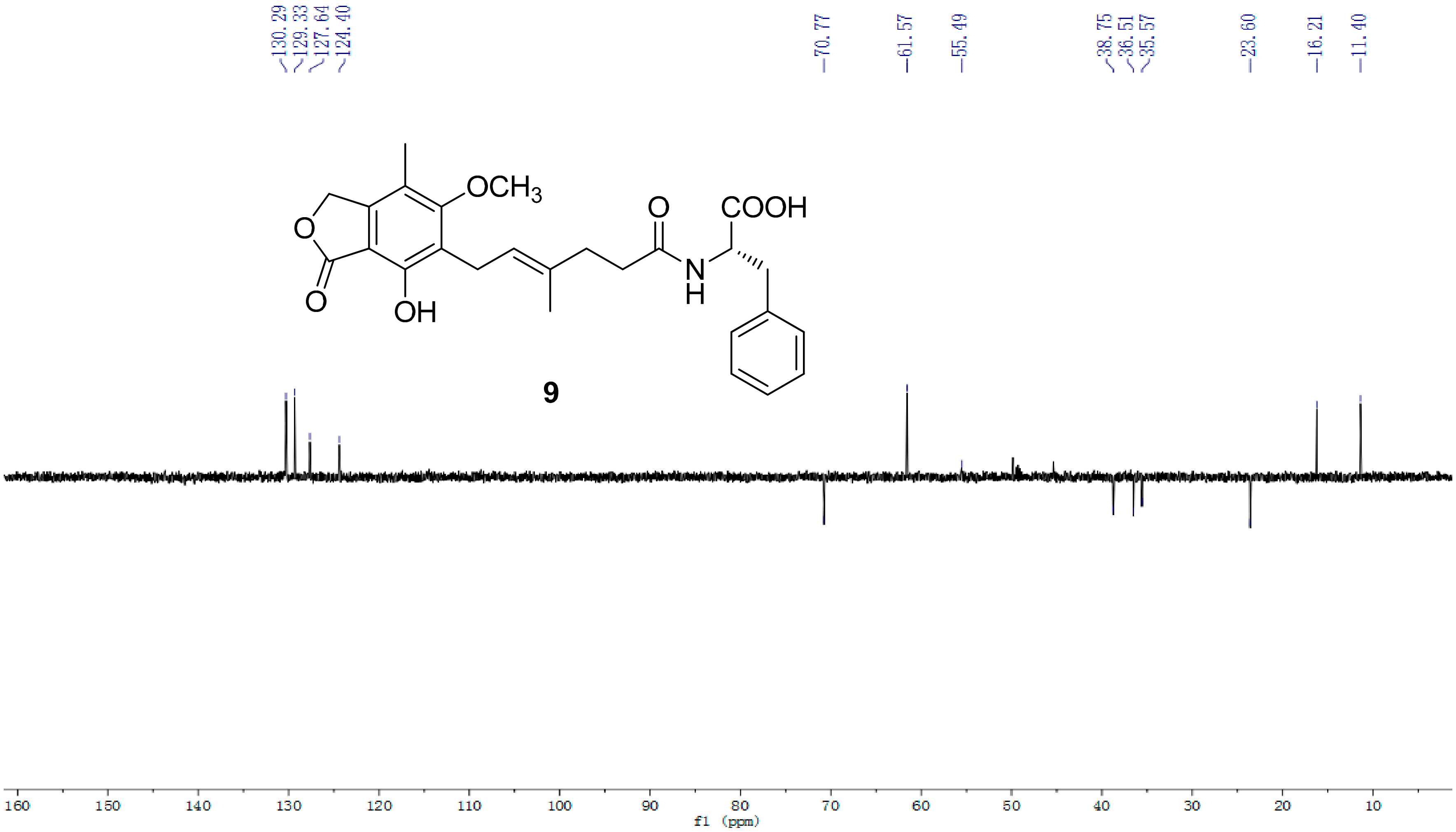

Figure S74. DEPT spectrum of compound **9** (Recorded in CD_3OD)

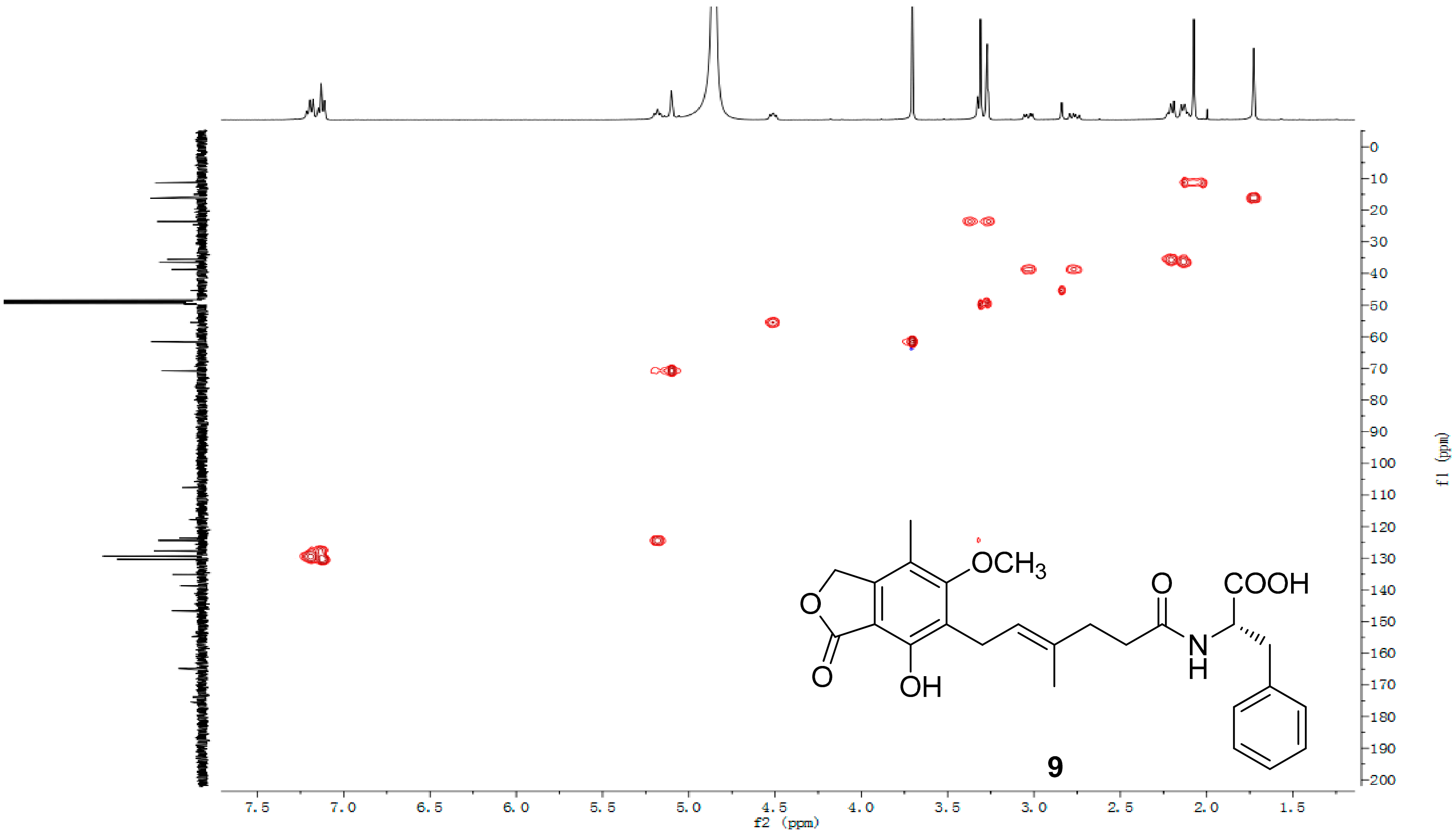

Figure S75. HSQC spectrum of compound **9** (Recorded in CD_3OD)

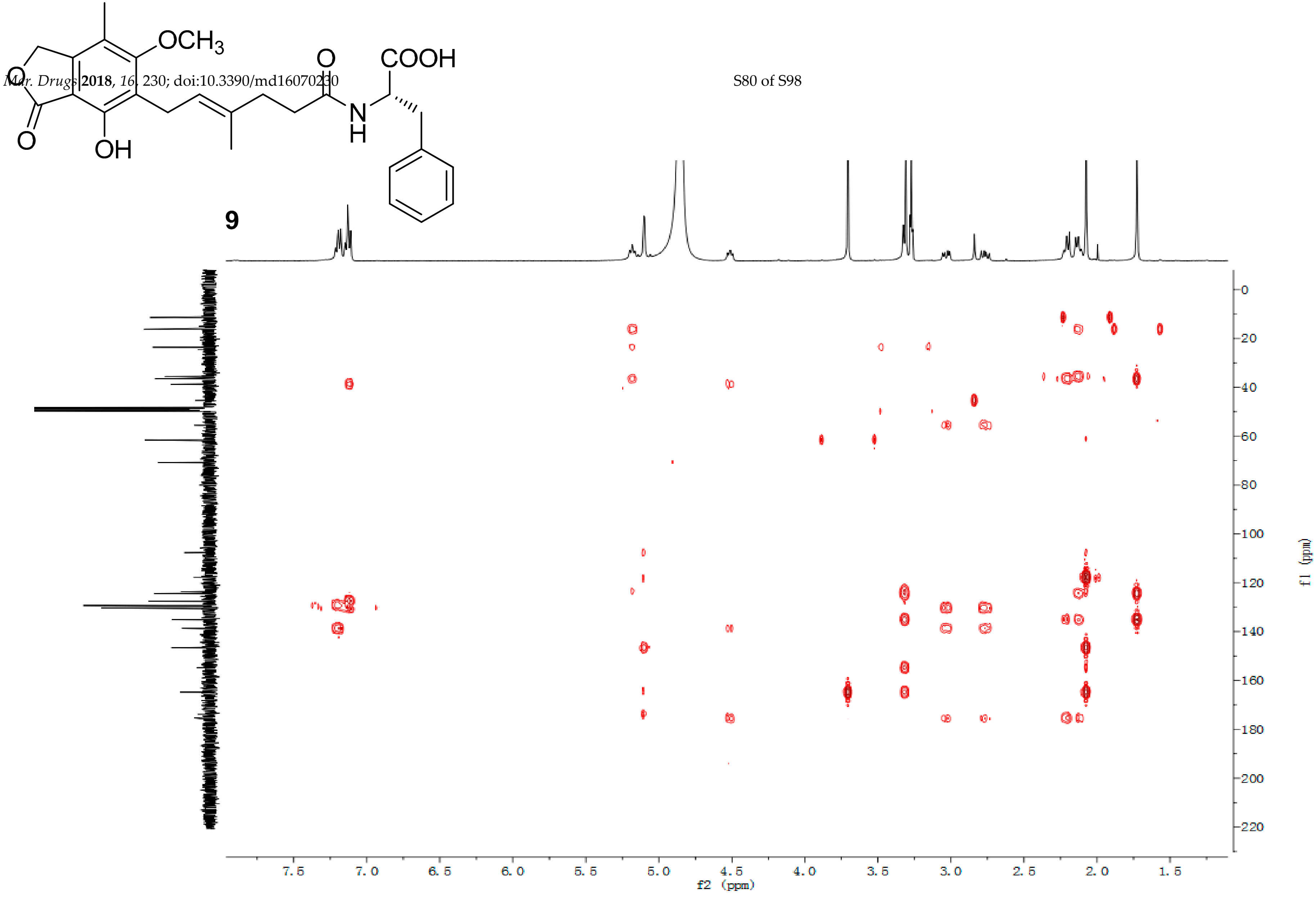

Figure S76. HMBC spectrum of compound **9** (Recorded in CD3OD)

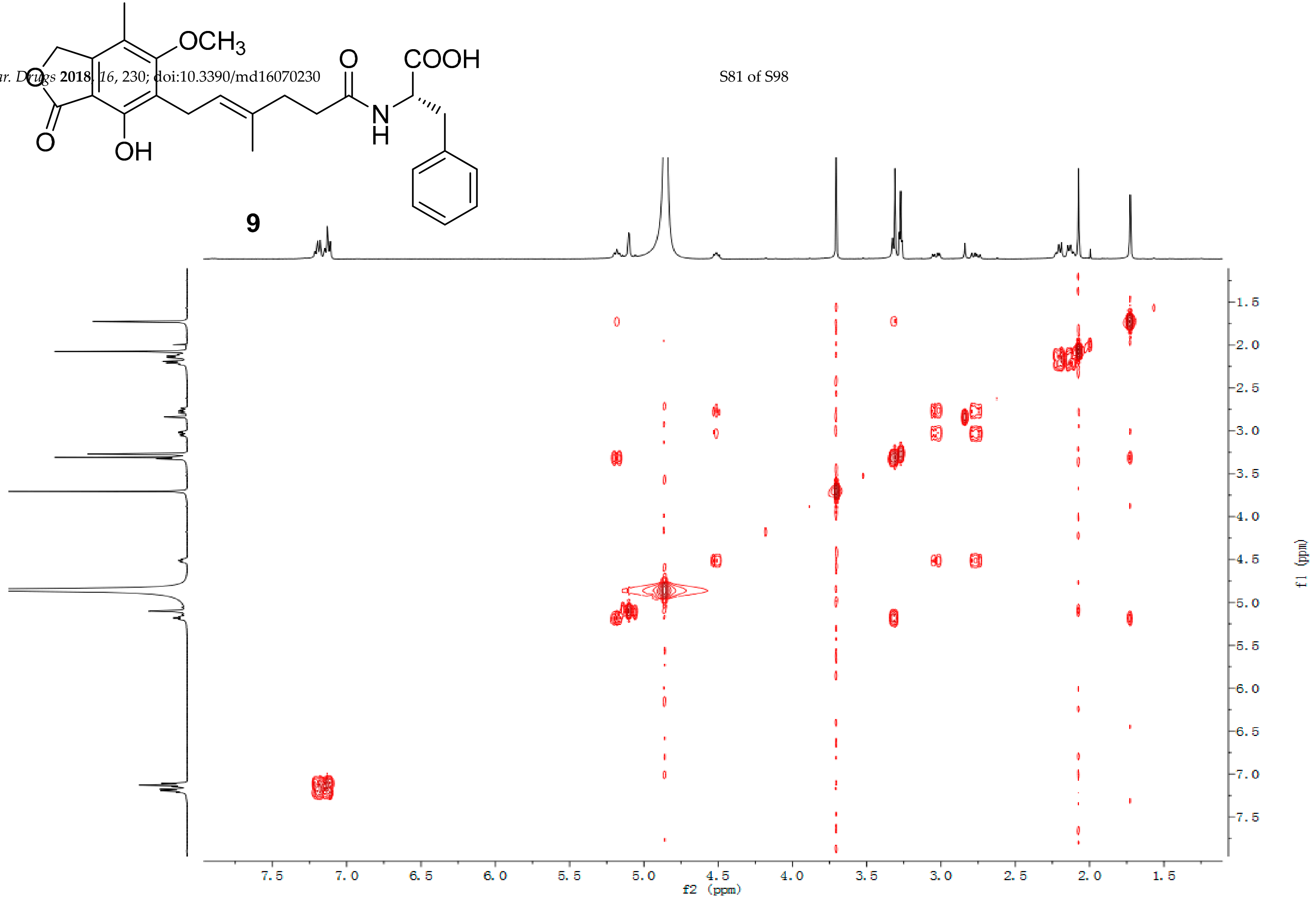

Figure S77. ^{1}H–^{1}H COSY spectrum of compound **9** (Recorded in CD3OD)

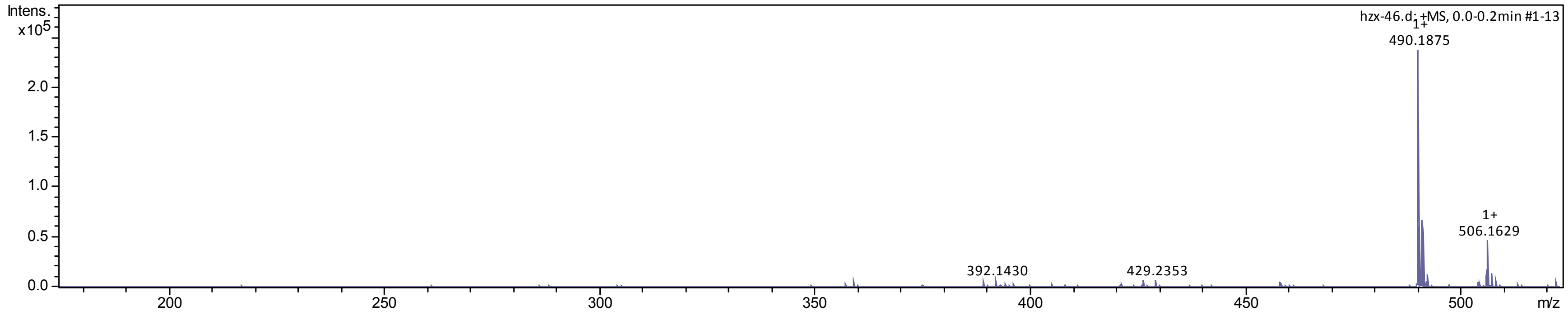

Figure S78. HRESIMS spectrum of compound **9**

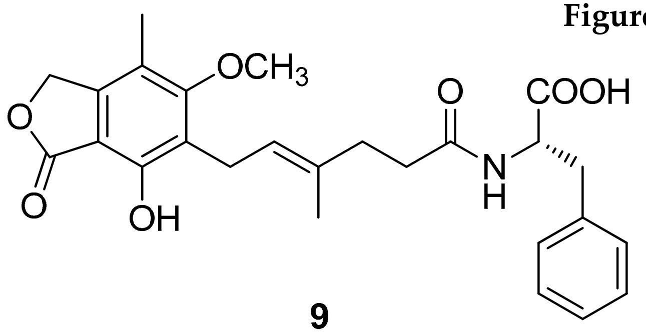

9

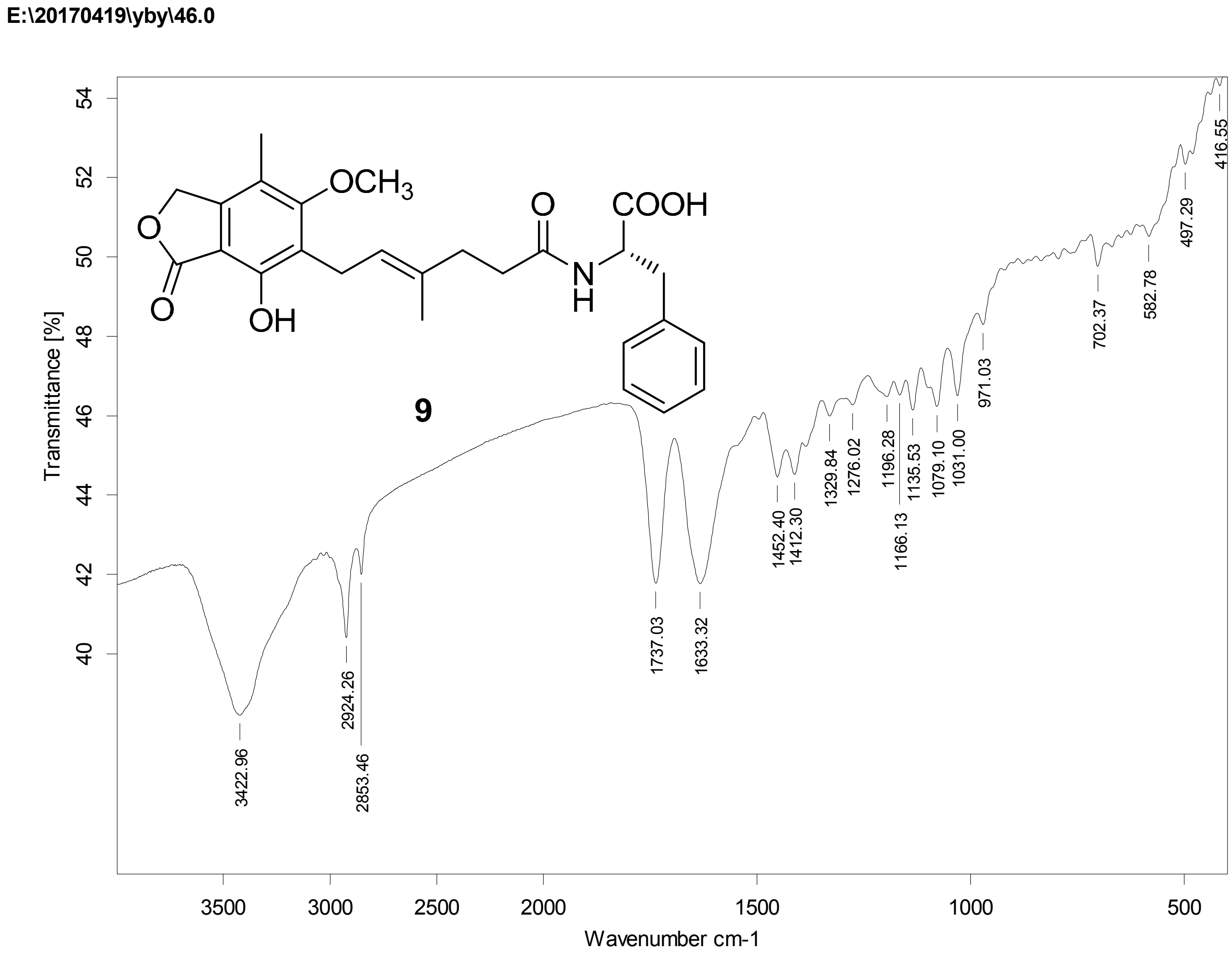

Figure S79. IR spectrum of compound **9**

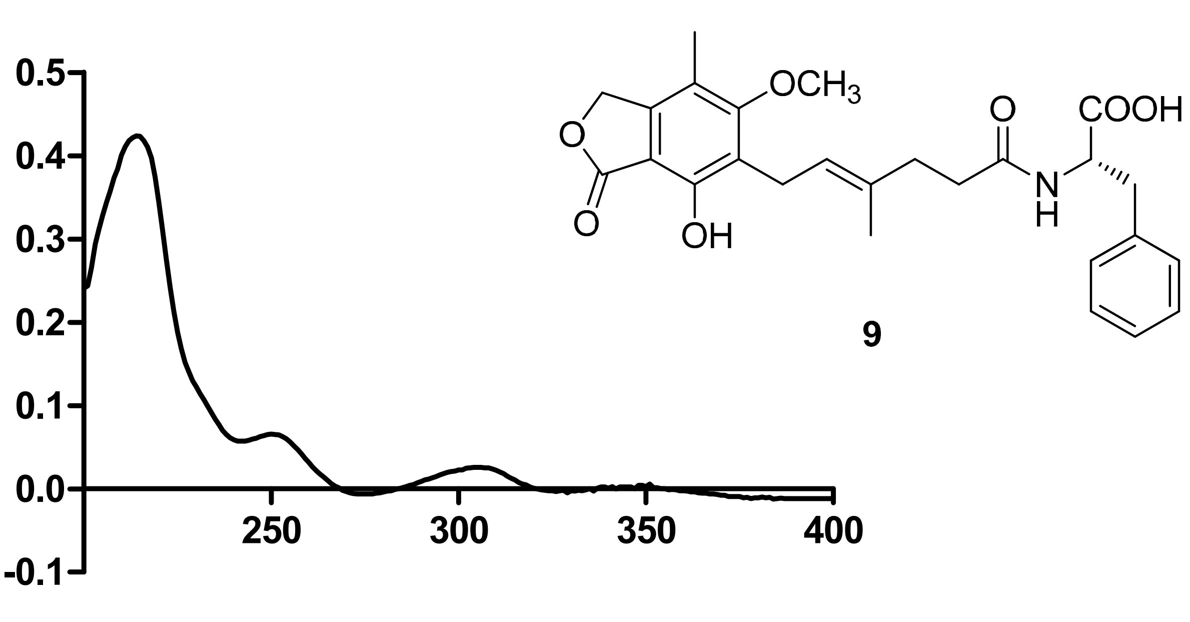

Figure S80. UV spectrum of compound **9**

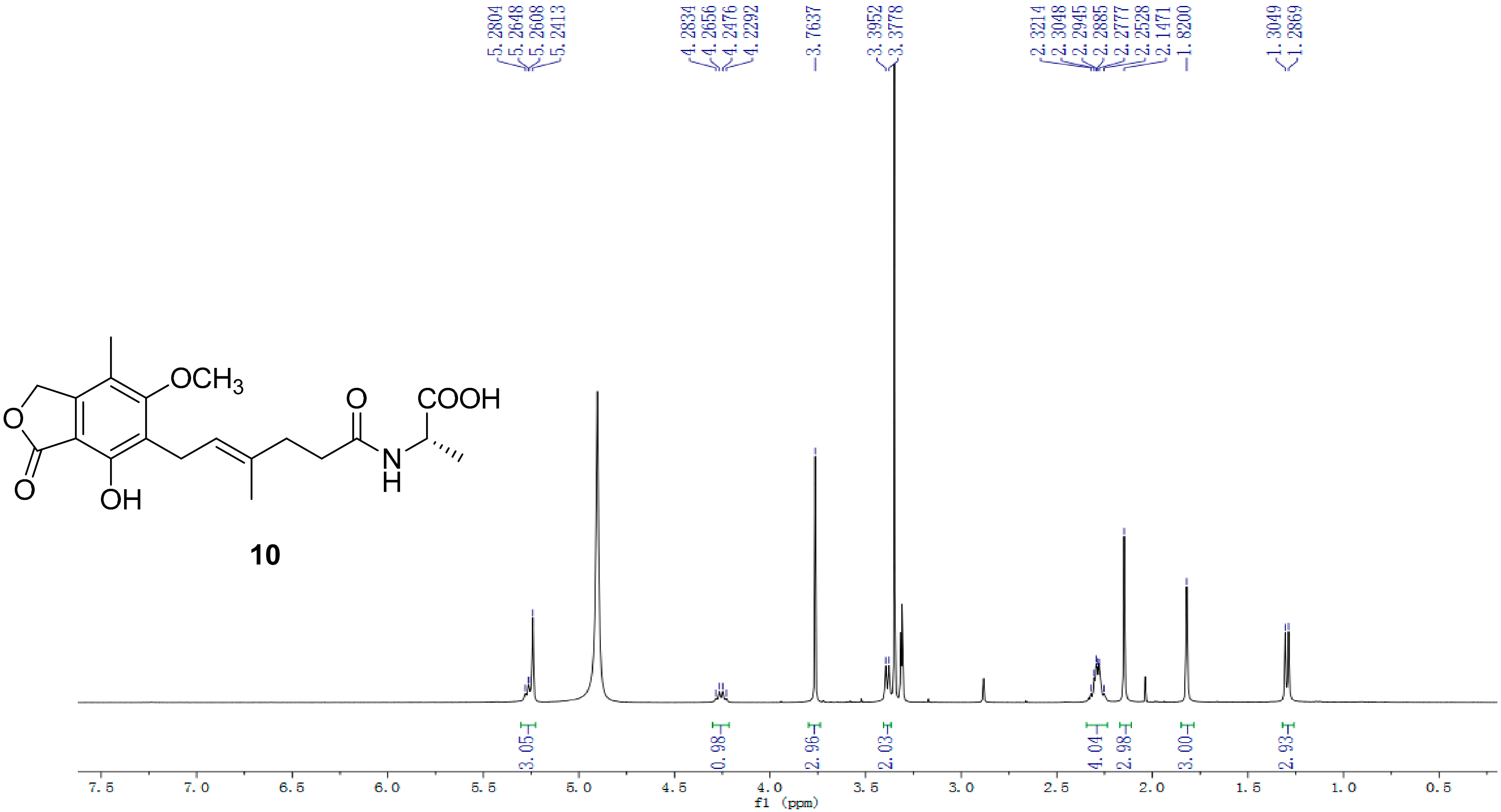

Figure S81. ^{1}H NMR spectrum of compound **10** (Recorded in CD_3OD)

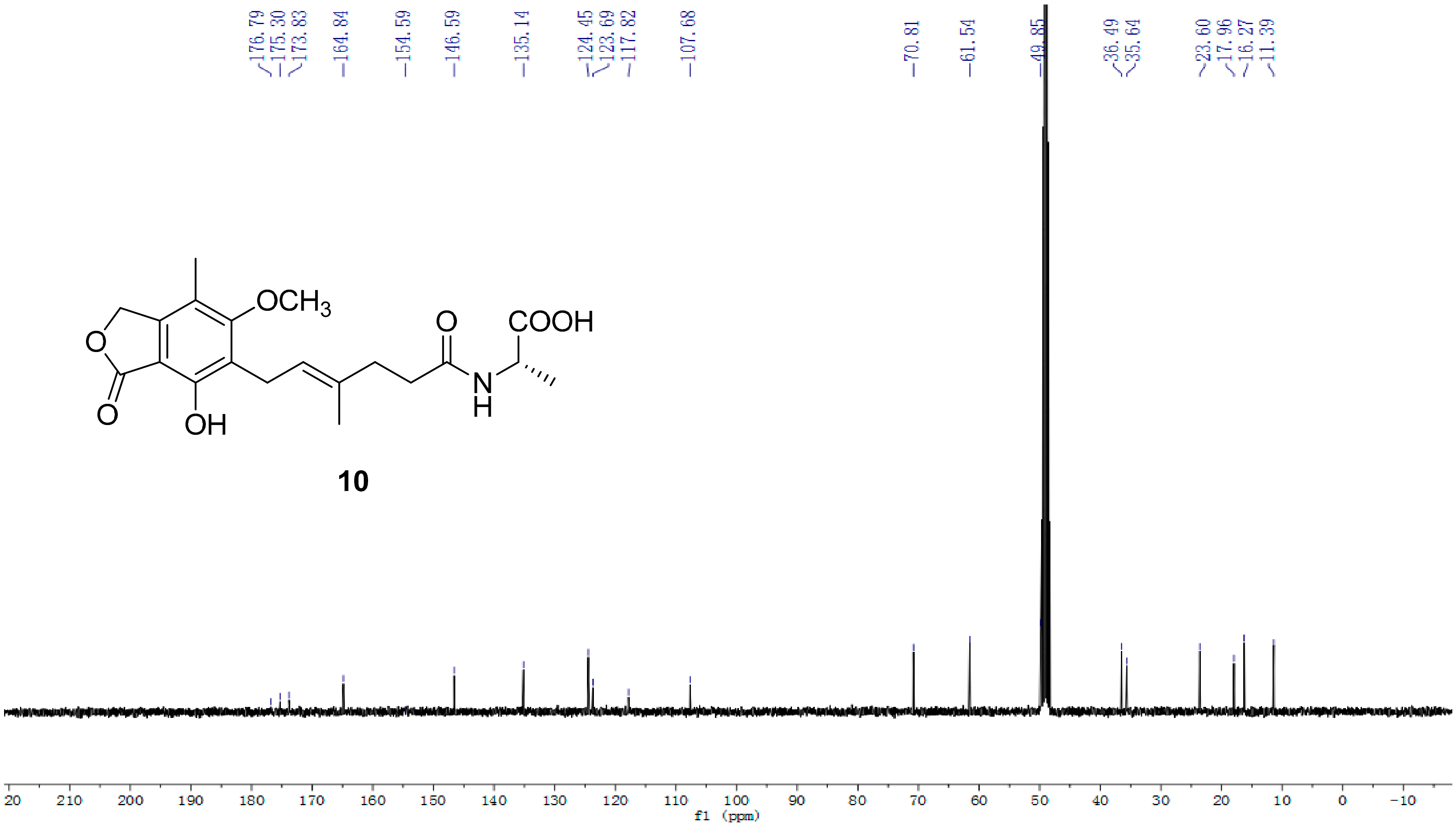

Figure S82. ^{13}C NMR spectrum of compound **10** (Recorded in CD_3OD)

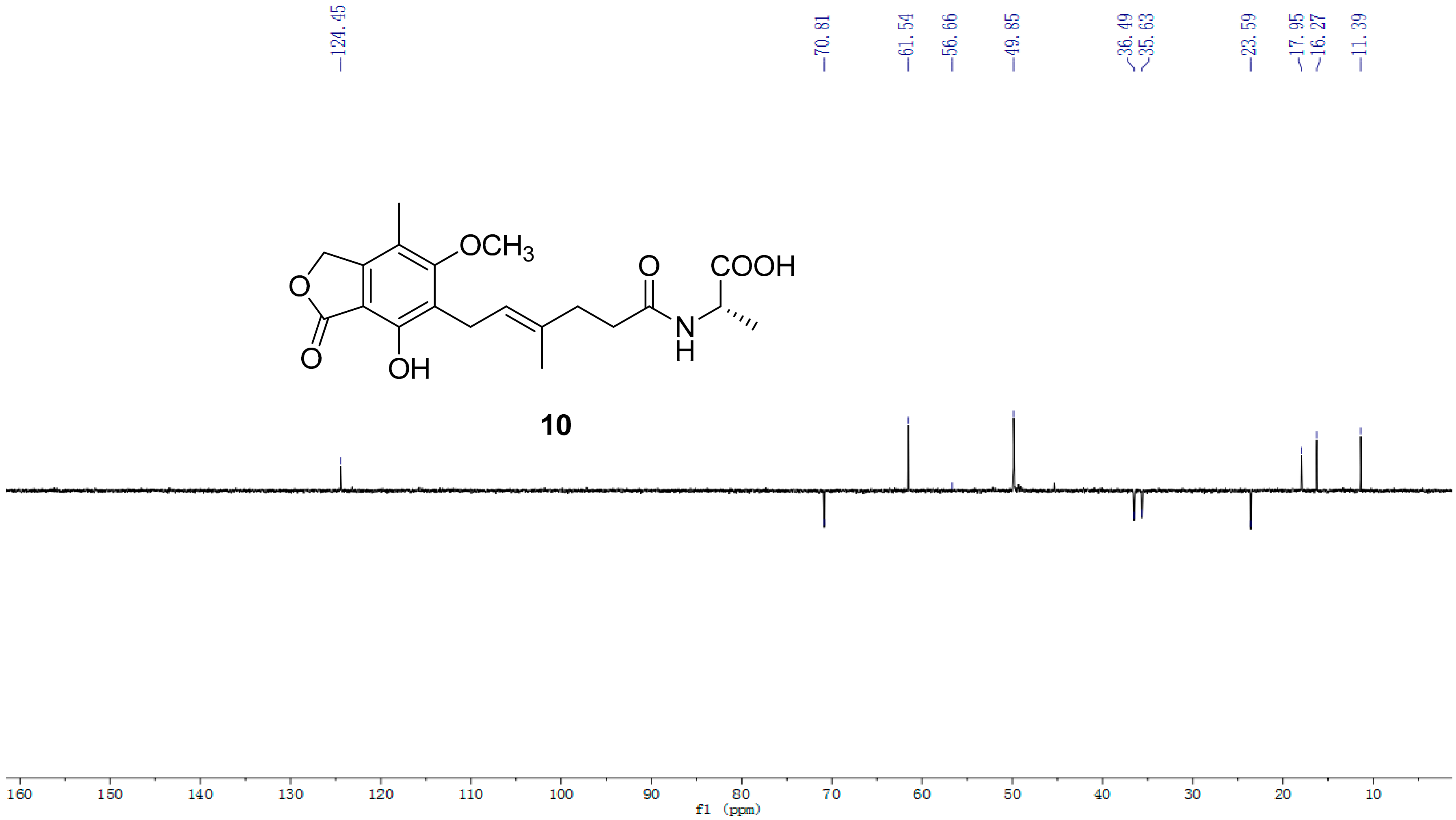

Figure S83. DEPT spectrum of compound **10** (Recorded in CD_3OD)

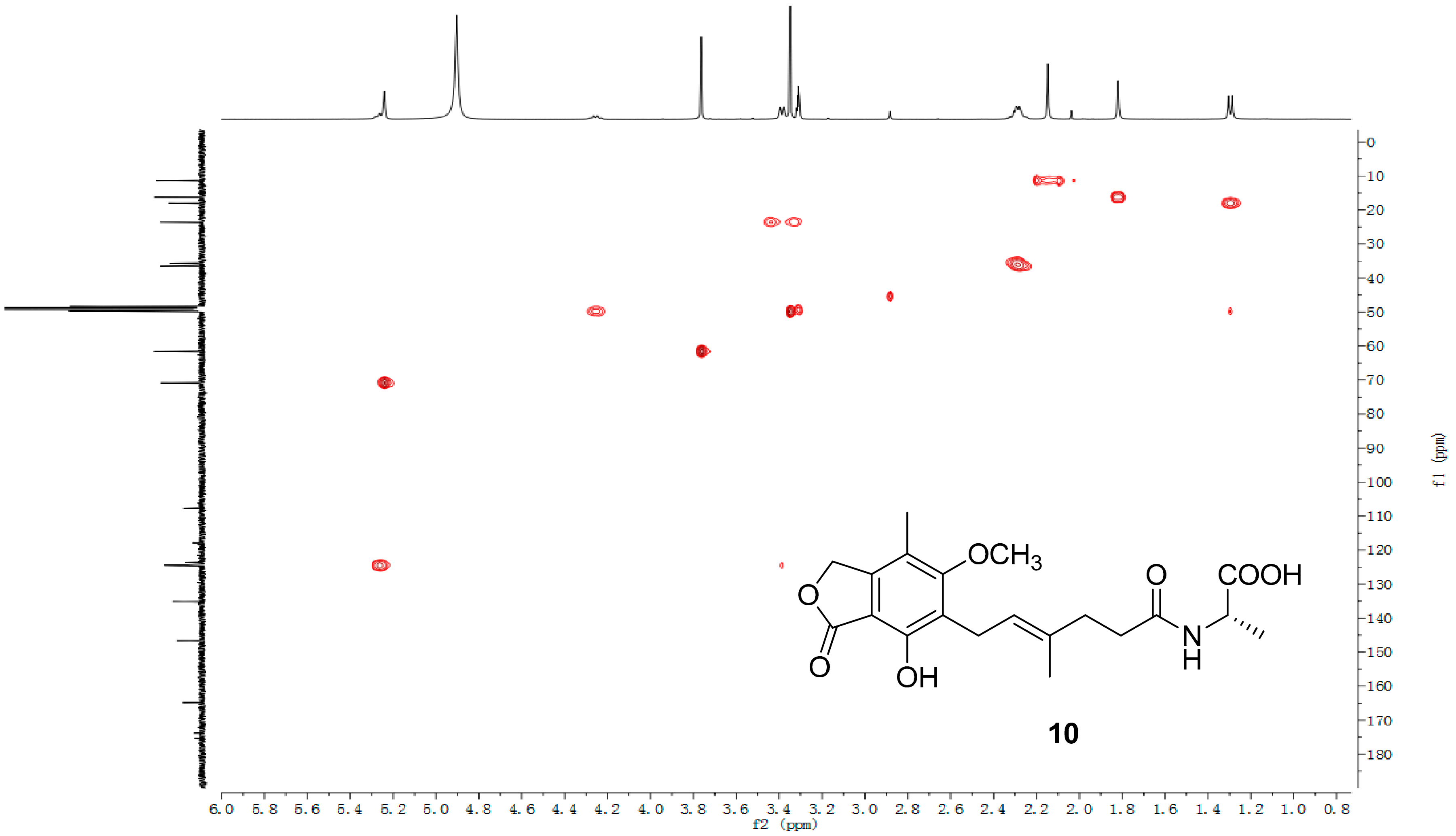

Figure S84. HSQC spectrum of compound **10** (Recorded in CD3OD)

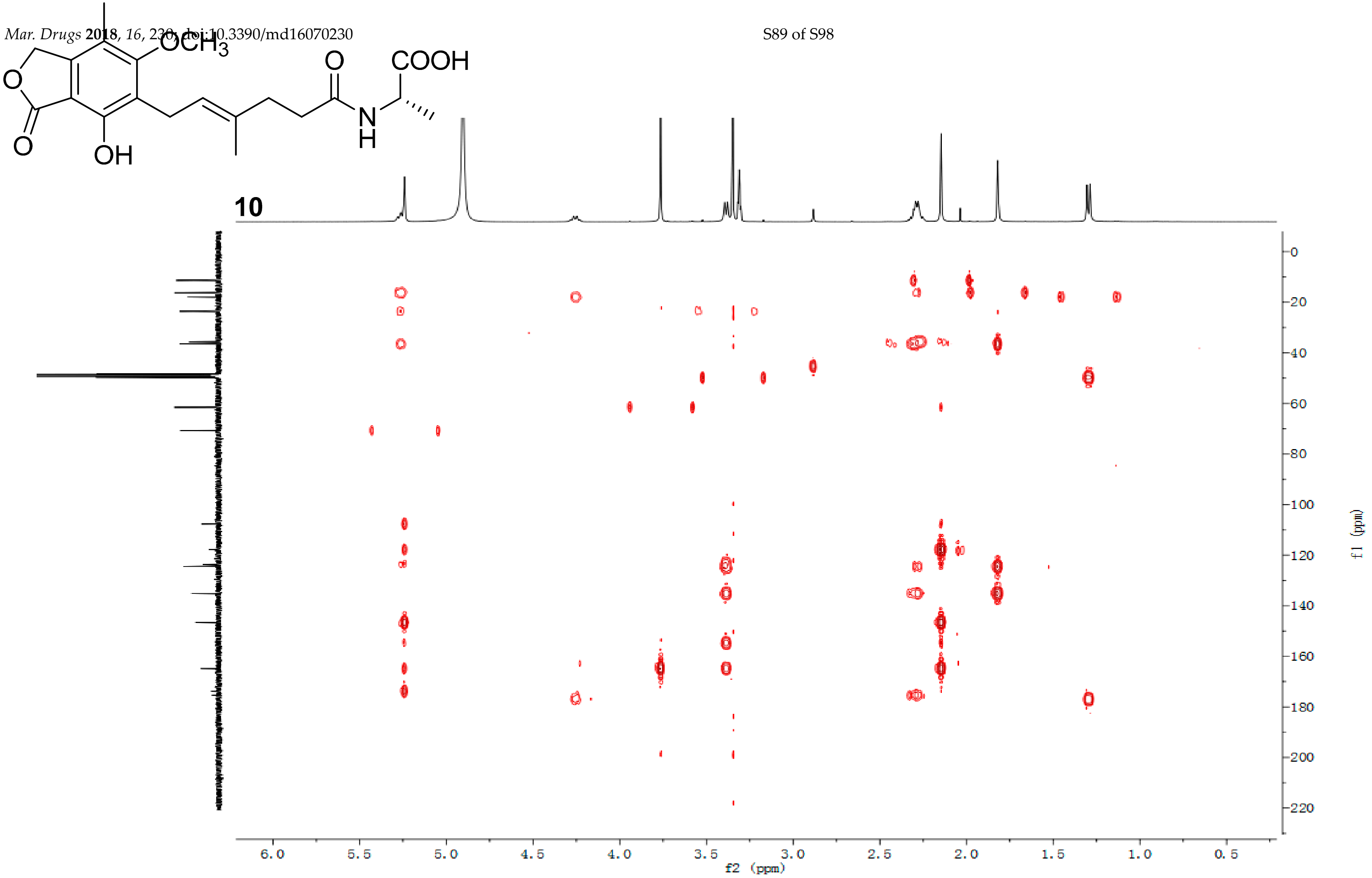

Figure S85. HMBC spectrum of compound **10** (Recorded in CD_3OD)

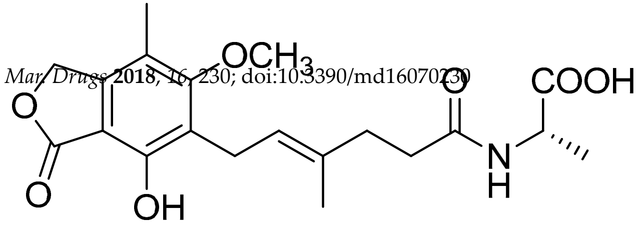

10

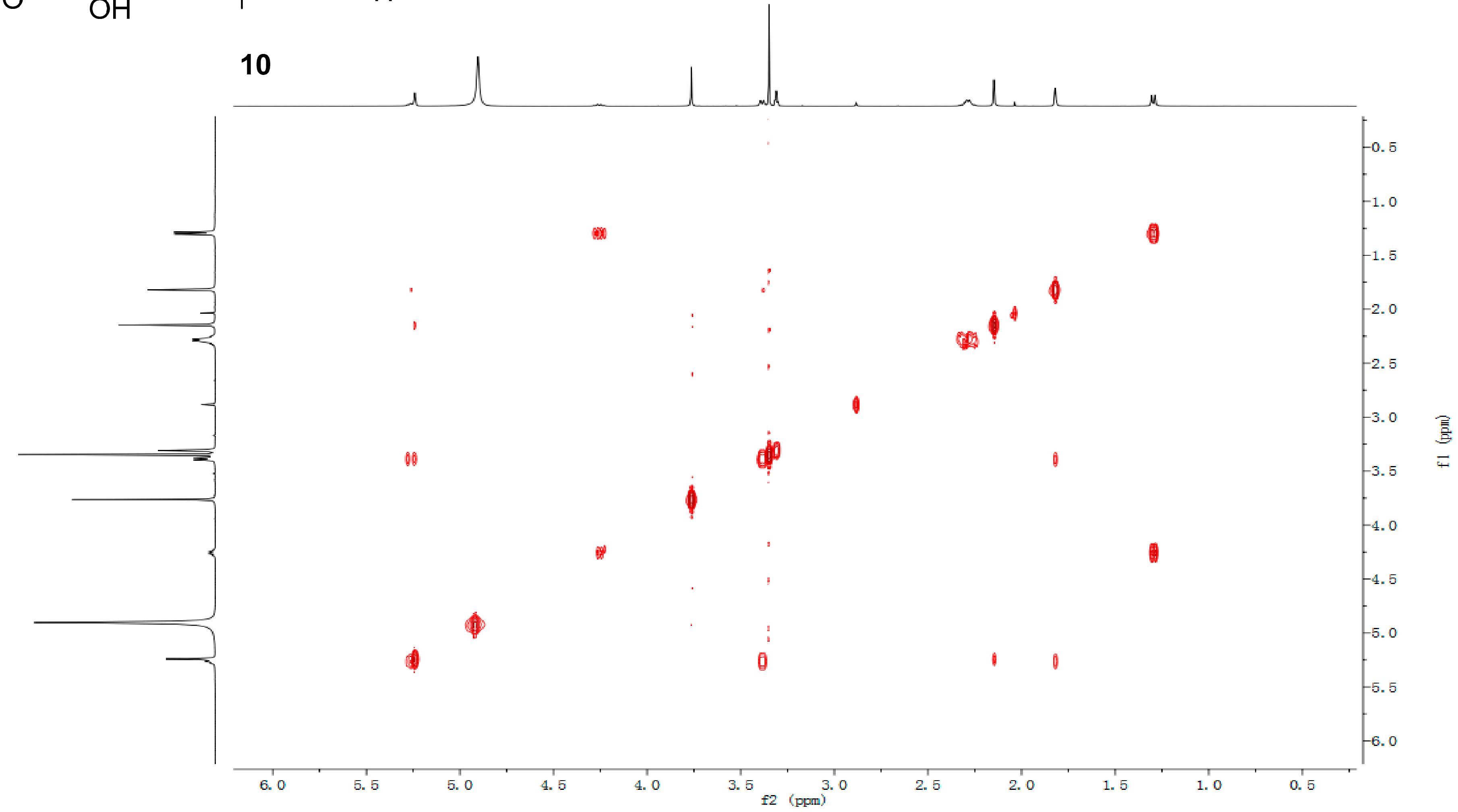

Figure S86. ^1H–^1H COSY spectrum of compound **10** (Recorded in CD_3OD)

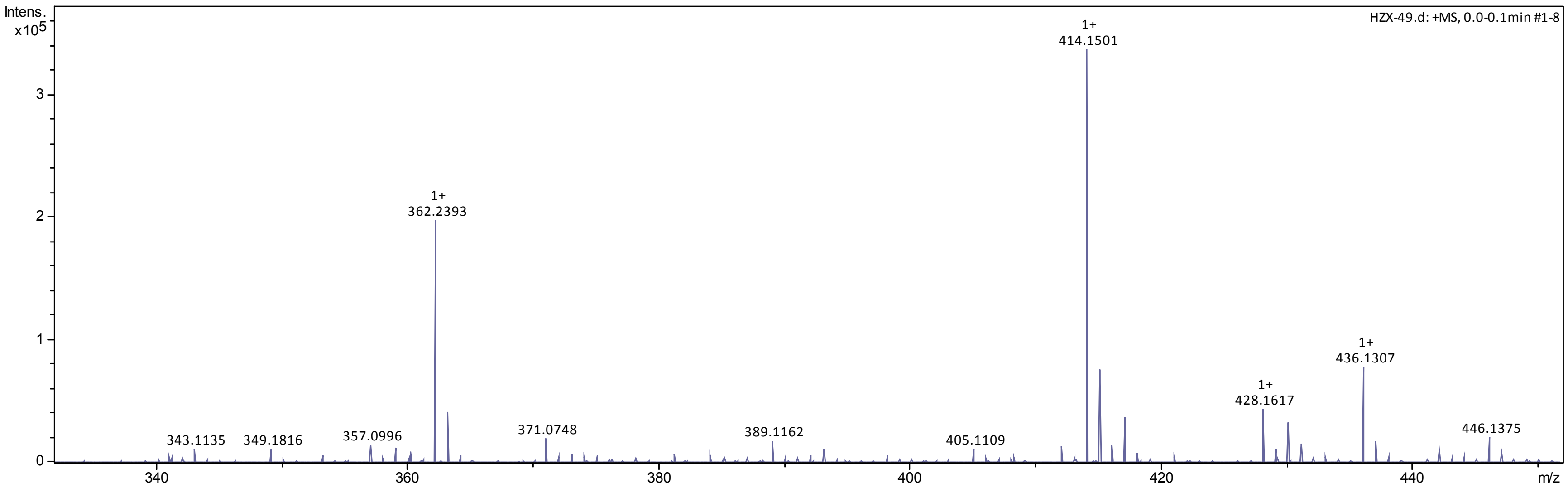

Figure S87. HRESIMS spectrum of compound **10**

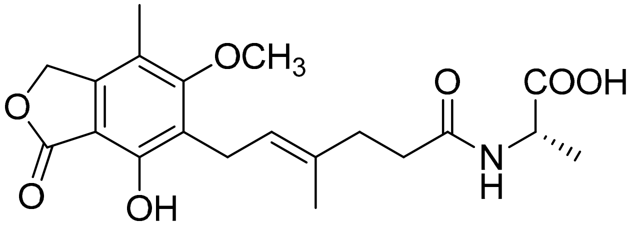

10

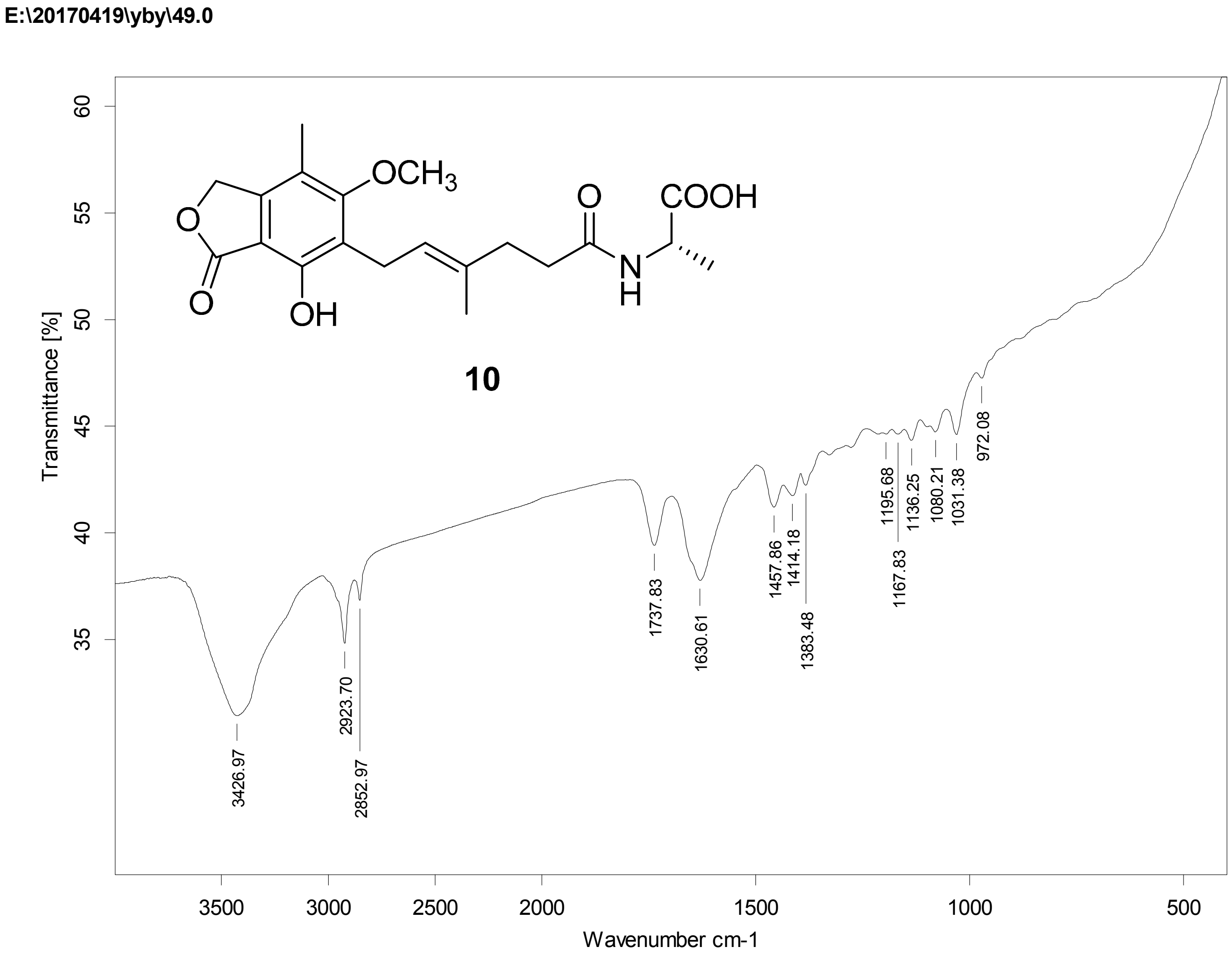

Figure S88. IR spectrum of compound **10**

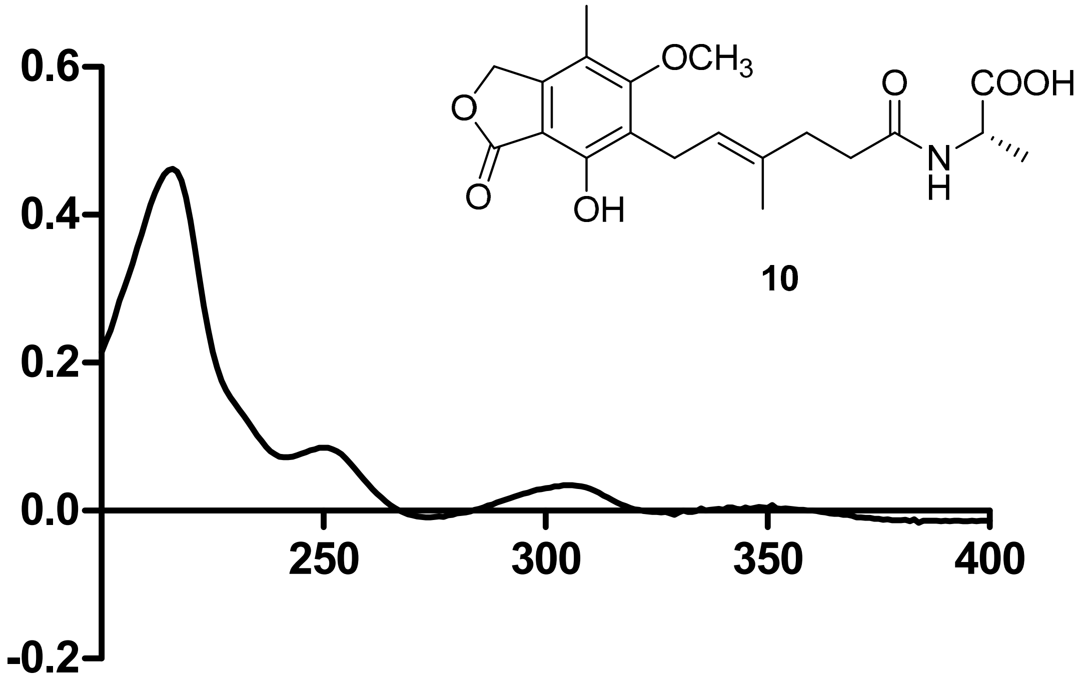

Figure S89. UV spectrum of compound **10**

Structure elucidation of compounds 1, 2, and 8–10

Compound **1** was isolated as a white powder, and its molecular formula was assigned as $C_{18}H_{22}O_7$, based on the HRESIMS data at *m*/*z* 373.1259 ([M + Na]$^+$, calcd for 373.1263) and ^{13}C NMR data, requiring eight indices of hydrogen deficiency. Detailed analysis of the ^{1}H and ^{13}C NMR data (Table S1) of **1** and **11** suggested that both compounds possessed the similar structural features, with the only difference that a methyl group at C-8 in **11** was replaced by a –CH_2OCH_3 group in **1**, as supported by the HMBC correlations from H_2-8 to C-3a (δ_C 147.2), C-4 (δ_C 119.1), C-5 (δ_C 164.5), and the methoxyl carbon (δ_C 58.7). Moreover, the planar structure was defined by the 2D NMR analysis, including HMBC and ^{1}H–^{1}H COSY correlations. Thus, the structure of **1** was identified and named 8-*O*-methyl mycophenolic acid.

Compound **2** was also isolated as a white powder. Its molecular formula $C_{17}H_{20}O_7$ was determined by the HRESIMS *m*/*z* 359.1092 [M + Na]$^+$ (calcd for $C_{17}H_{20}O_7Na$, 359.1107). The ^{1}H and ^{13}C NMR data (Table S1) showed high similarity to those of **11**, differing in that the C-3 methylene in **11** was hydroxylated in **2**, as verified by its molecular formula and the chemical shift values of CH-3 (δ_H 6.58; δ_C 100.2). This conclusion was further confirmed by the HMBC correlations from H-3 to C-1 (δ_C 171.6), C-3a (δ_C 145.7), C-4 (δ_C 120.3), and C-7a (δ_C 108.7). Compound **2** was optically inactive and no apparent Cotton effects were observed in its experimental CD spectrum despite the presence of an *α*,*β*-unsaturated carbonyl chromophore, suggesting that it was a racemic mixture. Moreover, an attempt to separate the two enantiomers was failed. Thus, the structure of **2** was defined and named 3-hydroxy mycophenolic acid.

Compound **8** was determined to have the molecular formula $C_{22}H_{29}NO_7$, according to its HRESIMS *m*/*z* 442.1851 [M + Na]$^+$ (calcd for $C_{22}H_{29}NO_7Na$, 442.1842). The ^{1}H and ^{13}C NMR data (Table S2) were similar to those of **11**, except for the presence the valine residue which formed an amide bond with C-6', as supported by the COSY correlations from H-2'' (δ_H 4.24) to H-4'' (δ_H 2.10) and from H-4'' to H-5'' (δ_H 0.90) and H-6'' (δ_H 0.90) and HMBC correlations from H_2-2'' to C-6' and C-3'' and from H-4'' to C-3''. The specific rotation of **8** {$[\alpha]_D^{23}$: +5.3 (*c* 0.08, MeOH)} was dextrorotatory, the same to that of the synthetic product *N*-mycophenoyl-L-valine {$[\alpha]_D^{25}$: +2.0 (*c* 1, acetone)} [1], suggesting the presence of a *L*-valine in **8**. Thus, the structure of **8** was identified as *N*-mycophenoyl-L-valine.

Compounds **9** and **10** were also identified as the natural mycophenolic acid-amino acid conjugates. And their molecular formulas were determined to be $C_{26}H_{29}NO_7$ and $C_{20}H_{25}NO_7$, respectively, based on the HRESIMS analysis as well as ^{13}C NMR data. Detailed analysis of the 1D and 2D NMR data (Table S2) of **9** and **10** with those of **8** revealed the obvious differences that **9** had a phenylalanine amide unit and **10** had an alanine amide unit, as supported by the key 2D NMR analysis. Compound **9** was measured with a specific rotation, $[\alpha]_D^{23}$: +2.5 (*c* 0.1, MeOH), which was identical to that of the synthetic product *N*-mycophenoyl-L-phenyloalanine {$[\alpha]_D^{25}$: +2.0 (*c* 1, MeOH)} [1], suggesting the presence of a *L*-phenylalanine amide unit

in **9**. Similarly, comparing the specific rotation of **10** {$[\alpha]_D^{23}$: –6.4 (*c* 0.08, MeOH)} with that of the synthetic product *N*-mycophenoyl-L-alanine {$[\alpha]_D^{25}$: –2.0 (*c* 1, acetone)} [1], the similar negative values indicated the presence of a L-alanine amide unit in **10**. Thus, the structures of **9** and **10** were defined and named *N*-mycophenoyl-L-phenyloalanine and *N*-mycophenoyl-L-alanine, respectively.

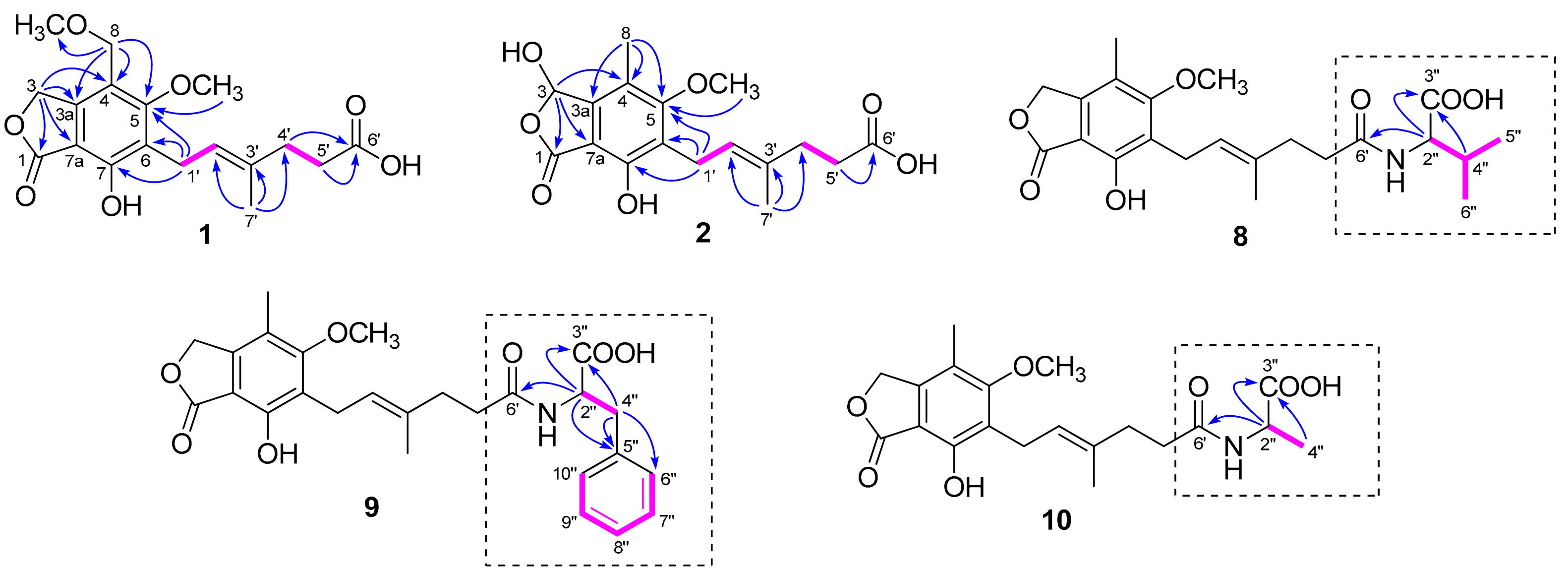

Figure S90. Key ^{1}H–^{1}H COSY (pink lines) and HMBC (blue arrows) correlations of **1**–**2** and **8**–**10**.

Ref: [1] Iwaszkiewicz-Grzes, D.; Cholewinski, G.; Kot-Wasik, A.; Trzonkowski, P.; Dzierzbicka, K. Synthesis and biological activity of mycophenolic acid-amino acid derivatives. *Eur. J. Med. Chem.* **2013**, *69*, 863–871.

The physical and chemical constants of compounds 1, 2, and 8–10

8-*O*-Methyl mycophenolic acid (**1**): white powder; UV (MeOH) λ_{max} (log ε): 219 (4.57), 253 (3.81), and 305 (3.51) nm; IR (ν_{max}): 3424, 2924, 2853, 1738, 1618, 1453, 1421, 1381, 1273, 1109, 1028, 969, 674 cm^{-1}; HRESIMS *m/z* 373.1259 [M + Na]$^+$ (calcd for $C_{18}H_{22}O_7Na$, 373.1263); ^{1}H and ^{13}C NMR data, see Table S1.

3-Hydroxy mycophenolic acid (**2**): white powder; $[\alpha]^{23}_{D}$: 0 (*c* 0.15, MeOH); UV (MeOH) λ_{max} (log ε): 218 (4.41), 251 (3.73), and 311 (3.56) nm; IR (ν_{max}): 3436, 2947, 1737, 1626, 1460, 1408, 1276, 1138, 1092, 1024, 595 cm^{-1}; HRESIMS *m/z* 359.1092 [M + Na]$^+$ (calcd for $C_{17}H_{20}O_7Na$, 359.1107); ^{1}H and ^{13}C NMR data, see Table S1.

***N*-Mycophenoyl-L-valine** (**8**): white powder; $[\alpha]^{23}_{D}$: +5.3 (*c* 0.08, MeOH); UV (MeOH) λ_{max} (log ε): 217 (4.24), 251 (3.49), and 307 (3.16) nm; IR (ν_{max}): 3430, 2924, 2853, 1738, 1633, 1463, 1416, 1384, 1136, 1078, 1030, 973, 673 cm^{-1}; HRESIMS *m/z* 442.1851 [M + Na]$^+$ (calcd for $C_{22}H_{29}NO_7Na$, 442.1842); ^{1}H and ^{13}C NMR data, see Table S2.

***N*-Mycophenoyl-L-phenyloalanine** (**9**): white powder; $[\alpha]^{23}_{D}$: +2.5 (*c* 0.1, MeOH); UV (MeOH) λ_{max} (log ε): 215 (4.39), 253 (3.64), and 308 (3.17) nm; IR (ν_{max}): 3423, 2924, 2853, 1737, 1633, 1452, 1412, 1330, 1136, 1079, 1031, 971, 702, 583 cm^{-1}; HRESIMS *m/z* 490.1875 [M + Na]$^+$ (calcd for $C_{26}H_{29}NO_7Na$, 490.1842); ^{1}H and ^{13}C NMR data, see Table S2.

***N*-Mycophenoyl-L-alanine** (**10**): white powder, $[\alpha]^{23}_{D}$: –6.4 (*c* 0.08, MeOH); UV (MeOH) λ_{max} (log ε): 218 (4.29), 253 (3.58), and 307 (3.16) nm; IR (ν_{max}): 3427, 2924, 2853, 1738, 1631, 1458, 1414, 1383, 1136, 1080, 1031, 972 cm^{-1}; HRESIMS *m/z* 414.1501 [M + Na]$^+$ (calcd for $C_{20}H_{25}NO_7Na$, 414.1529); ^{1}H and ^{13}C NMR data, see Table S2.

^{1}H and ^{13}C NMR data for compounds 1, 2, and 8–10

Table S1. ^{1}H and ^{13}C NMR data for **1** and **2** in CD_3OD (δ in ppm, J in Hz).

No.	1		2	
	δ_H [a,b]	δ_C [c]	δ_H [a,b]	δ_C [c]
1	-	173.6 CO	-	171.6 CO
3	5.33 (2H, s)	71.4 CH_2	6.58 (1H, s)	100.2 CH
3a	-	147.2 C	-	145.7 C
4	-	119.1 C	-	120.3 C
5	-	164.5 C	-	165.2 C
6	-	123.8 C	-	125.5 C
7	-	156.6 C	-	154.4 C
7a	-	108.3 C	-	108.7 C
8	4.52 (2H, s)	68.1 CH_2	2.25 (3H, s)	11.1 CH_3
3-OMe	-	-	-	-
5-OMe	3.78 (3H, s)	63.0 CH_3	3.76 (3H, s)	61.5 CH_3
8-OMe	3.39 (3H, s)	58.7 CH_3	-	-
1'	3.39 (2H, m)	23.5 CH_2	3.39 (2H, br d, J = 6.9 Hz)	23.7 CH_2
2'	5.26 (1H, t, J = 7.0 Hz)	124.0 CH	5.25 (1H, t, J = 6.9 Hz)	124.1 CH
3'	-	135.4 C	-	135.2 C
4'	2.27 (2H, m)	36.0 CH_2	2.26 (2H, m)	35.8 CH_2
5'	2.34 (2H, m)	34.4 CH_2	2.35 (2H, m)	33.9 CH_2
6'	-	178.1 C	-	177.3 C
7'	1.81 (3H, s)	16.3 CH_3	1.81 (3H, s)	16.3 CH_3
6'-OMe	-	-	-	-

[a] Recorded at 400 MHz; [b] "m" means overlapped or multiplet with other signals; [c] Recorded at 100 MHz.

Table S2. ^{1}H and ^{13}C NMR data for **8–10** in CD_3OD (*δ* in ppm, *J* in Hz).

No.	**8**		**9**		**10**	
	δ_H [a,b]	δ_C [c]	δ_H [a,b]	δ_C [c]	δ_H [a,b]	δ_C [c]
1	-	173.8 C	-	173.9 C	-	173.8 C
3	5.24 (2H, s)	70.8 CH_2	5.10 (2H, s)	70.8 CH_2	5.24 (2H, s)	70.8 CH_2
3a	-	146.6 C	-	146.6 C	-	146.6 C
4	-	117.8 C	-	117.8 C	-	117.8 C
5	-	164.9 C	-	164.8 C	-	164.8 C
6	-	123.7 C	-	123.7 C	-	123.7 C
7	-	154.7 C	-	154.8 C	-	154.6 C
7a	-	107.7 C	-	107.7 C	-	107.7 C
8	2.14 (3H, s)	11.4 CH_3	2.07 (3H, s)	11.4 CH_3	2.15 (3H, s)	11.4 CH_3
5-OMe	3.76 (3H, s)	61.6 CH_3	3.71 (3H, s)	61.6 CH_3	3.76 (3H, s)	61.5 CH_3
1'	3.38 (2H, br d, *J* = 7.1 Hz)	23.6 CH_2	3.32 (2H, br d, *J* = 6.9 Hz)	23.6 CH_2	3.39 (2H, br d, *J* = 7.0 Hz)	23.6 CH_2
2'	5.27 (1H, t, *J* = 7.1 Hz)	124.4 CH	5.18 (1H, t, *J* = 6.9 Hz)	124.4 CH	5.26 (1H, t, *J* = 7.0 Hz)	124.5 CH
3'	-	135.2 C	-	135.1 C	-	135.1 C
4'	2.28 (2H, m)	36.6 CH_2	2.13 (2H, m)	36.5 CH_2	2.27 (2H, m)	36.5 CH_2
5'	2.36 (2H, m)	35.5 CH_2	2.21 (2H, m)	35.6 CH_2	2.30 (2H, m)	35.6 CH_2
6'	-	175.8 C	-	175.4 C	-	175.3 C
7'	1.83 (3H, s)	16.3 CH_3	1.73 (3H, s)	16.2 CH_3	1.82 (3H, s)	16.3 CH_3
2''	4.24 (1H, d, *J* = 5.5 Hz)	59.4 CH	4.51 (1H, dd, *J* = 5.4, 8.2 Hz)	55.5 CH	4.26 (1H, q, *J* = 7.2 Hz)	49.9 CH
3''	-	175.8 C	-	175.4 C	-	176.8 C
4''	2.10 (1H, m)	31.8 CH	2.77 (1H, dd, *J* = 8.2, 13.7 Hz); 3.03 (1H, dd, *J* = 5.4, 13.7 Hz)	38.8 CH_2	1.30 (3H, d, *J* = 7.2 Hz)	18.0 CH_3
5''	0.90 (3H, d, *J* = 6.8 Hz)	19.7 CH_3	-	138.7 C	-	-
6''	0.90 (3H, d, *J* = 6.8 Hz)	18.4 CH_3	7.12 (1H, m)	130.3 C	-	-
7''	-	-	7.19 (1H, m)	129.3 C	-	-
8''	-	-	7.14 (1H, m)	127.6 C	-	-
9''	-	-	7.19 (1H, m)	129.3 C	-	-
10''	-	-	7.12 (1H, m)	130.3 C	-	-

[a] Recorded at 400 MHz; [b] "m" means overlapped or multiplet with other signals; [c] Recorded at 100 MHz.
